# Transcriptome Sequencing, Microarray, and Proteomic Analyses Reveal Cellular and Metabolic Impact of Hepatitis C Virus Infection *In Vitro*

**DOI:** 10.1002/hep.23733

**Published:** 2010-04-23

**Authors:** Stephen D Woodhouse, Ramamurthy Narayan, Sally Latham, Sheena Lee, Robin Antrobus, Bevin Gangadharan, Shujun Luo, Gary P Schroth, Paul Klenerman, Nicole Zitzmann

**Affiliations:** 1 Oxford Glycobiology Institute, Department of Biochemistry, University of OxfordOxford, United Kingdom; 2 Peter Medawar Building for Pathogen Research, Nuffield Department of Medicine, University of OxfordOxford, United Kingdom; 3 Oxford Centre for Gene Function, Department of Human Anatomy, Physiology and Genetics, University of OxfordOxford, United Kingdom; 4 Illumina Inc.Hayward, CA

## Abstract

Hepatitis C virus (HCV) is a major cause of liver disease but the full impact of HCV infection on the hepatocyte is poorly understood. RNA sequencing (RNA-Seq) is a novel method to analyze the full transcriptional activity of a cell or tissue, thus allowing new insight into the impact of HCV infection. We conducted the first full-genome RNA-Seq analysis in a host cell to analyze infected and noninfected cells, and compared this to microarray and proteomic analyses. The combined power of the triple approach revealed that HCV infection affects a number of previously unreported canonical pathways and biological functions, including pregnane X receptor/retinoic acid receptor activation as a potential host antiviral response, and integrin-linked kinase signaling as an entry factor. This approach also identified several mechanisms implicated in HCV pathogenesis, including an increase in reactive oxygen species. HCV infection had a broad effect on cellular metabolism, leading to increases in cellular cholesterol and free fatty acid levels, associated with a profound and specific decrease in cellular glucose levels. *Conclusion:* RNA-Seq technology, especially when combined with established methods, demonstrated that HCV infection has potentially wide-ranging effects on cellular gene and protein expression. This *in vitro* study indicates a substantial metabolic impact of HCV infection and highlights new mechanisms of virus–host interaction which may be highly relevant to pathogenesis *in vivo*. (Hepatology 2010;52:443–453)

Persistent HCV infection can lead to liver disease such as hepatic steatosis, fibrosis, cirrhosis, and hepatocellular carcinoma, and is the leading indication for liver transplants in the United States.^1^ Despite this, the mechanisms of disease progression are poorly understood. With the development of the infectious hepatitis C virus (HCV) cell culture system (HCVcc),^2^-^4^ it became possible to study the entire virus infectious cycle and its effect on cellular gene and protein expression. Understanding the changes brought about by viral infection at the host cell level will allow a better insight into how current therapies work and to focus new therapeutics to the most promising areas. We used three techniques to investigate gene expression and proteomics changes following HCV infection in Huh 7.5 cells. We used the novel Solexa system (RNA-Seq) which uses whole-genome RNA sequencing technology^5^ to compare gene expression levels in infected and uninfected cells. RNA-Seq technology is likely to replace microarray technology as costs decrease.^6^, ^7^ The technology is reliable and reproducible^8^ and is increasingly used in transcriptome analysis.^9^, ^10^ However, RNA-Seq has not previously been used to study the impact of viral infection. We compared this method to conventional Affymetrix gene chip microarray, and two-dimensional gel electrophoresis (2DE)-based proteomics. In this multianalysis approach, we identified thousands of differentially expressed genes and proteins that allowed the dissection of the effects of HCV infection on a number of biofunctions and canonical pathways. These effects could have significant implications for HCV pathogenesis: if the profound cellular and metabolic modifications observed using the genotype 2 HCVcc system *in vitro* are confirmed *in vivo* and in different HCV genotypes, they could impact on disease pathogenesis and response to interferon treatment.^11^

## Materials and Methods

### Infection of Huh 7.5 cells with Jc1 HCV and X-31

Huh 7.5 cells were infected with Gt2a HCV J6CF-JFH1 (Jc1) at a multiplicity of infection (moi) of 0.02, or with X-31 influenza at moi of 1, or mock-infected with media, cultured as described^12^ and harvested when infection levels reached ≥ 90% (postinfection day 10).

#### Immunofluorescence

Huh 7.5 cells were fixed with paraformaldehyde, permeabilized with Triton X-100 and blocked with milk/phosphate-buffered saline (PBS) solution. Cells were subsequently incubated with anti-HCV core primary antibody (Cambridge Biosciences), followed by anti-mouse fluorescein isothiocyanate (Sigma). Each step was followed by PBS washes.

#### DNA Microarray Analysis

When HCV infection levels reached ≥ 90% total RNA was extracted from four infected and four noninfected replicates of Huh 7.5 cells, using the RNAeasy Mini Kit (Qiagen). Samples were prepared using the Affymetrix GeneChip WT sense target labeling and control reagents kit, and hybridized to the Affymetrix GeneChip Human Gene 1.0 ST Array containing 28,869 well-annotated genes. Chips were scanned on an Affymetrix Fluidics Station 450 and Scanner 3000.

Arrays were PLIER normalized and genes clustered in GeneSpring GX 9 (Agilent) using a Condition Tree and a Spearman correlation. Huh 7.5 cells were clustered into HCV infected and uninfected groups.

Differentially expressed genes were identified using a Welch *t* test with a *P* value cut off of ≤0.05 and a fold-change difference between treatments of ≥1.5. Gene interaction networks and canonical pathways were analyzed using Ingenuity Pathways Analysis (IPA).^13^

#### RNA-Seq Analysis

RNA was extracted from HCV infected and noninfected cells in the same way as for the microarray experiment. The poly-A containing messenger RNA molecules were purified using poly-T oligo-attached magnetic beads (Invitrogen). The messenger RNA was fragmented using divalent cations under elevated temperature (Ambion), and copied into first-strand complementary DNA (cDNA) using reverse transcriptase and random hexamer primers. Second strand cDNA synthesis was carried out using DNA polymerase I and RNase H. The cDNA fragments were prepared for sequencing on the Illumina Genome Analyzer using the Genomic DNA sequencing Sample Prep Kit (Illumina).

The analyzer identified gene names backed up by a count of the number of times it appears. The number of counts and the Illumina counting tool determines fold-changes between the different samples. For samples with a fold-change of 1.5-2, we used a cutoff of 50 counts, for fold-change of >2 we used a cutoff of >15 counts and >8 counts for a fold-change >4. Gene interactions were analyzed with IPA.^13^

#### Proteomic Analysis

Sample analyses from HCV-infected (≥90%) and uninfected Huh 7.5 cells were analyzed using 2DE (n = 4) as previously detailed,^14^ except a 1.5-fold cutoff was used. Protein spots of interest were excised and digested in-gel. Tryptic peptides were eluted and analyzed by a Micromass Q-ToF liquid chromatography tandem mass spectrometry (LC-MS/MS) system (Micromass). Spectra processed using ProteinLynx Global Server (Waters) generated “.pkl” files which were searched against SwissProt version 56.9 using Mascot Daemon version 2.1 (Matrix Science). Searches were restricted to human taxonomy (20402 sequences) with carbamideomethyl cysteine as a fixed, and oxidized methionine as a variable modification. For confident protein identification, peptide ion cutoffs were chosen to include peptides showing identity or extensive homology (*P* < 0.05), and all data were checked manually.

#### Detection of Sjogren Syndrome Antigen B

Huh 7.5 cells were infected with HCV as above and infected and noninfected cells were lysed with sample buffer.^14^ Equal amounts of protein were loaded onto a 4%-12% precast sodium dodecyl sulfate polyacrylamide gel electrophoresis gel (Invitrogen); after western blotting, the presence of Sjogren syndrome antigen B (SSB) was probed for using mouse anti-SSB (Abnova).

#### Effect of AY9944 on HCV

Huh 7.5 cells were pretreated for 8 hours in six-well plates with or without AY9944 trans-1,4-bis(2-chlorobenzylaminomethyl)cyclohexane, (Calbiochem) at 2.5, 1.25, and 0.6125 μM. After 8 hours, the cells were infected with HCV at moi = 0.2 and grown in the continued presence of the inhibitor for 3 days, after which infected cells were detected by immunofluorescence and foci were counted.

#### Glucose Assay

Cells were infected as above with either HCV or with influenza virus for 24 hours. HCV-infected and mock-infected cells were also subjected to 3 days treatment with 100 or 1000 IU/mL interferon (Sigma). Cells were harvested by trypsinization when infection levels reached ≥ 90%, and washed with PBS. Cells were lysed in radioimmunoprecipitation assay buffer and centrifuged for 5 minutes at 13500 g. The supernatant was collected and glucose determined using the Glucose assay kit (Sigma), and normalized to cellular protein content.

#### Free Fatty Acid and Cholesterol Assay

Cells were infected as above and when HCV infection levels reached ≥ 90% cells were harvested by trypsinization and washed with PBS. Cells were lysed in Triton X-100 in PBS and centrifuged for 10 minutes at 13500 g. The free fatty acid content of the supernatant was determined using the Free Fatty Acids, Half Micro Test Kit (Roche) and normalized to cellular protein content. Cholesterol content was determined using the Amplex Red cholesterol assay (Invitrogen).

#### Measurement of Reactive Oxygen Species

Huh 7.5 cells were infected with HCV at moi of 0.02 for 1, 4, 24, and 48 hours, and 8 and 10 days. Mock-infected cells were prepared for the same time points. After harvesting, 50,000 cells were resuspended in 5 μM 2′,7′-dichlorofluorescein diacetate (Sigma), and incubated for 1 hour at 37°C in the dark. Fluorescence was measured on a NOVOstar plate reader, at excitation/emission filter wavelengths of 485 nm/530 nm.

## Results

### HCV-Induced Cellular Responses Analyzed by Microarray, RNA-Seq, and Proteomic Analyses

HCV (Jc1; genotype 2a) infection of Huh 7.5 cells was monitored by immunofluorescence microscopy. When infection levels reached ≥ 90%, the cells were harvested for microarray, RNA-Seq, and proteomics analyses.

**Fig. 1:**
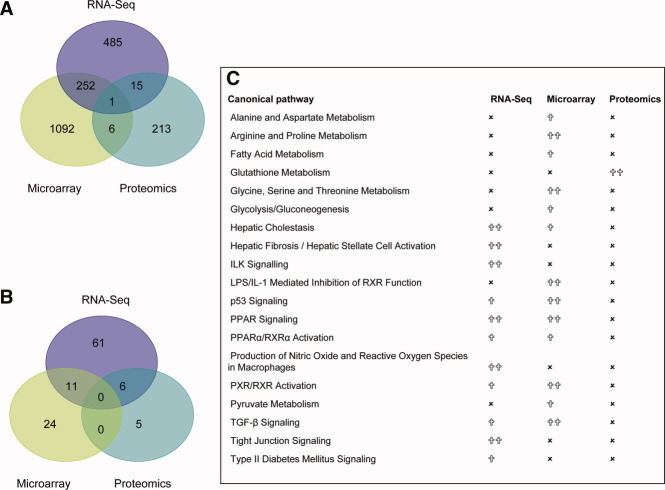
Comparative analysis of the number of genes and proteins found using RNA-Seq, microarray or proteomics analysis. (A) Numbers of genes and proteins identified by each method of analysis to be affected by HCV infection. Microarray analysis identified 1351 genes, RNA-Seq identified 753 genes and proteomics analysis identified 235 proteins (a full list can be seen in Supporting Fig. 1). (B) Analysis of the identified genes and proteins found in each study using IPA software determined the effect of HCV on a number of canonical pathways. The degree of crossover between the methods of analysis and canonical pathways identified using a cut off value of 1.5-fold change are identified in (B) and a selected number are shown in (C) (a full list can be seen in Supporting Fig. 1). † Indicates significance at the < 0.05 level. †† Indicates significance at the < 0.01% level. × Indicates not found.

**Fig. 2:**
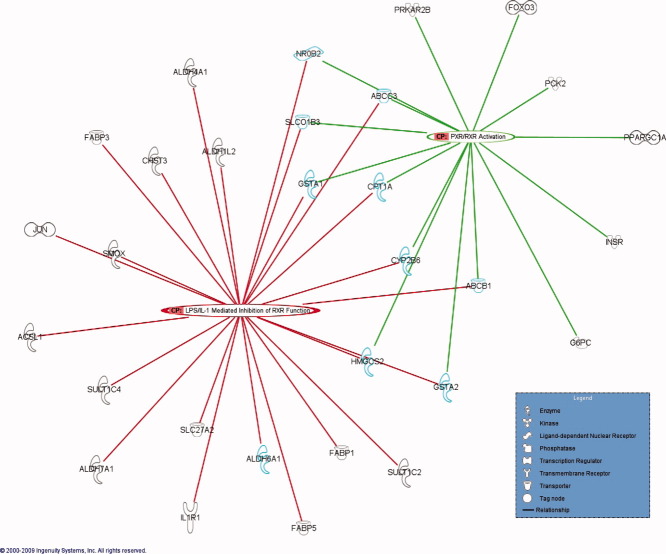
Genes identified by microarray analysis to be involved in PXR/RXR activation (*P* < 0.005) and LPS/IL-1 mediated inhibition of RXR (*P* < 0.005). Genes involved in both pathways are highlighted in blue. Actual fold changes are shown in Supporting Fig. 1.

**Fig. 3:**
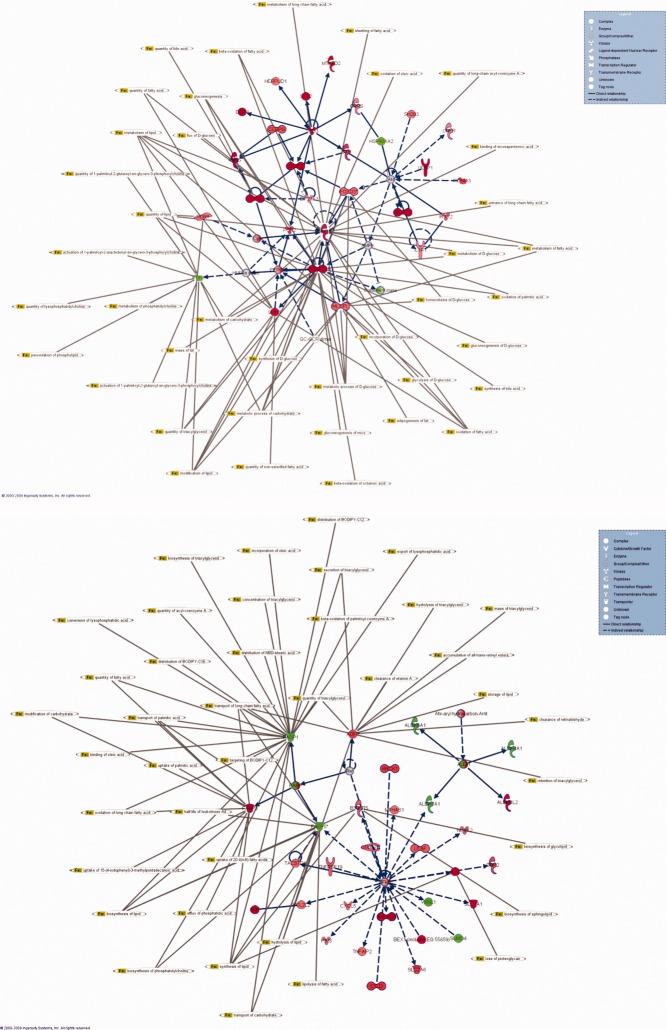
Networks of connecting genes compiled by IPA from the microarray analysis. Genes highlighted in red are up-regulated, those in green are down-regulated. Expression levels of gray genes are not altered. Genes connected with blue filled lines have direct links; those connected with blue dotted lines have indirect links. Functional annotations for lipid and carbohydrate metabolism are indicated by filled gray lines.

Microarray analysis identified 1351 genes, RNA-Seq identified 753 genes, and proteomics analysis identified 235 proteins which were differentially regulated in response to HCV infection. Although some overlap of genes/proteins identified by the three methods occurs, the majority of differential expression was, surprisingly, determined by a single method of analysis (Fig. 1A). IPA analysis of the genes detected by the microarray method identified 35 canonical pathways (Fig. 1B) and numerous biofunctions (Supporting Table 1). For the first time, HCV is shown to induce the differential expression of genes involved in PXR/RXR activation and LPS/IL-1 mediated inhibition of RXR function canonical pathways (Fig. 2). In addition gene networks identifying lipid and carbohydrate metabolism functions were identified (Fig. 3). By comparison; RNA-Seq gene expression analysis identified 78 canonical pathways (Fig. 1B), probably due to a lower redundancy rate in genes identified as compared to the microarray analysis. This method allowed identification of more than twice the number of canonical pathways determined by the microarray method and seven times more than by proteomics (Fig. 1B). In addition to supporting previous findings such as the impact of HCV on TGF-β signaling^15^ and hepatic fibrosis/hepatic stellate cell activation,^16^ previously unreported pathways were identified as being affected by HCV such as tight junction (TJ) signaling (Supporting Table 1). Of particular note in this pathway is the up-regulation of par-3 partitioning defective 3 homolog, which is thought to regulate TJ assembly.^17^, ^18^

**Fig. 4:**
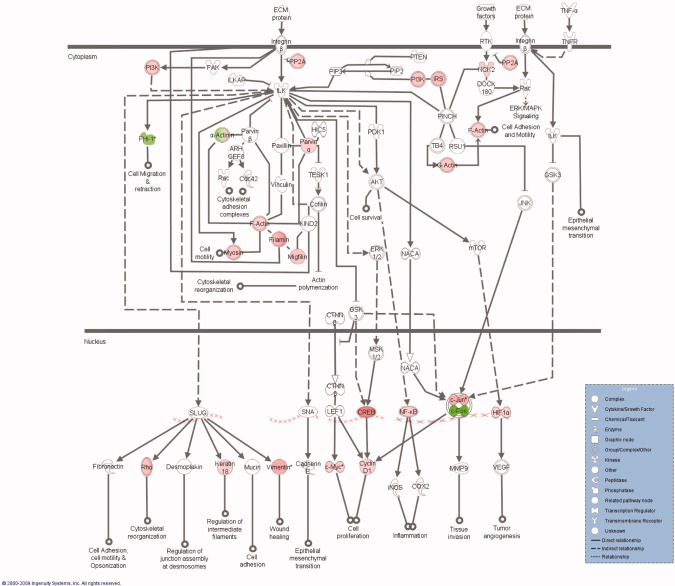
Impact of HCV infection on ILK signaling. A schematic of the ILK signaling canonical pathway produced using IPA and overlaid with genes identified by RNA-Seq and Microarray analysis. Genes highlighted in red are up-regulated, those in green are down-regulated. Gray genes are not detected to be altered.

Collating the genomic analyses can also provide further information; e.g., an additional five genes were added to the integrin-linked kinase (ILK) signaling canonical pathway (identified by RNA-Seq) by overlaying the microarray analyses (Fig. 4).

**Fig. 5:**
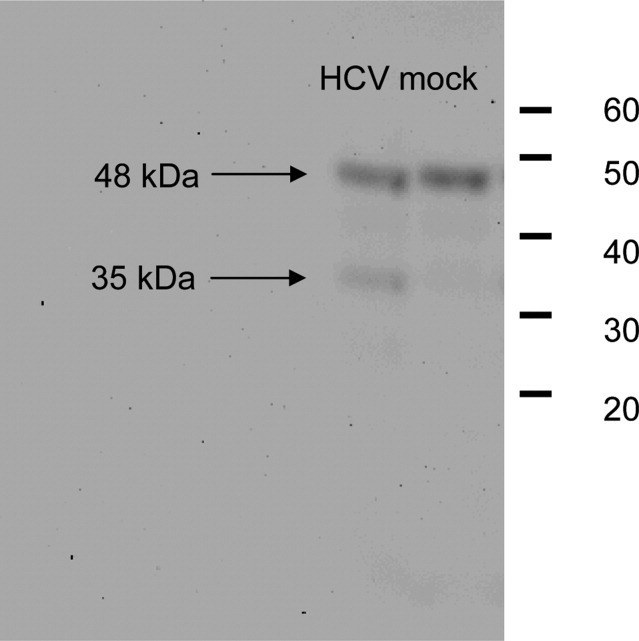
Proteomic analysis reveals the impact of HCV infection on SSB. Western blot analysis of equal amounts of protein extracted from HCV infected and mock-infected Huh 7.5 cells. Subsequent probing with a monoclonal antibody against SSB, detected the presence of a cleavage product of 35 kDa.

The 235 proteins identified by the 2DE-based proteomic study (Supporting Fig. 1) are in agreement with a previous study where changes in proteins involved in lipid metabolism, oxidative stress, and carbohydrate metabolism were observed.^19^ In addition we identified proteins involved in glutathione metabolism and numerous RNA binding proteins, including SSB, HNRPC, HNRPK, PCBP1, and PCBP2, of which SSB HNRPC and HNRPK are known to bind the untranslated regions of HCV RNA.^20^, ^21^ western blot analysis to confirm altered levels of SSB did not confirm its up-regulation by HCV. However, a cleavage fragment was detected in the HCV-infected sample only, illustrating an HCV induced change (Fig. 5).

#### Impact of HCVcc Infection on Lipid and Cholesterol Metabolism

**Fig. 6:**
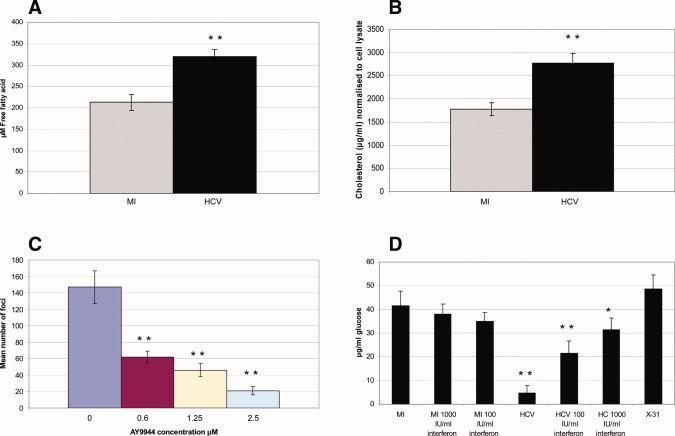
Impact of HCV on cellular metabolism. At the same time point the gene and proteomics analyses were carried out (HCV infection levels ≥ 90%) the levels of free fatty acid, cholesterol and glucose in HCV infected Huh 7.5 cells were compared to mock-infected cells and significance measured using a Student *t* test (n = 4). (A) Free fatty acid levels in HCV infected cells were significantly higher than in control cells with average values of 320 μM in infected cells compared to 215 μM within uninfected cells. (B) Cholesterol levels are higher in infected cells with average levels of 1778 μg/mL in noninfected and 2774 μg/mL in HCV infected cells. (C) To demonstrate the requirement for cholesterol, Huh 7.5 cells were infected with HCV at a moi of 0.2, at 8 hours after pretreatment with 0, 0.6, 1.25, and 2.5 μM AY9944. At 3 days after infection, cells were analyzed by immunofluorescence and foci of infected cells counted. At increasing concentrations of AY9944 significant decreases in foci were exhibited compared to mock-infected control cells (student *t* test [n = 4]). (D) At day 10 after infection, glucose levels within HCV infected Huh7.5 showed a 9-fold drop (significance Student *t* test [n = 4]) compared to noninfected cells with levels of 4.6 μg/mL compared to 41.6 μg/mL of noninfected cells. This difference is ameliorated by the addition of interferon (which has no significant effect on mock-infected cells) in a dose-dependent manner. Infection of Huh 7.5 cells with X-31 influenza has no effect on glucose levels. *Indicates significance at the <0.05 level. **Indicates significance at the < 0.01% level. MI, mock-infected.

Components of host lipid metabolism such as very low density lipoprotein and apolipoproteinB-100 are known to play key roles in HCV replication (for review, see Syed et al.^22^) and a more detailed investigation will aid our understanding of the mechanisms involved. Gene ontology analysis of the microarray and RNA-Seq data using IPA showed that biofunctions and canonical pathways associated with lipids and cholesterol are markedly affected by HCV infection (Supporting Table 1). We therefore investigated these pathways further using functional studies and biochemical assays. A significant (*P* < 0.01) increase in cholesterol and free fatty acid levels in HCV-infected Huh 7.5 cells is shown here for the first time, with cholesterol and free fatty acid levels more than a third higher in HCV-infected cells (Fig. 6). Increased levels of cholesterol are important for successful HCV replication as prophylactic use of the 3-beta-hydroxysterol Δ-7 reductase inhibitor AY9944, which prevents cholesterol synthesis,^23^ for 6 hours prior to infection, caused a reduction in HCV foci formation in a dose-dependent manner (Fig. 6). Statins have achieved similar effects *in vitro*.^24^, ^25^

In addition, we noted up-regulation of the pregnane X receptor (PXR)/retinoic acid receptor (RXR) ligand activation signaling pathway (identified by microarray and RNA-Seq data analysis, Fig. 2). This is of interest because PXR ligands may ameliorate human diseases such as cholestatic liver disease.^26^

#### Impact of HCVcc Infection on Cellular Glucose Metabolism

Pathway analysis of the microarray data showed perturbation of the glycolysis and gluconeogenesis canonical pathways after HCV infection, with nine genes being differentially expressed. The SLC2A4RG gene (microarray), which induces the gene responsible for expression of the glucose transporter SLC2A4, is down-regulated 2.3-fold and another glucose transporter SLC2A8 (microarray, implicated in glucose homeostasis^27^) is up-regulated 1.9-fold. We therefore analyzed the glucose levels in HCV-infected Huh 7.5 cells, which were found to be nine-fold lower compared to noninfected cells (Fig. 6D). Glucose levels recovered in a dose-dependent manner, after interferon treatment for 3 days. In contrast, infection of Huh 7.5 cells with influenza virus (type A, strain X-31; infection level close to 100%), or treatment of uninfected cells with interferon, had no impact on glucose levels.

#### Impact of HCVcc on Reactive Oxygen Species and Oxidative Stress

**Fig. 7:**
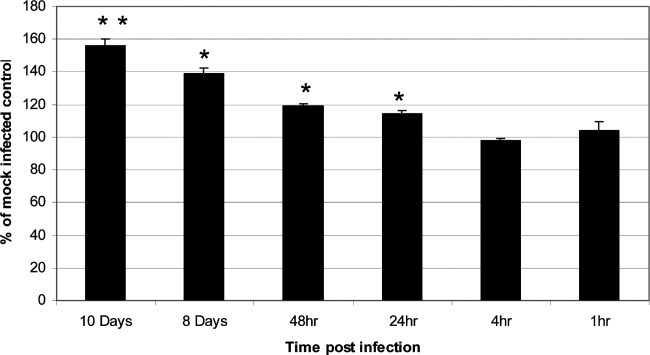
Measurement of ROS in the HCVcc system. Huh 7.5 cells were preinfected with HCV for 1, 4, 24, and 48 hours and 8 and 10 days. ROS were detected with 2′,7′-dichlorofluorescein diacetate. All regimes were compared to similarly treated mock-infected Huh 7.5 cells. *Indicates significance at the <0.05 level. **Indicates significance at the <0.01% level.

HCV infection is characterized by increased levels of markers of oxidative stress, and lipid peroxidation products are increased in serum and liver specimens from patients.^28^ Analysis of HCV infection in the microarray experiment identified nine up-regulated metallothionein and five glutathione-related genes, the RNA-Seq experiment identified superoxide dismutase 3, and the proteomics analysis identified altered expression of superoxide dismutase, which are likely to be involved in alleviating oxidative stress, and counteracting production of lipid peroxidation and/or reactive oxygen species (ROS).^29^, ^30^ Measurement of ROS using 2′,7′-dichlorofluorescein diacetate showed a significant increase (*P* < 0.05) 48 hours after HCV infection, which increased further with time after infection; 10 days after infection (when gene and proteomics analyses were conducted) ROS levels were 56% (*P* < 0.01) higher in HCV-infected cells (Fig. 7).

## Discussion

The RNA-Seq analysis, in combination with microarray and proteomics approaches, highlights hitherto unreported cellular responses to HCV infection at the gene level, adding to the recently published data by Walters et al.^31^ The RNA-Seq approach, based on new technological advances in massively parallel sequencing, has a number of advantages over conventional microarray techniques. The most important of these is that no prior selection of RNA is required, as is inherent in the use of probe/hybridization-based assays. Thus, an unbiased analysis of the total transcriptome can be performed, and the specific impact of HCVcc infection on Huh 7.5 cells *in vitro* could be analyzed. The limitations of the technique are the requirements for large quantities of high-quality RNA, the current cost and requirement for specialist sequencing equipment, and management of the huge data load. However, rapidly emerging data from this new field point to important potential benefits in terms of improved detection.^6^, ^9^, ^32^

By collating the results from the different techniques it is possible to expand the information regarding the effect of HCV on gene and protein expression, increasing our chances of identifying potentially important genes and pathways. One such example is the observed increase of genes in the ILK signaling canonical pathway from 25 to 30. HCV induction of the ILK signaling canonical pathway (reported here for the first time) is likely to trigger actin rearrangement.^33^ Such cytoskeleton regulation is postulated to be important for HCV replication,^34^, ^35^ perhaps allowing viral movement to the tight junctions in a similar manner to Coxsackievirus.^36^, ^37^ The RNA-Seq analysis identified the tight junction signaling canonical pathway (Supporting Fig. 1), increasingly thought to be important in HCV entry and transmission.^38^, ^39^ A number of other canonical pathways affected by HCV infection including LPS/IL-1 mediated inhibition of RXR function, and PXR/RXR activation are reported here for the first time. HCV core protein has been shown to bind RXRα resulting in the up-regulation of certain lipid metabolism enzymes. As HCV replication is linked to increased lipid metabolism, this potentially increases viral replication.^40^ The “LPS/IL-1 mediated inhibition of RXR function” canonical pathway identified by IPA may also point toward a novel host antiviral response, as inhibition of RXR function could impair cholesterol and lipid metabolism^41^ required for viral replication.

The combined analysis used in this study indicates that HCV infection is likely to cause disruption of glycolysis, gluconeogenesis and lipid metabolism. This is supported by the finding that interference of the cholesterol biosynthesis pathway causes a drop in HCV infectivity and replication. In addition, following HCV infection, cellular glucose levels are significantly decreased and both free fatty acid and cholesterol levels are significantly increased. An increase in cholesterol metabolism may lead to increased glycolysis and gluconeogenesis which could result in increased acetyl-CoA needed for cholesterol production, leading to decreased glucose levels and increased amino acid metabolism, perhaps for gluconeogenesis. Furthermore, free fatty acid metabolism is also increased. ACSL1, which converts free long-chain fatty acids into acetyl-CoA precursors, as well as CPT1A and CPT1B, which transport fatty acids across the outer mitochondrial membrane for subsequent production of acetyl-CoA, are up-regulated 2.5-fold, 2.3-fold, and 1.6-fold, respectively, which could support viral replication by increasing cholesterol production.

HCV suppresses cellular glucose uptake by down-regulation of surface expression of glucose transporters GLUT1 and GLUT2.^42^ Down-regulation of GLUT4 (SLC2A4) has been identified by Walters et al.^31^ and confirmed in this study, and we further identify down-regulation of GLUT3 (SLC2A3), indicating that it may be involved in a similar manner. Although infected cells may be attempting to counter the HCV-induced low cellular glucose levels by increasing gluconeogenesis, they appear to be unable to counteract both increased glycolysis as well as decreased glucose transport.

This is the first time ROS levels have been quantified in the infectious HCVcc system. The induction of ROS depends on the time after infection and on infection levels. ROS may contribute to the development of hepatocellular carcinoma due to triggering double-stranded DNA breaks^43^ and could also lead to fibrosis via induction of TGF-β.^44^ The superoxide dismutase protein identified by proteomics, and superoxide dismutase 3, whose up-regulated gene was identified by RNA-Seq analysis, as well as the metallothione and glutathione genes identified in the microarray analyses, could potentially protect against HCV-induced ROS and oxidative stress as well as suppress HCV RNA replication in a manner similar to antioxidants.^45^, ^46^

HCV induces differential expression of several RNA binding proteins including SSB (Supporting Table 1). SSB interacts with the HCV IRES in the 5′ untranslated region of the viral RNA, an area important for the translation of the HCV genome.^47^, ^48^ Western blot analysis probing for SSB indicates that HCV induces cleavage of SSB (Supporting Table 1). Using an HCV replicon system, Romero et al. demonstrate that granzyme H (from cytotoxic-lymphocyte granules) exerts an antiviral effect by cleaving SSB, thereby preventing IRES translation of the HCV genome.^49^ We do not know which factor is responsible for cleaving SSB in our study (HCVcc in the absence of cytotoxic lymphocyte granules), but it may hint at a novel host mediated antiviral response.

We have only highlighted a key subset of the differentially regulated genes and proteins. Further analysis of the data sets will contribute to our understanding of virus-host interplay in HCV, and use of the RNA-Seq technology in combination with microarray and proteomics techniques could have a major impact in other infectious diseases. It will also be of great interest to compare and confirm our findings with other HCV infectious systems and genotypes as they become available. This study was performed using the most robust *in vitro* system available at the time comprising an HCV genotype 2 infected human hepatoma cell line, which has been widely used in similar studies.^31^, ^42^, ^50^ However, it will be important to define the significance of the transcriptional changes, especially in relation to different host and viral genotypes in similar studies and *in vivo*.

## Abbreviations

2DE: two-dimensional gel electrophoresis
AY9944: trans-1,4-bis(2-chlorobenzylaminomethyl)cyclohexane
cDNA: complementary DNA
HCVcc: hepatitis C virus cell culture system
PXR: pregnane X receptor
RNA-Seq: RNA sequencing
ROS: reactive oxygen species
RXR: retinoic acid receptor
SSB: Sjogren syndrome antigen B
