# Supplementary material for: Transcriptome Sequencing, Microarray, and Proteomic Analyses Reveal Cellular and Metabolic Impact of Hepatitis C Virus Infection In Vitro
Source: Hepatology. 2010 Apr 23;52(2):443–53. doi: 10.1002/hep.23733 (PMC3427885; doi:10.1002/hep.23733)
Supplement: Supplementary file 1 [file hep0052-0443-SD1.doc]

**Supporting Figure 1**

Affymetrix Microarray Data

| **Gene Symbol** | **Fold change** | **Gene Title** | **Cluster Id** |
| --- | --- | --- | --- |
| ANKRD1 | 14.9 | ankyrin repeat domain 1 (cardiac muscle) | 7934979 |
| RASGEF1B | 10.2 | RasGEF domain family, member 1B | 8101304 |
| AREG | 9.9 | amphiregulin (schwannoma-derived growth factor) | 8095736 |
| PLA2G4C | 9.6 | phospholipase A2, group IVC (cytosolic, calcium-independent) | 8037970 |
| AREG | 9.3 | amphiregulin (schwannoma-derived growth factor) | 8095744 |
| INHBE | 7.8 | inhibin, beta E | 7956426 |
| QPCT | 7.4 | glutaminyl-peptide cyclotransferase (glutaminyl cyclase) | 8041508 |
| THBS1 | 6.3 | thrombospondin 1 | 7982597 |
| MT2A | 6.2 | metallothionein 2A | 7995783 |
| IL8 | 5.9 | interleukin 8 | 8095680 |
| NCF2 | 5.7 | neutrophil cytosolic factor 2 (65kDa, chronic granulomatous disease, autosomal 2) | 7922773 |
| F2RL1 | 5.5 | coagulation factor II (thrombin) receptor-like 1 | 8106403 |
| PLA1A | 5.5 | phospholipase A1 member A | 8081890 |
| RCN1 | 5.5 | reticulocalbin 1, EF-hand calcium binding domain | 7939120 |
| NOSTRIN | 5.4 | nitric oxide synthase trafficker | 8046099 |
| PRSS23 | 5.3 | protease, serine, 23 | 7942957 |
| HSD17B14 | 5.3 | hydroxysteroid (17-beta) dehydrogenase 14 | 8038213 |
| PHLDA1 | 5.0 | pleckstrin homology-like domain, family A, member 1 | 7965040 |
| NPPB | 4.8 | natriuretic peptide precursor B | 7912520 |
| TP53INP1 | 4.7 | tumor protein p53 inducible nuclear protein 1 | 8151890 |
| HSPB8 | 4.6 | heat shock 22kDa protein 8 | 7959102 |
| LOC729642 | 4.6 | hypothetical protein LOC729642 | 8026490 |
| SERPINE2 | 4.4 | serpin peptidase inhibitor, clade E (nexin, plasminogen activator inhibitor type 1), member 2 | 8059376 |
| MT1A | 4.4 | metallothionein 1A | 8162531 |
| STC2 | 4.4 | stanniocalcin 2 | 8115851 |
| LOC284417 | 4.1 | hCG1651476 | 8039453 |
| GDF15 | 4.1 | growth differentiation factor 15 | 8027002 |
| TUBA1A | 4.0 | tubulin, alpha 1a | 7963054 |
| ANXA3 | 4.0 | annexin A3 | 8095986 |
| MT1X | 3.9 | metallothionein 1X | 7995838 |
| MT1A | 3.9 | metallothionein 1A1 | 7995806 |
| LTBP1 | 3.8 | latent transforming growth factor beta binding protein 1 | 8041383 |
| ZNF114 | 3.8 | zinc finger protein 114 | 8030002 |
| NUPR1 | 3.8 | nuclear protein 1 /// nuclear protein 1 (NUPR1), transcript variant 1, mRNA | 7897801 |
| ATF3 | 3.7 | activating transcription factor 3 | 8000574 |
| DDIT4 | 3.7 | DNA-damage-inducible transcript 4 | 7909610 |
| HDAC9 | 3.7 | histone deacetylase 9 | 7928308 |
| UAP1L1 | 3.7 | UDP-N-acteylglucosamine pyrophosphorylase 1-like 1 | 8131631 |
| SV2A | 3.7 | synaptic vesicle glycoprotein 2A | 8159554 |
| DDIT3 | 3.6 | DNA-damage-inducible transcript 3 | 7997188 |
| TMEM140 | 3.6 | transmembrane protein 140 | 7919645 |
| PRNP | 3.6 | prion protein (p27-30) (Creutzfeldt-Jakob disease,) | 7964460 |
| S100P | 3.5 | S100 calcium binding protein P | 8136388 |
| DAGLA | 3.5 | diacylglycerol lipase, alpha | 8060758 |
| STX3 | 3.5 | syntaxin 3 | 7915612 |
| SLC34A2 | 3.4 | solute carrier family 34 (sodium phosphate), member 2 | 8093950 |
| VLDLR | 3.4 | very low density lipoprotein receptor | 7940508 |
| TRIB3 | 3.3 | tribbles homolog 3 (Drosophila) | 7940191 |
| IL32 | 3.3 | interleukin 32 | 8152819 |
| ULBP1 | 3.2 | UL16 binding protein 1 | 7915592 |
| MAP1B | 3.2 | microtubule-associated protein 1B | 8094441 |
| LAMA3 | 3.2 | laminin, alpha 3 | 8154100 |
| SULT1C2 | 3.1 | sulfotransferase family, cytosolic, 1C, member 2 | 8060344 |
| MT1E | 3.1 | metallothionein 1E | 7992828 |
| TMEM144 | 3.1 | transmembrane protein 144 | 8122724 |
| GTPBP2 | 3.1 | GTP binding protein 2 | 8106098 |
| SLC1A4 | 3.1 | solute carrier family 1 (glutamate/neutral amino acid transporter), member 4 | 8020551 |
| CYR61 | 3.0 | cysteine-rich, angiogenic inducer, 61 | 8044212 |
| TMC7 | 3.0 | transmembrane channel-like 7 | 7995797 |
| CREB5 | 2.9 | cAMP responsive element binding protein 5 | 7984257 |
| FER1L3 | 2.9 | fer-1-like 3, myoferlin (C. elegans) | 8098041 |
| PPARGC1A | 2.9 | peroxisome proliferator-activated receptor gamma, coactivator 1 alpha | 8126629 |
| GADD45A | 2.9 | growth arrest and DNA-damage-inducible, alpha | 8042310 |
| LARP6 | 2.9 | La ribonucleoprotein domain family, member 6 | 7990564 |
| VEPH1 | 2.9 | ventricular zone expressed PH domain homolog 1 (zebrafish) | 7902687 |
| FSTL1 | 2.9 | follistatin-like 1 | 7993588 |
| TCP11L2 | 2.9 | t-complex 11 (mouse)-like 2 | 8131996 |
| FABP3 | 2.9 | fatty acid binding protein 3, muscle and heart (mammary-derived growth inhibitor) | 7935058 |
| KIAA0746 /// KIAA0746 | 2.8 | KIAA0746 protein /// KIAA0746 protein (KIAA0746), mRNA | 8099633 |
| GPR137B | 2.8 | G protein-coupled receptor 137B | 7902227 |
| RAB32 | 2.8 | RAB32, member RAS oncogene family | 8092185 |
| SESN2 | 2.8 | sestrin 2 | 7990080 |
| SLC7A11 | 2.8 | solute carrier family 7, (cationic amino acid transporter, y+ system) member 11 | 8091678 |
| ASNS | 2.8 | asparagine synthetase | 8089835 |
| F2RL2 | 2.8 | coagulation factor II (thrombin) receptor-like 2 | 7958262 |
| DAB2 | 2.8 | disabled homolog 2, mitogen-responsive phosphoprotein (Drosophila) | 8093152 |
| C6orf115 | 2.8 | chromosome 6 open reading frame 115 | 7997192 |
| MT1H | 2.8 | metallothionein 1H | 8124394 |
| ARHGEF2 | 2.8 | rho/rac guanine nucleotide exchange factor (GEF) 2 | 7914342 |
| CAPN2 | 2.8 | calpain 2, (m/II) large subunit | 7925318 |
| ALDH1L2 | 2.7 | aldehyde dehydrogenase 1 family, member L2 | 8099721 |
| NRCAM | 2.7 | neuronal cell adhesion molecule / | 8060997 |
| MRAS | 2.7 | muscle RAS oncogene homolog | 7910680 |
| DKFZp686O24166 | 2.7 | DKFZp686O24166 | 8122554 |
| PFKFB2 | 2.7 | 6-phosphofructo-2-kinase/fructose-2,6-biphosphatase 2 | 7899436 |
| RRAGD | 2.7 | Ras-related GTP binding D | 8102800 |
| BMF /// BMF | 2.7 | Bcl2 modifying factor /// Bcl2 modifying factor (BMF), transcript variant 3, mRNA | 8141150 |
| PLK2 | 2.7 | polo-like kinase 2 (Drosophila) | 8112731 |
| MT1L | 2.7 | metallothionein 1L (gene/pseudogene) | 8111772 |
| MTHFD2 | 2.7 | methylenetetrahydrofolate dehydrogenase (NADP+ dependent) 2, methenyltetrahydrofolate cyclohydrolase | 8122336 |
| RRAGC | 2.7 | Ras-related GTP binding C | 8140984 |
| TMPRSS9 | 2.7 | transmembrane protease, serine 9 | 7995829 |
| C6orf145 | 2.7 | chromosome 6 open reading frame 145 | 7924069 |
| HABP2 | 2.7 | hyaluronan binding protein 2 | 7920877 |
| LOC442098 | 2.7 | similar to Bifunctional methylenetetrahydrofolate dehydrogenase/cyclohydrolase, mitochondrial precursor | 7909967 |
| C12orf49 | 2.7 | chromosome 12 open reading frame 49 | 8094240 |
| CHAC1 | 2.7 | ChaC, cation transport regulator homolog 1 (E. coli) | 7965979 |
| SERPINA3 | 2.7 | serpin peptidase inhibitor, clade A (alpha-1 antiproteinase, antitrypsin), member 3 | 8142270 |
| OR2A20P | 2.7 | olfactory receptor, family 2, subfamily A, member 20 pseudogene | 8082965 |
| TNFRSF12A | 2.7 | tumor necrosis factor receptor superfamily, member 12A | 7938702 |
| PMAIP1 | 2.6 | phorbol-12-myristate-13-acetate-induced protein 1 | 7909285 |
| GCNT4 | 2.6 | glucosaminyl (N-acetyl) transferase 4, core 2 (beta-1,6-N-acetylglucosaminyltransferase) | 8128123 |
| ENO2 | 2.6 | enolase 2 (gamma, neuronal) | 7987454 |
| GCNT3 | 2.6 | glucosaminyl (N-acetyl) transferase 3, mucin type | 8112202 |
| FAM129A | 2.6 | family with sequence similarity 129, member A | 7995793 |
| SYT11 | 2.6 | synaptotagmin XI | 8042830 |
| NEU1 | 2.6 | sialidase 1 (lysosomal sialidase) | 7915160 |
| TMEM27 | 2.6 | transmembrane protein 27 | 8024467 |
| NEU1 | 2.6 | sialidase 1 (lysosomal sialidase) | 8123678 |
| NEU1 | 2.6 | sialidase 1 (lysosomal sialidase) | 7930561 |
| TUBB2B | 2.6 | tubulin, beta 2B | 8084064 |
| GPR37 | 2.6 | G protein-coupled receptor 37 (endothelin receptor type B-like) | 7966738 |
| OR2A20P | 2.5 | olfactory receptor, family 2, subfamily A, member 20 pseudogene | 7982868 |
| BHLHB2 | 2.5 | basic helix-loop-helix domain containing, class B, 2 | 7976496 |
| PKIB | 2.5 | protein kinase (cAMP-dependent, catalytic) inhibitor beta | 8136983 |
| ANK2 | 2.5 | ankyrin 2, neuronal transcript variant 2, mRNA | 7992789 |
| JUN | 2.5 | jun oncogene | 8021470 |
| ITPR3 | 2.5 | inositol 1,4,5-triphosphate receptor, type 3 | 8112668 |
| VIM | 2.5 | vimentin | 7953532 |
| SLC7A1 | 2.5 | solute carrier family 7 (cationic amino acid transporter, y+ system), member 1 | 7984001 |
| CTSL1 | 2.5 | cathepsin L1 | 7922846 |
| KIF5C | 2.5 | kinesin family member 5C /// kinesin family member 5C (KIF5C), mRNA | 7906061 |
| PPP1R15A | 2.5 | protein phosphatase 1, regulatory (inhibitor) subunit 15A | 7955441 |
| UBD | 2.5 | ubiquitin D | 8178676 |
| MOSPD1 | 2.5 | motile sperm domain containing 1 | 7917726 |
| DUSP1 | 2.5 | dual specificity phosphatase 1 | 8171472 |
| UBD | 2.5 | ubiquitin D | 8179851 |
| ACSL1 | 2.5 | acyl-CoA synthetase long-chain family member 1 | 8125139 |
| CYP2B6 | 2.5 | cytochrome P450, family 2, subfamily B, polypeptide 6 | 8123651 |
| ANXA1 | 2.5 | annexin A1 | 8142687 |
| CSF1 | 2.4 | colony stimulating factor 1 (macrophage) | 8143629 |
| BEX1 | 2.4 | brain expressed, X-linked 1 | 8077441 |
| KLF4 | 2.4 | Kruppel-like factor 4 (gut) | 8121768 |
| TAGLN | 2.4 | transgelin | 8096959 |
| LPIN1 | 2.4 | lipin 1 | 7916609 |
| SPAG1 | 2.4 | sperm associated antigen 1 | 8118734 |
| ABLIM3 | 2.4 | actin binding LIM protein family, member 3 | 7926368 |
| --- | 2.4 | --- | 7970810 |
| CYP1A1 | 2.4 | cytochrome P450, family 1, subfamily A, polypeptide 1 | 8156228 |
| SULT1C4 | 2.4 | sulfotransferase family, cytosolic, 1C, member 4 | 8040419 |
| ALPK3 | 2.4 | alpha-kinase 3 | 8045637 |
| RHOU | 2.4 | ras homolog gene family, member U | 8030128 |
| MT1JP | 2.4 | metallothionein 1J (pseudogene) | 8178295 |
| DHX32 | 2.4 | DEAH (Asp-Glu-Ala-His) box polypeptide 32 | 8175288 |
| BHLHB3 | 2.4 | basic helix-loop-helix domain containing, class B, 3 | 8115831 |
| MR1 | 2.3 | major histocompatibility complex, class I-related | 8124650 |
| STBD1 | 2.3 | starch binding domain 1 | 8009476 |
| CPT1A | 2.3 | carnitine palmitoyltransferase 1A (liver) | 8103951 |
| RNF19A | 2.3 | ring finger protein 19A | 8028963 |
| GADD45B | 2.3 | growth arrest and DNA-damage-inducible, beta | 8155849 |
| OPTN | 2.3 | optineurin | 8130622 |
| ACSS2 | 2.3 | acyl-CoA synthetase short-chain family member 2 | 8071593 |
| ASAH1 | 2.3 | N-acylsphingosine amidohydrolase (acid ceramidase) 1 | 7903786 |
| SLC41A2 | 2.3 | solute carrier family 41, member 2 | 8174201 |
| CPEB4 | 2.3 | cytoplasmic polyadenylation element binding protein 4 | 8163002 |
| AHR | 2.3 | aryl hydrocarbon receptor | 7944082 |
| RASD1 | 2.3 | RAS, dexamethasone-induced 1 | 7996260 |
| EDARADD | 2.3 | EDAR-associated death domain | 8040340 |
| LOXL4 | 2.3 | lysyl oxidase-like 4 | 8147661 |
| KLF11 | 2.3 | Kruppel-like factor 11 | 8109093 |
| LAD1 | 2.3 | ladinin 1 | 7902439 |
| ELF3 | 2.3 | E74-like factor 3 (ets domain transcription factor, epithelial-specific ) | 7990391 |
| C17orf28 | 2.3 | chromosome 17 open reading frame 28 | 8044225 |
| CLIP4 | 2.3 | CAP-GLY domain containing linker protein family, member 4 | 7985620 |
| ELOVL7 | 2.3 | ELOVL family member 7, elongation of long chain fatty acids (yeast) | 8107857 |
| FAM134B | 2.3 | family with sequence similarity 134, member B | 7910387 |
| FLJ10815 | 2.3 | amino acid transporter | 8108301 |
| LSP1 | 2.3 | lymphocyte-specific protein 1 pseudogene lymphocyte-specific protein 1 | 7995803 |
| KLF6 | 2.3 | Kruppel-like factor 6 | 7909877 |
| CLCN7 | 2.3 | chloride channel 7 | 8124413 |
| MST1 | 2.3 | macrophage stimulating 1 (hepatocyte growth factor-like) | 7936949 |
| C10orf10 | 2.3 | chromosome 10 open reading frame 10 | 8094911 |
| IDS | 2.3 | iduronate 2-sulfatase (Hunter syndrome) | 7961891 |
| MT1F | 2.2 | metallothionein 1F | 7973745 |
| FLNC /// FLNC | 2.2 | filamin C, gamma (actin binding protein 280) /// filamin C, gamma (actin binding protein 280) (FLNC), mRNA | 7907893 |
| OTUD1 | 2.2 | OTU domain containing 1 | 8095826 |
| --- | 2.2 | ... | 7949971 |
| PCK2 | 2.2 | phosphoenolpyruvate carboxykinase 2, nuclear gene encoding mitochondrial protein, transcript variant 2, | 8152041 |
| TMSL1 /// TMSL2 /// TMSL6 /// TMSL3 /// TMSB4X | 2.2 | thymosin-like 1 /// thymosin-like 2 /// thymosin-like 6 /// thymosin-like 3 /// thymosin, beta 4, X-linked | 8024485 |
| FNIP1 | 2.2 | folliculin interacting protein 1 | 7926239 |
| ADM2 | 2.2 | adrenomedullin 2 | 8062041 |
| ABCC3 | 2.2 | ATP-binding cassette, sub-family C (CFTR/MRP), member 3 | 7951091 |
| FILIP1L | 2.2 | filamin A interacting protein 1-like | 8149534 |
| C1orf34 | 2.2 | chromosome 1 open reading frame 34 | 7965964 |
| MICAL2 | 2.2 | microtubule associated monoxygenase, calponin and LIM domain containing 2 | 8110055 |
| SLC3A2 | 2.2 | solute carrier family 3 (activators of dibasic and neutral amino acid transport), member 2 | 7928907 |
| UBASH3B | 2.2 | ubiquitin associated and SH3 domain containing, B | 8131614 |
| CYP2B7P1 | 2.2 | cytochrome P450, family 2, subfamily B, polypeptide 7 pseudogene 1 | 8013112 |
| TSC22D3 | 2.2 | TSC22 domain family, member 3 | 8135378 |
| CTH | 2.2 | cystathionase (cystathionine gamma-lyase) | 7910694 |
| RIT1 | 2.2 | Ras-like without CAAX 1 | 7939559 |
| MIRN21 | 2.2 | microRNA 21 | 7935553 |
| SLC44A3 | 2.2 | solute carrier family 44, member 3 | 8040211 |
| BMP2 | 2.2 | bone morphogenetic protein 2 | 8124430 |
| FBN1 | 2.1 | fibrillin 1 | 7923347 |
| BTG2 | 2.1 | BTG family, member 2 | 7908793 |
| DUSP8 | 2.1 | dual specificity phosphatase 8 | 8018264 |
| TINAGL1 | 2.1 | tubulointerstitial nephritis antigen-like 1 | 8162823 |
| UNC93A | 2.1 | unc-93 homolog A (C. elegans) | 8041179 |
| FBXO25 | 2.1 | --- | 7984620 |
| DOCK11 | 2.1 | dedicator of cytokinesis 11 | 8112274 |
| LRRN1 | 2.1 | leucine rich repeat neuronal 1 | 8111136 |
| TMSB4X /// TMSL1 /// TMSL2 /// TMSL6 /// TMSL3 | 2.1 | thymosin, beta 4, X-linked /// thymosin-like 1 /// thymosin-like 2 /// thymosin-like 6 /// thymosin-like 3 | 8001750 |
| ITGAV | 2.1 | integrin, alpha V (vitronectin receptor, alpha polypeptide, antigen CD51) | 7953383 |
| HIVEP2 | 2.1 | human immunodeficiency virus type I enhancer binding protein 2 | 7905028 |
| TNFRSF9 | 2.1 | tumor necrosis factor receptor superfamily, member 9 | 7983650 |
| NR0B2 | 2.1 | nuclear receptor subfamily 0, group B, member 2 | 8122058 |
| DUSP4 | 2.1 | dual specificity phosphatase 4 | 7931810 |
| HRASLS3 | 2.1 | HRAS-like suppressor 3 | 7998510 |
| DKFZP564O0823 | 2.1 | DKFZP564O0823 protein | 8064175 |
| IFRD1 | 2.1 | interferon-related developmental regulator 1 | 8164129 |
| RNF103 | 2.1 | ring finger protein 103 | 8087447 |
| MAFG | 2.1 | v-maf musculoaponeurotic fibrosarcoma oncogene homolog G (avian) | 7933204 |
| PTP4A3 | 2.1 | protein tyrosine phosphatase type IVA, member 3 | 8175593 |
| RAB3B | 2.1 | RAB3B, member RAS oncogene family | 7989073 |
| DUSP10 | 2.1 | dual specificity phosphatase 10 | 7995825 |
| IRF1 | 2.1 | interferon regulatory factor 1 | 8107996 |
| SKIL | 2.1 | SKI-like oncogene | 8157890 |
| GABRG1 | 2.1 | gamma-aminobutyric acid (GABA) A receptor, gamma 1 | 8135990 |
| PQLC2 | 2.1 | PQ loop repeat containing 2 | 7926677 |
| DTNA | 2.1 | dystrobrevin, alpha /// dystrobrevin, alpha (DTNA), transcript variant 2/7/3 | 8047763 |
| SERPINE1 | 2.1 | serpin peptidase inhibitor, clade E (nexin, plasminogen activator inhibitor type 1), member 1 | 7973530 |
| HEXB | 2.1 | hexosaminidase B (beta polypeptide) | 8101774 |
| SEMA6A | 2.1 | sema domain, transmembrane domain (TM), and cytoplasmic domain, (semaphorin) 6A | 8113914 |
| CASZ1 | 2.1 | castor zinc finger 1 (CASZ1), transcript variant 1, mRNA | 8074063 |
| CYLD | 2.1 | cylindromatosis (turban tumor syndrome) (CYLD), transcript variant 1/2/3, mRNA | 7931168 |
| MAFG | 2.1 | v-maf musculoaponeurotic fibrosarcoma oncogene homolog G (avian) | 8008454 |
| OSGIN1 | 2.1 | oxidative stress induced growth inhibitor 1 | 8089112 |
| CASZ1 | 2.1 | castor zinc finger 1 (CASZ1), transcript variant 1, mRNA | 8117543 |
| CAV2 | 2.1 | caveolin 2 | 7916024 |
| ENC1 | 2.1 | ectodermal-neural cortex (with BTB-like domain) | 8117330 |
| IL18 | 2.0 | interleukin 18 (interferon-gamma-inducing factor), mRNA | 7938485 |
| SAMD4A | 2.0 | sterile alpha motif domain containing 4A | 7940717 |
| NPC2 | 2.0 | Niemann-Pick disease, type C2 | 7944722 |
| GPT2 | 2.0 | glutamic pyruvate transaminase (alanine aminotransferase) 2 | 8050350 |
| SEMA7A | 2.0 | semaphorin 7A, GPI membrane anchor (John Milton Hagen blood group) | 8053025 |
| DKFZp761B107 | 2.0 | hypothetical protein DKFZp761B107 | 8028955 |
| LONRF1 | 2.0 | LON peptidase N-terminal domain and ring finger 1 | 8174361 |
| SHC2 | 2.0 | SHC (Src homology 2 domain containing) transforming protein 2 (SHC2), mRNA | 7952223 |
| MFSD1 | 2.0 | major facilitator superfamily domain containing 1 | 8172670 |
| TES | 2.0 | testis derived transcript (3 LIM domains) | 7902290 |
| SNCA | 2.0 | synuclein, alpha (non A4 component of amyloid precursor) (SNCA), transcript variant NACP112, mRNA | 8052416 |
| KIAA0415 | 2.0 | KIAA0415, mRNA | 7920839 |
| ICA1 | 2.0 | islet cell autoantigen 1, 69kDa | 8046048 |
| PPIC | 2.0 | peptidylprolyl isomerase C (cyclophilin C) | 8008885 |
| POMT2 | 2.0 | protein-O-mannosyltransferase 2 | 7903144 |
| KLHL24 | 2.0 | kelch-like 24 (Drosophila) | 8089723 |
| SKAP2 | 2.0 | src kinase associated phosphoprotein 2 | 7944867 |
| EPCAM | 2.0 | epithelial cell adhesion molecule | 8060850 |
| HABP4 | 2.0 | hyaluronan binding protein 4 | 7988467 |
| SLC2A6 | 2.0 | solute carrier family 2 (facilitated glucose transporter), member 6 | 8124540 |
| OLFM3 | 2.0 | olfactomedin 3 | 7908917 |
| KRT19 | 2.0 | keratin 19 | 7945641 |
| SLC43A1 | 2.0 | solute carrier family 43, member 1 | 7899627 |
| MUC13 | 2.0 | mucin 13, cell surface associated | 8091071 |
| GPX7 | 2.0 | glutathione peroxidase 7 | 8123388 |
| FAM80B | 2.0 | family with sequence similarity 80, member B (FAM80B), mRNA | 8049961 |
| LOC202451 /// KIAA1244 | 2.0 | hypothetical protein LOC202451 /// KIAA1244 | 8169541 |
| TIMP4 | 2.0 | TIMP metallopeptidase inhibitor 4 | 8095214 |
| WARS | 2.0 | tryptophanyl-tRNA synthetase | 8077366 |
| SLC17A5 | 2.0 | solute carrier family 17 (anion/sugar transporter), member 5 | 8166072 |
| MANBA | 2.0 | mannosidase, beta A, lysosomal | 8046861 |
| SLC12A2 | 2.0 | solute carrier family 12 (sodium/potassium/chloride transporters), member 2 | 8129953 |
| NEDD9 | 2.0 | neural precursor cell expressed, developmentally down-regulated 9 | 7912145 |
| KLF7 | 2.0 | Kruppel-like factor 7 (ubiquitous) | 7914000 |
| PACS1 | 2.0 | phosphofurin acidic cluster sorting protein 1 | 8123364 |
| FZD5 | 2.0 | frizzled homolog 5 (Drosophila) | 7910377 |
| SYTL5 | 2.0 | synaptotagmin-like 5 | 8150076 |
| HEXA | 2.0 | hexosaminidase A (alpha polypeptide) | 8138337 |
| CLCN6 | 2.0 | chloride channel 6 transcript variant ClC-6c / transcript variant ClC-6b, ClC-6d, mRNA | 7948987 |
| MAPK13 | 2.0 | mitogen-activated protein kinase 13 | 8095751 |
| GNPDA1 | 2.0 | glucosamine-6-phosphate deaminase 1 | 8135514 |
| TP53INP2 | 2.0 | tumor protein p53 inducible nuclear protein 2 | 8053576 |
| RCAN2 | 2.0 | regulator of calcineurin 2 (RCAN2), mRNA | 8040430 |
| FLVCR2 | 2.0 | feline leukemia virus subgroup C cellular receptor family, member 2 | 8019796 |
| CCPG1 | 2.0 | cell cycle progression 1, transcript variant 2, mRNA; cell cycle progression 1, transcript variant 1, mRNA | 8148501 |
| NRP2 | 2.0 | neuropilin 2 mRNA | 7916112 |
| ADFP | 2.0 | adipose differentiation-related protein | 7924450 |
| RBCK1 | 2.0 | RanBP-type and C3HC4-type zinc finger containing 1 | 8114010 |
| CTNS | 1.9 | cystinosis, nephropathic | 8014755 |
| LHFPL2 | 1.9 | lipoma HMGIC fusion partner-like 2 | 8083876 |
| MAPKAPK3 | 1.9 | mitogen-activated protein kinase-activated protein kinase 3 | 8100097 |
| FSTL3 | 1.9 | follistatin-like 3 (secreted glycoprotein) | 7898556 |
| CASZ1 /// CASZ1 | 1.9 | castor zinc finger 1 /// castor zinc finger 1 (CASZ1), transcript variant 1, mRNA | 8020847 |
| RAP1GAP | 1.9 | RAP1 GTPase activating protein | 8098326 |
| RFPL4B | 1.9 | ret finger protein-like 4B | 8135069 |
| SLC2A8 | 1.9 | solute carrier family 2, (facilitated glucose transporter) member 8 | 8106252 |
| CEBPG | 1.9 | CCAAT/enhancer binding protein (C/EBP), gamma | 8124492 |
| EPHA5 | 1.9 | EPH receptor A5 | 8113666 |
| SLC30A1 | 1.9 | solute carrier family 30 (zinc transporter), member 1 | 7912343 |
| SAT2 | 1.9 | spermidine/spermine N1-acetyltransferase family member 2 | 8156923 |
| SLC1A5 | 1.9 | solute carrier family 1 (neutral amino acid transporter), member 5 | 7995552 |
| SMOX | 1.9 | spermine oxidase | 8019308 |
| FAM21A /// FAM21C | 1.9 | family with sequence similarity 21, member A /// family with sequence similarity 21, member C | 7997533 |
| DNAJC10 | 1.9 | DnaJ (Hsp40) homolog, subfamily C, member 10 | 7912347 |
| RND3 | 1.9 | Rho family GTPase 3 | 8020139 |
| GNA13 | 1.9 | guanine nucleotide binding protein (G protein), alpha 13 | 8135587 |
| MAG1 | 1.9 | lung cancer metastasis-associated protein | 7899392 |
| TMEM154 | 1.9 | transmembrane protein 154 | 8111474 |
| WDSUB1 | 1.9 | WD repeat, sterile alpha motif and U-box domain containing 1 | 8112615 |
| PLEKHH2 | 1.9 | pleckstrin homology domain containing, family H (with MyTH4 domain) member 2 | 8175121 |
| RBM24 | 1.9 | RNA binding motif protein 24 | 7951686 |
| MAFK | 1.9 | v-maf musculoaponeurotic fibrosarcoma oncogene homolog K (avian) | 8167625 |
| VAT1 | 1.9 | vesicle amine transport protein 1 homolog (T. californica) | 7974425 |
| C5orf26 | 1.9 | chromosome 5 open reading frame 26 | 7980146 |
| C20orf199 | 1.9 | chromosome 20 open reading frame 199 | 8117426 |
| DNAJB9 | 1.9 | DnaJ (Hsp40) homolog, subfamily B, member 9 | 7901338 |
| GBP2 | 1.9 | guanylate binding protein 2, interferon-inducible | 7995362 |
| G6PD /// G6PD | 1.9 | glucose-6-phosphate dehydrogenase /// glucose-6-phosphate dehydrogenase, transcript variant 2, mRNA | 7990345 |
| IRAK2 | 1.9 | interleukin-1 receptor-associated kinase 2 | 7984259 |
| ETS2 | 1.9 | v-ets erythroblastosis virus E26 oncogene homolog 2 (avian) | 8099670 |
| PDGFA | 1.9 | platelet-derived growth factor alpha polypeptide (PDGFA), transcript variant 2, mRNA | 8149399 |
| SPP1 | 1.9 | secreted phosphoprotein 1 (osteopontin, bone sialoprotein I, early T-lymphocyte activation 1), transcript variant 1, | 8032037 |
| SARS | 1.9 | seryl-tRNA synthetase | 8083656 |
| SEC11C | 1.9 | SEC11 homolog C (S. cerevisiae) | 8135576 |
| MBNL2 | 1.9 | muscleblind-like 2 (Drosophila) | 8101762 |
| FAM21A /// FAM21B /// FAM21C | 1.9 | family with sequence similarity 21, member A/B/C , mRNA | 8056102 |
| FAM21A /// FAM21B /// FAM21C | 1.9 | family with sequence similarity 21, member A/B/C mRNA | 8164194 |
| FLCN | 1.9 | folliculin | 8040465 |
| FAM21A /// FAM21B /// FAM21C | 1.9 | family with sequence similarity 21, member A/B/C , mRNA | 8131265 |
| VCAN | 1.9 | versican | 8138202 |
| SFT2D1 | 1.9 | SFT2 domain containing 1 | 8113726 |
| EDN1 | 1.9 | endothelin 1 | 7980358 |
| C1orf9 | 1.9 | chromosome 1 open reading frame 9 | 7999362 |
| EMP3 | 1.9 | epithelial membrane protein 3 | 7967030 |
| MST1 /// MSTP9 | 1.9 | macrophage stimulating 1 (hepatocyte growth factor-like) /// macrophage stimulating, pseudogene 9 | 8084219 |
| SLC6A9 | 1.9 | solute carrier family 6 (neurotransmitter transporter, glycine), member 9 mRNA | 8138689 |
| KIAA1946 | 1.9 | KIAA1946 | 8157381 |
| PROM1 | 1.9 | prominin 1 (PROM1), mRNA | 8098439 |
| MAP1LC3B | 1.9 | microtubule-associated protein 1 light chain 3 beta | 8062571 |
| THSD4 | 1.9 | thrombospondin, type I, domain containing 4 (THSD4), mRNA | 8156610 |
| CLGN | 1.9 | calmegin | 8164918 |
| MSTP9 /// MST1 | 1.9 | macrophage stimulating, pseudogene 9 /// macrophage stimulating 1 (hepatocyte growth factor-like) | 7918052 |
| DLAT | 1.9 | dihydrolipoamide S-acetyltransferase (E2 component of pyruvate dehydrogenase complex) | 8015349 |
| RAB3IL1 | 1.9 | RAB3A interacting protein (rabin3)-like 1 | 7948249 |
| AFF4 | 1.9 | AF4/FMR2 family, member 4 | 8090180 |
| SLC7A5 | 1.9 | solute carrier family 7 (cationic amino acid transporter, y+ system), member 5 | 7901460 |
| --- | 1.9 | --- | 7953765 |
| GBA | 1.9 | glucosidase, beta; acid (includes glucosylceramidase) | 8122279 |
| --- | 1.9 | --- | 8148070 |
| NFKB2 | 1.9 | nuclear factor of kappa light polypeptide gene enhancer in B-cells 2 (p49/p100) , mRNA | 8013671 |
| SPINK1 | 1.8 | serine peptidase inhibitor, Kazal type 1 | 7962559 |
| C9orf91 | 1.8 | chromosome 9 open reading frame 91 | 8085360 |
| TPP1 | 1.8 | tripeptidyl peptidase I | 7981290 |
| VPS13C | 1.8 | vacuolar protein sorting 13 homolog C (S. cerevisiae) (VPS13C), transcript variant 2B, mRNA | 8127549 |
| LOC284371 | 1.8 | hypothetical protein LOC284371 | 8102006 |
| UPP1 | 1.8 | uridine phosphorylase 1 | 8124531 |
| RBMS2 | 1.8 | RNA binding motif, single stranded interacting protein 2 | 8107769 |
| ASS1 | 1.8 | argininosuccinate synthetase 1 | 8123936 |
| SLC9A1 | 1.8 | solute carrier family 9 (sodium/hydrogen exchanger), member 1 (antiporter, Na+/H+, amiloride sensitive) | 8058477 |
| RCBTB1 | 1.8 | regulator of chromosome condensation (RCC1) and BTB (POZ) domain containing protein 1 | 7941537 |
| SH3BP2 | 1.8 | SH3-domain binding protein 2 | 8058498 |
| HEG1 | 1.8 | HEG homolog 1 (zebrafish) (HEG1), mRNA | 8094361 |
| DPP4 | 1.8 | dipeptidyl-peptidase 4 (CD26, adenosine deaminase complexing protein 2) | 8012043 |
| PINK1 | 1.8 | nuclear gene encoding mitochondrial protein, mRNA.Isoform 1 of Serine/threonine-protein kinase PINK1 | 8166747 |
| ZNF295 | 1.8 | zinc finger protein 295, transcript variant 3, mRNA /// zinc finger protein 295 , transcript variant 1, mRNA | 7990211 |
| DUSP16 | 1.8 | dual specificity phosphatase 16 | 8117594 |
| OBFC2A | 1.8 | oligonucleotide/oligosaccharide-binding fold containing 2A | 7897774 |
| GPR133 | 1.8 | G protein-coupled receptor 133 | 7941587 |
| CIDEC | 1.8 | cell death-inducing DFFA-like effector c | 8119016 |
| FBLIM1 | 1.8 | filamin binding LIM protein 1 | 8114787 |
| EDEM1 | 1.8 | ER degradation enhancer, mannosidase alpha-like 1 | 8062034 |
| FAM50A | 1.8 | family with sequence similarity 50, member A | 8126760 |
| KRTHB5 /// KRTHB5 | 1.8 | keratin, hair, basic, 5 /// keratin, hair, basic, 5 (KRTHB5), mRNA | 7975799 |
| ZNF165 | 1.8 | zinc finger protein 165 | 8124510 |
| C18orf58 | 1.8 | chromosome 18 open reading frame 58 | 7989037 |
| ZFYVE1 | 1.8 | zinc finger, FYVE domain containing 1 /// zinc finger, FYVE domain containing 1, transcript variant 2, mRNA | 8047738 |
| HPGD | 1.8 | hydroxyprostaglandin dehydrogenase 15-(NAD) | 8160297 |
| SLC22A15 | 1.8 | solute carrier family 22 (organic cation transporter), member 15 (SLC22A15), mRNA | 8106250 |
| BMPR1B | 1.8 | bone morphogenetic protein receptor, type IB | 8060353 |
| TMEM87B | 1.8 | transmembrane protein 87B | 7952179 |
| UBL3 | 1.8 | ubiquitin-like 3 | 8044961 |
| UIMC1 | 1.8 | ubiquitin interaction motif containing 1 | 8003824 |
|  | 1.8 | --- | 8079368 |
| TMEM56 | 1.8 | transmembrane protein 56 | 8112803 |
| AKR1C2 | 1.8 | aldo-keto reductase family 1, member C2 | 8080013 |
| APOL6 | 1.8 | apolipoprotein L, 6 | 8151123 |
| DACT2 | 1.8 | dapper, antagonist of beta-catenin, homolog 2 (Xenopus laevis) (DACT2), mRNA | 8117537 |
| ANKRD29 | 1.8 | ankyrin repeat domain 29 | 8151991 |
| SLC33A1 | 1.8 | solute carrier family 33 (acetyl-CoA transporter), member 1 | 7900508 |
| NAGK | 1.8 | N-acetylglucosamine kinase | 8145822 |
| DCDC2 | 1.8 | doublecortin domain containing 2 | 8023995 |
| ANKRD12 | 1.8 | ankyrin repeat domain 12 /// ankyrin repeat domain 12 (ANKRD12), transcript variant 2, mRNA | 7912316 |
| NPC1L1 | 1.8 | NPC1 (Niemann-Pick disease, type C1, gene)-like 1 (NPC1L1), transcript variant 2, mRNA | 8152463 |
| VPS18 | 1.8 | vacuolar protein sorting 18 homolog (S. cerevisiae) | 8003656 |
| C9orf30 | 1.8 | chromosome 9 open reading frame 30 | 7913385 |
| C6orf1 | 1.8 | chromosome 6 open reading frame 1 | 8094596 |
| ADRA2C | 1.8 | adrenergic, alpha-2C-, receptor | 7977507 |
| TMEFF1 | 1.8 | transmembrane protein with EGF-like and two follistatin-like domains 1 | 8121553 |
| DPP7 | 1.8 | dipeptidyl-peptidase 7 | 8158009 |
| CCNB1IP1 /// CCNB1IP1 | 1.8 | cyclin B1 interacting protein 1 /// cyclin B1 interacting protein 1 (CCNB1IP1), transcript variant 1, mRNA | 8117614 |
| FLJ35776 /// FLJ35776 | 1.8 | hypothetical protein LOC649446 /// hypothetical protein LOC649446 (FLJ35776), mRNA | 8027566 |
| DHRS3 | 1.8 | dehydrogenase/reductase (SDR family) member 3 | 8100578 |
| CHST3 | 1.8 | carbohydrate (chondroitin 6) sulfotransferase 3 | 7973054 |
| ZBTB38 | 1.8 | zinc finger and BTB domain containing 38 | 7924092 |
| ARMCX1 | 1.8 | armadillo repeat containing, X-linked 1 | 8012247 |
| SMAD7 | 1.8 | SMAD family member 7 | 8037835 |
| TMSL2 /// TMSL6 /// TMSB4X | 1.8 | thymosin-like 2 /// thymosin-like 6 /// thymosin, beta 4, X-linked | 8060745 |
| NUAK2 | 1.8 | NUAK family, SNF1-like kinase, 2 | 7927513 |
| GDEP | 1.8 | gene differentially expressed in prostate | 8046759 |
| --- | 1.8 | --- | 8131326 |
| --- | 1.8 | --- | 8055688 |
| LMCD1 | 1.8 | LIM and cysteine-rich domains 1 | 8017711 |
| SLC20A1 | 1.8 | solute carrier family 20 (phosphate transporter), member 1 | 8096116 |
| --- | 1.8 | --- | 8103226 |
| LSP1 | 1.8 | Lymphocyte specific protein 1 | 8056047 |
| NPAL2 | 1.8 | NIPA-like domain containing 2 | 7966299 |
| FAM57A | 1.8 | family with sequence similarity 57, member A | 8041644 |
| TMEM45B | 1.8 | transmembrane protein 45B | 8155596 |
| TAPBP | 1.8 | TAP binding protein (tapasin) | 8117045 |
| PCTK3 | 1.8 | PCTAIRE protein kinase 3, transcript variant 2, mRNA; PCTAIRE protein kinase 3 , transcript variant 3, mRNA | 8017599 |
| TMEM192 | 1.8 | transmembrane protein 192 /// hypothetical protein FLJ38482 (FLJ38482), mRNA | 8131091 |
| RNF185 | 1.8 | ring finger protein 185 | 8015759 |
| WHDC1 | 1.8 | WAS protein homology region 2 domain containing 1 (WHDC1), mRNA | 8107321 |
| KRTAP10-3 | 1.8 | keratin associated protein 10-3 /// keratin associated protein 10-3 (KRTAP10-3), mRNA | 8063337 |
| TC2N | 1.8 | tandem C2 domains, nuclear | 7994109 |
| ELF1 | 1.8 | E74-like factor 1 (ets domain transcription factor) | 8135480 |
| B3GALT1 | 1.8 | UDP-Gal:betaGlcNAc beta 1,3-galactosyltransferase, polypeptide 1 | 7917532 |
| PXK | 1.8 | PX domain containing serine/threonine kinase | 8176133 |
| DNASE2 | 1.8 | deoxyribonuclease II, lysosomal | 7995419 |
| TP53BP2 | 1.7 | tumor protein p53 binding protein, 2 (TP53BP2), transcript variant 1, mRNA | 8077786 |
| C12orf57 | 1.7 | chromosome 12 open reading frame 57 | 8128624 |
| SQSTM1 | 1.7 | sequestosome 1 | 8068593 |
| FAM83G | 1.7 | family with sequence similarity 83, member G /// family with sequence similarity 83, member G (FAM83G), mRNA | 8137670 |
| CASP8 | 1.7 | caspase 8 | 7937016 |
| HCFC2 | 1.7 | host cell factor C2 | 8096301 |
| FOSL2 | 1.7 | FOS-like antigen 2 | 7903619 |
| CLDN6 | 1.7 | claudin 6 | 8021453 |
| PTPRJ /// PTPRJ | 1.7 | protein tyrosine phosphatase, receptor type, J // transcript variant 2, | 7969677 |
| FLNA /// FLNA | 1.7 | filamin A, alpha (actin binding protein 280) /// filamin A, alpha (actin binding protein 280) (FLNA), mRNA | 8085311 |
| HDAC5 /// HDAC5 | 1.7 | histone deacetylase 5 /// histone deacetylase 5 (HDAC5), transcript variant 1, mRNA | 7927233 |
| PPP2R5B | 1.7 | protein phosphatase 2, regulatory subunit B', beta isoform | 8139919 |
| MAFG | 1.7 | v-maf musculoaponeurotic fibrosarcoma oncogene homolog G (avian) | 7927560 |
| WIPI1 | 1.7 | WD repeat domain, phosphoinositide interacting 1 | 8097541 |
| C1orf85 | 1.7 | chromosome 1 open reading frame 85 | 8013071 |
| DST | 1.7 | dystonin, transcript variant 1eB, mRNA // variant 1eA, mRNA | 7927323 |
| LOC285346 | 1.7 | hypothetical protein LOC285346 | 8106743 |
| IGF2R | 1.7 | insulin-like growth factor 2 receptor | 8045931 |
| FAM65A | 1.7 | family with sequence similarity 65, member A | 8130720 |
| OCLN | 1.7 | occludin | 8116921 |
| BAMBI | 1.7 | BMP and activin membrane-bound inhibitor homolog (Xenopus laevis) | 7907404 |
| ARL8B | 1.7 | ADP-ribosylation factor-like 8B | 8030007 |
| PDE5A | 1.7 | phosphodiesterase 5A, cGMP-specific | 8031867 |
| COQ10B | 1.7 | coenzyme Q10 homolog B (S. cerevisiae) | 8115234 |
| KCNT2 | 1.7 | potassium channel, subfamily T, member 2 | 7898357 |
| FVT1 | 1.7 | follicular lymphoma variant translocation 1 | 7915543 |
| STCH | 1.7 | stress 70 protein chaperone, microsome-associated, 60kDa | 8046895 |
| NHEDC2 | 1.7 | Na+/H+ exchanger domain containing 2 | 8099476 |
| C5orf28 | 1.7 | chromosome 5 open reading frame 28 | 7997740 |
| TNFRSF19 | 1.7 | tumor necrosis factor receptor superfamily, member 19 | 7984588 |
| SNORD49B | 1.7 | small nucleolar RNA, C/D box 49B | 8128865 |
| JUND | 1.7 | jun D proto-oncogene | 8102877 |
| MVP | 1.7 | major vault protein | 7912863 |
| FUT8 /// FUT8 | 1.7 | fucosyltransferase 8 (alpha (1,6) fucosyltransferase) , transcript variant 5, mRNA | 7943827 |
| AMOTL2 | 1.7 | angiomotin like 2 | 8109639 |
| NFE2L1 | 1.7 | nuclear factor (erythroid-derived 2)-like 1 | 7948643 |
| SLC17A2 | 1.7 | solute carrier family 17 (sodium phosphate), member 2 | 8114083 |
| RIOK3 | 1.7 | RIO kinase 3 (yeast) | 8127423 |
| OTUD7B /// OTUD7B | 1.7 | OTU domain containing 7B /// OTU domain containing 7B (OTUD7B), mRNA | 8003298 |
| SCAMP5 /// SCAMP5 | 1.7 | secretory carrier membrane protein 5 /// secretory carrier membrane protein 5 (SCAMP5), mRNA | 8102076 |
| HOXA11 | 1.7 | homeobox A11 | 7911335 |
| LITAF | 1.7 | lipopolysaccharide-induced TNF factor | 8134463 |
| MAFF | 1.7 | v-maf musculoaponeurotic fibrosarcoma oncogene homolog F (avian) | 7920697 |
| FOXO3 | 1.7 | forkhead box O3 | 8165694 |
| EXDL2 | 1.7 | exonuclease 3'-5' domain-like 2 | 7930074 |
| KRT23 | 1.7 | keratin 23 (histone deacetylase inducible) | 7988414 |
| METRNL | 1.7 | meteorin, glial cell differentiation regulator-like | 7918300 |
| TBX4 | 1.7 | T-box 4 | 8114964 |
| TMEM45A | 1.7 | transmembrane protein 45A | 8157463 |
| TUBE1 | 1.7 | tubulin, epsilon 1 | 7923189 |
| RELB /// RELB | 1.7 | v-rel reticuloendotheliosis viral oncogene homolog B, nuclear factor of kappa light polypeptide gene enhancer in B-cells 3 | 7946228 |
| TAPBP | 1.7 | TAP binding protein (tapasin) | 7989387 |
| SVIL | 1.7 | supervillin | 8034871 |
| MLXIPL | 1.7 | MLX interacting protein-like, transcript variant 2, mRNA /// MLX interacting protein-like, transcript variant 3, mRNA | 8099589 |
| NMI | 1.7 | N-myc (and STAT) interactor | 8160769 |
| OGFRL1 | 1.7 | opioid growth factor receptor-like 1 | 8038949 |
| SLC38A6 | 1.7 | solute carrier family 38, member 6 | 8132725 |
| BEST1 | 1.7 | bestrophin 1 | 7956261 |
| TNFAIP3 | 1.7 | tumor necrosis factor, alpha-induced protein 3 | 7940153 |
| SP100 /// SP100 | 1.7 | SP100 nuclear antigen /// SP100 nuclear antigen (SP100), transcript variant 1, mRNA | 8158671 |
| CASP4 /// CASP4 | 1.7 | caspase 4, apoptosis-related cysteine peptidase (CASP4), transcript variant gamma, mRNA | 7914021 |
| TRIB1 | 1.7 | tribbles homolog 1 (Drosophila) | 7971602 |
| PVR | 1.7 | poliovirus receptor | 8093624 |
| TMEM62 | 1.7 | transmembrane protein 62 | 8016504 |
| MT1JP /// MT1P3 | 1.7 | metallothionein 1J (pseudogene) /// metallothionein 1 pseudogene 3 | 7992594 |
| TAPBP | 1.7 | TAP binding protein (tapasin) | 7992967 |
| IRF9 | 1.7 | interferon regulatory factor 9 | 8090193 |
| JMY | 1.7 | junction-mediating and regulatory protein | 8056222 |
| OSTM1 | 1.7 | osteopetrosis associated transmembrane protein 1 | 7913249 |
| FLJ90709 | 1.7 | hypothetical protein FLJ90709 | 8070557 |
| SNX30 /// SNX30 | 1.7 | sorting nexin family member 30 /// sorting nexin family member 30 (SNX30), mRNA | 7961371 |
| TMEM185A | 1.7 | transmembrane protein 185A | 7999476 |
| HS1BP3 | 1.7 | HCLS1 binding protein 3 | 8047161 |
| ZNF643 | 1.7 | zinc finger protein 643 | 7970735 |
| USP54 | 1.7 | ubiquitin specific peptidase 54 | 7959893 |
| SLC25A36 | 1.7 | solute carrier family 25, member 36 | 8085244 |
| TMEM60 | 1.7 | transmembrane protein 60 | 7932498 |
| CSRP1 | 1.7 | cysteine and glycine-rich protein 1 | 7898263 |
| KIAA1539 | 1.7 | KIAA1539 | 8077458 |
| FLJ20160 | 1.7 | FLJ20160 protein | 8016016 |
| SLC1A5 | 1.7 | solute carrier family 1 | 8124423 |
| SERAC1 | 1.7 | serine active site containing 1 | 7960702 |
| LRRC49 | 1.7 | leucine rich repeat containing 49 | 8124380 |
| OSBPL10 | 1.7 | oxysterol binding protein-like 10 | 8170906 |
| MFSD11 | 1.7 | major facilitator superfamily domain containing 11 | 7959023 |
| FLJ30672 | 1.7 | hypothetical protein FLJ30672 | 8117630 |
| ABCG2 | 1.7 | ATP-binding cassette, sub-family G (WHITE), member 2 | 8022310 |
| B3GNT5 | 1.7 | UDP-GlcNAc:betaGal beta-1,3-N-acetylglucosaminyltransferase 5 | 7979984 |
| KLHL5 | 1.7 | kelch-like 5 (Drosophila) | 8124527 |
| WDR45 | 1.7 | WD repeat domain 45 | 8103769 |
| PTPN21 | 1.7 | protein tyrosine phosphatase, non-receptor type 21 | 7904226 |
| AGA | 1.7 | aspartylglucosaminidase | 8084679 |
| DTNBP1 | 1.7 | dystrobrevin binding protein 1 /// dystrobrevin binding protein 1 (DTNBP1), transcript variant 3, mRNA | 8018870 |
| RORA /// RORA | 1.7 | RAR-related orphan receptor A /// RAR-related orphan receptor A (RORA), transcript variant 4, mRNA | 8117395 |
| PIB5PA /// PIB5PA /// PIB5PA | 1.7 | phosphatidylinositol (4,5) bisphosphate 5-phosphatase, A (PIB5PA), transcript variant 1/2 mRNA | 8096511 |
| SH2B3 | 1.7 | SH2B adaptor protein 3 | 8044417 |
| TUFT1 | 1.7 | tuftelin 1 | 7970831 |
| SAA4 | 1.7 | serum amyloid A4, constitutive | 8029489 |
| MT2A | 1.7 | metallothionein 2A | 8078014 |
| DISP1 | 1.7 | dispatched homolog 1 (Drosophila) | 7999423 |
| SLC19A3 | 1.7 | solute carrier family 19, member 3 | 8179324 |
| SOX4 | 1.7 | SRY (sex determining region Y)-box 4 | 8178086 |
| DSCR3 | 1.7 | Down syndrome critical region gene 3 | 8165703 |
| KLHL29 | 1.7 | kelch-like 29 (Drosophila) | 7911343 |
| RHOC | 1.7 | ras homolog gene family, member C, transcript variant 2, / transcript variant 3 | 7993017 |
| C16orf62 | 1.7 | chromosome 16 open reading frame 62 | 8022640 |
| CTSA | 1.7 | cathepsin A | 8040223 |
| HAS2 | 1.7 | hyaluronan synthase 2 | 7903162 |
| ZNF852 | 1.7 | Zinc fingwe protein 852 | 7931832 |
| --- | 1.7 | --- | 8072710 |
| PNPLA8 | 1.7 | patatin-like phospholipase domain containing 8 | 8118314 |
| FLJ26056 /// DKFZP434B061 | 1.7 | hypothetical protein LOC375127 /// DKFZP434B061 protein | 7940160 |
| FHL3 | 1.7 | four and a half LIM domains 3 | 7992460 |
| INSR /// INSR | 1.7 | insulin receptor /// insulin receptor (INSR), transcript variant 2, mRNA | 7919749 |
| ADM | 1.7 | adrenomedullin | 8130859 |
| SLC6A11 | 1.7 | solute carrier family 6 (neurotransmitter transporter, GABA), member 11 | 8022559 |
| ZNF264 | 1.7 | zinc finger protein 264 | 8091637 |
| GCA | 1.7 | grancalcin, EF-hand calcium binding protein | 8042576 |
| NHSL1 | 1.7 | NHS-like 1 | 7915170 |
| TM4SF1 | 1.7 | transmembrane 4 L six family member 1 | 8124196 |
| PAK1 | 1.7 | p21/Cdc42/Rac1-activated kinase 1 (STE20 homolog, yeast) | 8020068 |
| CCL20 | 1.7 | chemokine (C-C motif) ligand 20 | 8075616 |
| KIAA0913 /// KIAA0913 | 1.7 | KIAA0913 /// KIAA0913 (KIAA0913), mRNA | 8104930 |
| TSPAN15 | 1.7 | tetraspanin 15 | 8049299 |
| GPR158 | 1.7 | G protein-coupled receptor 158 | 8139367 |
| SH3RF1 | 1.7 | SH3 domain containing ring finger 1 | 8008784 |
| --- | 1.7 | --- | 7982845 |
| PDE3A | 1.7 | phosphodiesterase 3A, cGMP-inhibited | 8124406 |
| PPAPDC2 | 1.7 | phosphatidic acid phosphatase type 2 domain containing 2 | 8156897 |
| ATP6V1C1 | 1.7 | ATPase, H+ transporting, lysosomal 42kDa, V1 subunit C1 | 8101862 |
| --- | 1.7 | --- | 8125818 |
| EFTUD1 | 1.7 | elongation factor Tu GTP binding domain containing 1 (EFTUD1), transcript variant 1, mRNA | 8157362 |
| FGL1 | 1.7 | fibrinogen-like 1 | 8093826 |
| ZNF319 | 1.7 | zinc finger protein 319 | 8156905 |
| STK39 /// STK39 | 1.7 | serine threonine kinase 39 (STE20/SPS1 homolog, yeast) (STK39), mRNA | 8100853 |
| GRINA | 1.6 | glutamate receptor, ionotropic, N-methyl D-asparate-associated protein 1 (glutamate binding) | 8165438 |
| CARS | 1.6 | cysteinyl-tRNA synthetase, transcript variant 1/2/ 3, mRNA | 7977497 |
| LSAMP | 1.6 | limbic system-associated membrane protein | 7944363 |
| TMCO3 | 1.6 | transmembrane and coiled-coil domains 3 | 8019954 |
| CREB3L1 /// CREB3L1 | 1.6 | cAMP responsive element binding protein 3-like 1 /// cAMP responsive element binding protein 3-like 1, | 7947915 |
| CXorf61 | 1.6 | chromosome X open reading frame 61 | 8112902 |
| BCAR1 | 1.6 | breast cancer anti-estrogen resistance 1 | 7912537 |
| ARL2 | 1.6 | ADP-ribosylation factor-like 2 | 8124484 |
| CLIP1 | 1.6 | CAP-GLY domain containing linker protein 1 | 7928291 |
| SELM /// SELM | 1.6 | selenoprotein M /// selenoprotein M (SELM), mRNA | 8083092 |
| AKNA | 1.6 | AT-hook transcription factor | 8168868 |
| MGC48628 | 1.6 | similar to KIAA1680 protein | 8147689 |
| ATP6V1D | 1.6 | ATPase, H+ transporting, lysosomal 34kDa, V1 subunit D | 8023220 |
| PRDM4 | 1.6 | PR domain containing 4 | 8067007 |
| CAV1 | 1.6 | caveolin 1, caveolae protein, 22kDa | 8179322 |
| ZNF622 | 1.6 | zinc finger protein 622 | 7923753 |
| WWC1 | 1.6 | WW and C2 domain containing 1 | 8012403 |
| --- | 1.6 | --- | 8096027 |
| SPAG9 | 1.6 | sperm associated antigen 9 | 8165709 |
| ISG15 | 1.6 | ISG15 ubiquitin-like modifier | 8095362 |
| LOC441728 | 1.6 | similar to Golgin subfamily A member 6 (Golgin linked to PML) (Golgin-like protein) | 8077490 |
| HERPUD1 /// HERPUD1 | 1.6 | homocysteine-inducible, endoplasmic reticulum stress-inducible, ubiquitin-like domain member 1 , variant 2, | 8044499 |
| SMAD9 | 1.6 | SMAD family member 9 | 8045287 |
| MTHFR | 1.6 | 5,10-methylenetetrahydrofolate reductase (NADPH) | 7968126 |
| ARFGEF2 | 1.6 | ADP-ribosylation factor guanine nucleotide-exchange factor 2 (brefeldin A-inhibited) | 7982889 |
| FOXRED2 /// FOXRED2 | 1.6 | FAD-dependent oxidoreductase domain containing 2 (FOXRED2), transcript variant 2, mRNA | 8097476 |
| GLIPR1 | 1.6 | GLI pathogenesis-related 1 (glioma) | 8151952 |
| SECTM1 | 1.6 | secreted and transmembrane 1 | 8003611 |
| PBEF1 | 1.6 | pre-B-cell colony enhancing factor 1 | 7945169 |
| ATP6V0A1 | 1.6 | ATPase, H+ transporting, lysosomal V0 subunit a1 | 8089013 |
| --- | 1.6 | --- | 8180166 |
| ORAI3 | 1.6 | ORAI calcium release-activated calcium modulator 3 | 7909104 |
| --- | 1.6 | --- | 8103524 |
| IFIT2 /// IFIT2 | 1.6 | interferon-induced protein with tetratricopeptide repeats 2 mRNA | 8117589 |
| VPS41 | 1.6 | vacuolar protein sorting 41 homolog (S. cerevisiae) | 8072454 |
| MICAL1 | 1.6 | microtubule associated monoxygenase, calponin and LIM domain containing 1 | 8053030 |
| CEACAM1 | 1.6 | carcinoembryonic antigen-related cell adhesion molecule 1 (biliary glycoprotein), transcript variant 2, mRNA | 7985482 |
| CCDC68 | 1.6 | coiled-coil domain containing 68 | 8070780 |
| ISG20 | 1.6 | interferon stimulated exonuclease gene 20kDa | 8118310 |
| ZBTB38 /// ZBTB38 | 1.6 | zinc finger and BTB domain containing 38 /// zinc finger and BTB domain containing 38 (ZBTB38), mRNA | 7980891 |
| MCOLN1 | 1.6 | mucolipin 1 | 7971197 |
| BACH2 | 1.6 | BTB and CNC homology 1, basic leucine zipper transcription factor 2 | 8150489 |
| SLC38A2 | 1.6 | solute carrier family 38, member 2 | 7902158 |
| CLSTN3 | 1.6 | calsyntenin 3 | 8046078 |
| DNAJC1 | 1.6 | DnaJ (Hsp40) homolog, subfamily C, member 1 | 8124537 |
| KIAA1984 /// C9orf86 | 1.6 | KIAA1984 /// chromosome 9 open reading frame 86 | 8080781 |
| SESTD1 | 1.6 | SEC14 and spectrin domains 1 | 8034565 |
| RAB7L1 | 1.6 | RAB7, member RAS oncogene family-like 1 | 7924526 |
| KLF10 /// KLF10 | 1.6 | Kruppel-like factor 10 /// Kruppel-like factor 10 (KLF10), transcript variant 2, mRNA | 7981383 |
| PCBP4 | 1.6 | poly(rC) binding protein 4 | 8023059 |
| ZDHHC13 | 1.6 | zinc finger, DHHC-type containing 13 , transcript variant 1, mRNA /variant 2, mRNA | 8124437 |
| GPR109B | 1.6 | G protein-coupled receptor 109B | 7953564 |
| SC5DL | 1.6 | sterol-C5-desaturase (ERG3 delta-5-desaturase homolog, S. cerevisiae)-like | 8110569 |
| DAAM1 | 1.6 | dishevelled associated activator of morphogenesis 1 | 8013312 |
| CMTM3 /// CMTM3 | 1.6 | CKLF-like MARVEL transmembrane domain containing 3 (CMTM3), transcript variant 5, mRNA | 8047419 |
| TACSTD1 | 1.6 | tumor-associated calcium signal transducer 1 | 8178043 |
| OLFML3 | 1.6 | olfactomedin-like 3 | 8118209 |
| PLXNC1 | 1.6 | plexin C1 | 7958158 |
| RND1 | 1.6 | Rho family GTPase 1 | 8041048 |
| ZNF460 | 1.6 | zinc finger protein 460 | 8179291 |
| PPP1R3B | 1.6 | protein phosphatase 1, regulatory (inhibitor) subunit 3B | 7919637 |
| MAN2A2 | 1.6 | mannosidase, alpha, class 2A, member 2 | 7998898 |
| C18orf30 | 1.6 | chromosome 18 open reading frame 30 | 7939839 |
| IL6R | 1.6 | interleukin 6 receptor | 8176026 |
| --- | 1.6 | --- | 8015914 |
| BRI3 | 1.6 | brain protein I3 | 7941087 |
| AARS | 1.6 | alanyl-tRNA synthetase | 8102643 |
| SLC26A11 | 1.6 | solute carrier family 26, member 11 | 8019798 |
| ZNF548 | 1.6 | zinc finger protein 548 | 8017850 |
| FHOD3 | 1.6 | formin homology 2 domain containing 3 | 7920971 |
| MARVELD2 /// MARVELD2 | 1.6 | MARVEL domain containing 2 (MARVELD2), transcript variant 2, mRNA | 8127234 |
| DHRS7 | 1.6 | dehydrogenase/reductase (SDR family) member 7 | 8004167 |
| MARVELD2 /// MARVELD2 | 1.6 | MARVEL domain containing 2 /// MARVEL domain containing 2 (MARVELD2), transcript variant 2, mRNA | 8095080 |
| IQCB1 | 1.6 | IQ motif containing B1 | 8043036 |
| CDH17 | 1.6 | cadherin 17, LI cadherin (liver-intestine) | 8086494 |
| TLE1 | 1.6 | transducin-like enhancer of split 1 (E(sp1) homolog, Drosophila) | 7923582 |
| SCN9A | 1.6 | sodium channel, voltage-gated, type IX, alpha subunit | 8123181 |
| ICAM1 | 1.6 | intercellular adhesion molecule 1 (CD54), human rhinovirus receptor | 7996571 |
| RAB27A | 1.6 | RAB27A, member RAS oncogene family | 8105908 |
| NPC1 | 1.6 | Niemann-Pick disease, type C1 | 7926875 |
| DAPK3 | 1.6 | death-associated protein kinase 3 | 8077450 |
| ZFAND2A | 1.6 | zinc finger, AN1-type domain 2A | 8124397 |
| DTX3 /// DTX3 | 1.6 | deltex 3 homolog (Drosophila) /// deltex 3 homolog (Drosophila) (DTX3), mRNA | 8102532 |
| TIPARP | 1.6 | TCDD-inducible poly(ADP-ribose) polymerase | 8047217 |
| TSPYL2 | 1.6 | TSPY-like 2 | 7923043 |
| WBP5 | 1.6 | WW domain binding protein 5 | 8023656 |
| PIM3 | 1.6 | pim-3 oncogene | 7901316 |
| --- | 1.6 | --- | 8069532 |
| FLRT3 | 1.6 | fibronectin leucine rich transmembrane protein 3 | 8102050 |
| LOC387869 | 1.6 | similar to microtubule-associated proteins 1A/1B light chain 3 | 8111952 |
| TMSL1 /// TMSL2 /// TMSL6 /// TMSL3 /// TMSB4X | 1.6 | thymosin-like 1 /// thymosin-like 2 /// thymosin-like 6 /// thymosin-like 3 /// thymosin, beta 4, X-linked | 7968015 |
| WBP2 | 1.6 | WW domain binding protein 2 | 8005200 |
| RAB11FIP4 | 1.6 | RAB11 family interacting protein 4 (class II) | 8035445 |
| CHKB /// CPT1B /// CPT1B | 1.6 | carnitine palmitoyltransferase 1B (CPT1B), | 7994659 |
| CALD1 /// CALD1 /// CALD1 | 1.6 | caldesmon 1 /// caldesmon 1, transcript variant 4, mRNA /// caldesmon 1, transcript variant 5, mRNA | 7975136 |
| ZXDB | 1.6 | zinc finger, X-linked, duplicated B | 7927876 |
| GNAZ | 1.6 | guanine nucleotide binding protein (G protein), alpha z polypeptide | 8090852 |
| C6orf48 | 1.6 | chromosome 6 open reading frame 48 | 8008087 |
| DENND4B | 1.6 | DENN/MADD domain containing 4B | 8124365 |
| CD97 | 1.6 | CD97 molecule | 8020508 |
| C6orf48 | 1.6 | chromosome 6 open reading frame 48 | 8120860 |
| ACVR1 | 1.6 | activin A receptor, type I | 7919699 |
| C18orf37 | 1.6 | chromosome 18 open reading frame 37 | 8134431 |
| ATP6V1H | 1.6 | ATPase, H+ transporting, lysosomal 50/57kDa, V1 subunit H | 7984932 |
| LONP1 | 1.6 | lon peptidase 1, mitochondrial | 8138765 |
| SH3GLB1 | 1.6 | SH3-domain GRB2-like endophilin B1 | 7920201 |
| ARSJ /// ARSJ | 1.6 | arylsulfatase family, member J /// arylsulfatase family, member J (ARSJ), mRNA | 7999468 |
| PBEF1 | 1.6 | pre-B-cell colony enhancing factor 1 | 8073007 |
| NR4A1 | 1.6 | nuclear receptor subfamily 4, group A, member 1 | 8066981 |
| CCDC93 | 1.6 | coiled-coil domain containing 93 | 8013307 |
| ENAH | 1.6 | enabled homolog (Drosophila) | 8087935 |
| TACC2 | 1.6 | transforming, acidic coiled-coil containing protein 2 | 7975311 |
| PARP12 | 1.6 | poly (ADP-ribose) polymerase family, member 12 | 7934154 |
| --- | 1.6 | --- | 8124391 |
| DCHS2 | 1.6 | dachsous 2 (Drosophila) | 8015133 |
| TMEM55B /// TMEM55B | 1.6 | transmembrane protein 55B /// transmembrane protein 55B (TMEM55B), transcript variant 1, mRNA | 8010897 |
| RFC1 | 1.6 | replication factor C | 7901967 |
| TGFB2 | 1.6 | transforming growth factor, beta 2 | 8117583 |
| FAM55C | 1.6 | family with sequence similarity 55, member C | 7919627 |
| BICC1 | 1.6 | bicaudal C homolog 1 (Drosophila) | 8139640 |
| SDPR | 1.6 | serum deprivation response (phosphatidylserine binding protein) | 8008982 |
| CXCL5 | 1.6 | chemokine (C-X-C motif) ligand 5 | 7937020 |
| C20orf70 | 1.6 | C20orf69 | 8081288 |
| CHIC2 | 1.6 | cysteine-rich hydrophobic domain 2 | 8128977 |
| TGFBI /// TGFBI | 1.6 | transforming growth factor, beta-induced, 68kDa /// transforming growth factor, beta-induced, 68kDa, mRNA | 8029580 |
| LOC728377 /// ARHGEF5 | 1.6 | similar to rho guanine nucleotide exchange factor 5 /// Rho guanine nucleotide exchange factor (GEF) 5 | 7933008 |
| TARS | 1.6 | threonyl-tRNA synthetase | 7905067 |
| C13orf15 | 1.6 | chromosome 13 open reading frame 15 | 8178977 |
| EHD4 | 1.6 | EH-domain containing 4 | 7971444 |
| GRAMD3 | 1.6 | GRAM domain containing 3 | 7932796 |
| FZD4 | 1.6 | frizzled homolog 4 (Drosophila) | 8147132 |
| FLJ26056 | 1.6 | FLJ26056 | 8140085 |
| TXNDC10 | 1.6 | thioredoxin domain containing 10 | 8055702 |
| GRAMD1A /// GRAMD1A | 1.6 | GRAM domain containing 1A /// GRAM domain containing 1A (GRAMD1A), mRNA | 7930137 |
| LOC392288 | 1.6 | similar to microtubule-associated proteins 1A/1B light chain 3 | 8006319 |
| BHLHB9 | 1.6 | basic helix-loop-helix domain containing, class B, 9 | 8120602 |
| PDE4D /// LOC653198 /// PDE4D | 1.6 | phosphodiesterase 4D, cAMP-specific (phosphodiesterase E3 dunce homolog, Drosophila) mRNA | 8024885 |
| SRXN1 | 1.6 | sulfiredoxin 1 homolog (S. cerevisiae) | 8051411 |
| KIAA1370 /// KIAA1370 | 1.6 | KIAA1370 /// KIAA1370 (KIAA1370), mRNA | 7974816 |
| PDLIM3 | 1.6 | PDZ and LIM domain 3 | 7940582 |
| LOC392871 /// LOC340274 | 1.6 | similar to argininosuccinate synthetase /// similar to argininosuccinate synthetase | 8122265 |
| MEF2D | 1.6 | myocyte enhancer factor 2D | 8048940 |
| ATP6V1E1 /// ATP6V1E1 /// ATP6V1E1 | 1.6 | ATPase, H+ transporting, lysosomal 31kDa, V1 subunit E1, transcript variant 2, mRNA, transcript variant 3, | 7951372 |
| TRAM1 | 1.6 | translocation associated membrane protein 1 | 8091099 |
| ADPRH | 1.6 | ADP-ribosylarginine hydrolase | 8080212 |
| PALLD | 1.6 | palladin, cytoskeletal associated protein | 8148304 |
| C14orf179 | 1.6 | chromosome 14 open reading frame 179 | 8029437 |
| ZNF364 | 1.6 | zinc finger protein 364 | 7983157 |
| --- | 1.6 | --- | 8062119 |
| TMEM2 | 1.6 | transmembrane protein 2 | 8125713 |
| LOC643837 | 1.6 | hypothetical protein LOC643837 | 7910164 |
| GGTL3 | 1.6 | gamma-glutamyltransferase-like 3 | 7973618 |
| LCOR | 1.6 | ligand dependent nuclear receptor corepressor | 8106516 |
| DTD1 | 1.6 | D-tyrosyl-tRNA deacylase 1 homolog (S. cerevisiae) | 8128669 |
| ABCB1 | 1.6 | ATP-binding cassette, sub-family B (MDR/TAP), member 1 | 7954436 |
| DCK | 1.6 | deoxycytidine kinase | 8112121 |
| PARP6 | 1.6 | poly (ADP-ribose) polymerase family, member 6 | 8157253 |
| SCARNA7 | 1.6 | small Cajal body-specific RNA 7 | 8175621 |
| ALDOC | 1.6 | aldolase C, fructose-bisphosphate | 8050594 |
| NPPA | 1.6 | natriuretic peptide precursor A | 7900438 |
| OSMR | 1.6 | oncostatin M receptor | 7958455 |
| GULP1 | 1.6 | GULP, engulfment adaptor PTB domain containing 1 | 8018305 |
| SIPA1L2 /// SIPA1L2 | 1.6 | signal-induced proliferation-associated 1 like 2 /// signal-induced proliferation-associated 1 like 2, mRNA | 8006317 |
| FLJ26056 /// DKFZP434B061 | 1.6 | hypothetical protein LOC375127 /// DKFZP434B061 protein | 8058106 |
| PLEKHM3 | 1.6 | pleckstrin homology domain | 7934411 |
| FLNB | 1.6 | filamin B, beta (actin binding protein 278) | 8083063 |
| PINK1 | 1.6 | PTEN induced putative kinase 1 | 8054354 |
| SYF2 | 1.6 | SYF2 homolog, RNA splicing factor (S. cerevisiae) | 8140500 |
| PCMTD2 | 1.6 | protein-L-isoaspartate (D-aspartate) O-methyltransferase domain containing 2 | 8026989 |
| SMPD1 /// SMPD1 | 1.6 | sphingomyelin phosphodiesterase 1, acid lysosomal (acid sphingomyelinase), transcript variant 1, mRNA | 7923378 |
| RHOB | 1.6 | ras homolog gene family, member B | 8098712 |
| Mar-03 | 1.6 | membrane-associated ring finger (C3HC4) 3 | 8160981 |
| SPNS1 | 1.6 | spinster homolog 1 (Drosophila) | 8080562 |
| GPRC5C /// GPRC5C | 1.6 | G protein-coupled receptor, family C, group 5, member C (GPRC5C), transcript variant 2, mRNA | 8047078 |
| GLIS3 /// GLIS3 | 1.6 | GLIS family zinc finger 3 /// GLIS family zinc finger 3 (GLIS3), transcript variant 1, mRNA | 8039164 |
| STARD4 | 1.6 | StAR-related lipid transfer (START) domain containing 4 | 8026696 |
| LOC728323 C20orf69 | 1.6 |  | 8029854 |
| ACSS3 | 1.6 | acyl-CoA synthetase short-chain family member 3 | 8130474 |
| TAF13 | 1.6 | TAF13 RNA polymerase II, TATA box binding protein (TBP)-associated factor, 18kDa | 7984569 |
| LOC728377 /// FLJ43692 | 1.6 | similar to rho guanine nucleotide exchange factor 5 /// ARHGEF5-like | 8085984 |
| SYT1 | 1.6 | synaptotagmin I | 8116012 |
| LPP | 1.6 | LIM domain containing preferred translocation partner in lipoma | 8010092 |
| C19orf54 | 1.6 | chromosome 19 open reading frame 54 | 8175442 |
| SWAP70 | 1.6 | SWAP-70 protein | 7960436 |
| KSR1 /// KSR1 | 1.6 | kinase suppressor of ras 1 /// kinase suppressor of ras 1 (KSR1), mRNA | 8101675 |
| RPP25 | 1.6 | ribonuclease P/MRP 25kDa subunit | 7975390 |
| JUB | 1.6 | jub, ajuba homolog (Xenopus laevis) | 8084206 |
| GRPEL2 | 1.6 | GrpE-like 2, mitochondrial (E. coli) | 8094625 |
| NPNT | 1.6 | nephronectin | 8172538 |
| SCRN1 | 1.5 | secernin 1 | 7980098 |
| C5orf32 | 1.5 | chromosome 5 open reading frame 32 | 7980616 |
| FLRT2 | 1.5 | fibronectin leucine rich transmembrane protein 2 | 8103834 |
| SLC12A4 | 1.5 | solute carrier family 12 (potassium/chloride transporters), member 4 | 8138289 |
| CHMP4C | 1.5 | chromatin modifying protein 4C | 7991837 |
| TTLL1 /// TTLL1 | 1.5 | tubulin tyrosine ligase-like family, member 1 (TTLL1), transcript variant 2, mRNA | 8124022 |
| GPR98 /// GPR98 | 1.5 | G protein-coupled receptor 98 /// G protein-coupled receptor 98 (GPR98), transcript variant 1, mRNA | 8077526 |
| NFKBIE | 1.5 | nuclear factor of kappa light polypeptide gene enhancer in B-cells inhibitor, epsilon | 7989365 |
| GLS | 1.5 | glutaminase | 8072436 |
| ZNF32 | 1.5 | zinc finger protein 32 | 7929882 |
| FYN | 1.5 | FYN oncogene related to SRC, FGR, YES | 7958749 |
| PRAF2 | 1.5 | PRA1 domain family, member 2 | 7905428 |
| SLC16A5 | 1.5 | solute carrier family 16, member 5 (monocarboxylic acid transporter 6) | 7946977 |
| SPATA20 | 1.5 | spermatogenesis associated 20 | 7966257 |
| LASS5 | 1.5 | LAG1 homolog, ceramide synthase 5 | 8095376 |
| DUSP3 | 1.5 | dual specificity phosphatase 3 (vaccinia virus phosphatase VH1-related) | 8007493 |
| ARMCX3 | 1.5 | armadillo repeat containing, X-linked 3 | 8124446 |
| SELI /// SELI | 1.5 | selenoprotein I /// selenoprotein I (SELI), mRNA | 7919055 |
| --- | 1.5 | --- | 7909954 |
| SRGAP1 | 1.5 | SLIT-ROBO Rho GTPase activating protein 1 | 8059538 |
| ADAM9 /// ADAM9 | 1.5 | ADAM metallopeptidase domain 9 | 8045499 |
| BCL2L2 | 1.5 | BCL2-like 2 | 8117580 |
| PEA15 | 1.5 | phosphoprotein enriched in astrocytes 15 | 8117165 |
| NUDT14 | 1.5 | nudix (nucleoside diphosphate linked moiety X)-type motif 14 | 8053447 |
| SLC16A13 | 1.5 | solute carrier family 16, member 13 (monocarboxylic acid transporter 13) | 8109333 |
| MFHAS1 | 1.5 | malignant fibrous histiocytoma amplified sequence 1 | 8070269 |
| MYC | 1.5 | v-myc myelocytomatosis viral oncogene homolog (avian) | 8105828 |
| CELSR3 | 1.5 | cadherin, EGF LAG seven-pass G-type receptor 3 (flamingo homolog, Drosophila) | 8040490 |
| CRIM1 | 1.5 | cysteine rich transmembrane BMP regulator 1 (chordin-like) | 7918593 |
| GNS | 1.5 | glucosamine (N-acetyl)-6-sulfatase (Sanfilippo disease IIID) | 8091554 |
| C1RL | 1.5 | complement component 1, r subcomponent-like | 7908161 |
| SNX8 | 1.5 | sorting nexin 8 | 8112994 |
| C14orf24 | 1.5 | chromosome 14 open reading frame 24 | 8033987 |
| CTSO | 1.5 | cathepsin O | 7993680 |
| SELK /// SELK | 1.5 | selenoprotein K /// selenoprotein K (SELK), mRNA | 8063078 |
| B2M | 1.5 | beta-2-microglobulin | 8117598 |
| --- | 1.5 | --- | 8152617 |
| IL1R1 | 1.5 | interleukin 1 receptor, type I | 8071817 |
| SNX12 | 1.5 | sorting nexin 12 | 8079187 |
| PPP2R5A | 1.5 | protein phosphatase 2, regulatory subunit B', alpha isoform | 8013521 |
| F3 | 1.5 | coagulation factor III (thromboplastin, tissue factor) | 8142307 |
| HLCS | 1.5 | holocarboxylase synthetase (biotin-(proprionyl-Coenzyme A-carboxylase (ATP-hydrolysing)) ligase) | 7916412 |
| DENND4A /// DENND4A | 1.5 | DENN/MADD domain containing 4A /// DENN/MADD domain containing 4A (DENND4A), mRNA | 8074330 |
| PHC1 | 1.5 | polyhomeotic homolog 1 (Drosophila) | 7915147 |
| TRIM47 | 1.5 | tripartite motif-containing 47 | 8033362 |
| TCEA1 | 1.5 | transcription elongation factor A (SII), 1 | 8035232 |
| PHC1 | 1.5 | polyhomeotic homolog 1 (Drosophila) | 8013989 |
| CTA-221G9.4 | 1.5 | KIAA1671 protein | 8032214 |
| IRS2 | 1.5 | insulin receptor substrate 2 | 7938390 |
| FASTKD1 | 1.5 | FAST kinase domains 1 | 8077817 |
| GAP43 | 1.5 | growth associated protein 43 | 8124521 |
| EIF2AK3 | 1.5 | eukaryotic translation initiation factor 2-alpha kinase 3 | 8121515 |
| MT1B | 1.5 | metallothionein 1B | 8132960 |
| SMCR8 | 1.5 | Smith-Magenis syndrome chromosome region, candidate 8 | 8113773 |
| NES | 1.5 | nestin | 8124448 |
| SEC16B /// LOC730102 /// SEC16B | 1.5 | SEC16 homolog B (S. cerevisiae) /// hypothetical protein LOC730102 // SEC16 homolog B (S. cerevisiae), | 8031690 |
| IBTK | 1.5 | inhibitor of Bruton agammaglobulinemia tyrosine kinase | 8046003 |
| LIMCH1 | 1.5 | LIM and calponin homology domains 1 | 8129888 |
| C4orf18 | 1.5 | chromosome 4 open reading frame 18 | 8091411 |
| NFIX /// NFIX | 1.5 | nuclear factor I/X (CCAAT-binding transcription factor) mRNA | 7950578 |
| PHLPPL | 1.5 | PH domain and leucine rich repeat protein phosphatase-like | 8048864 |
| ATL3 | 1.5 | atlastin GTPase 3 | 7985918 |
| MTMR6 | 1.5 | myotubularin related protein 6 | 8074878 |
| HINT3 | 1.5 | histidine triad nucleotide binding protein 3 | 7928411 |
| POLD4 | 1.5 | polymerase (DNA-directed), delta 4 | 7928046 |
| LOC494141 | 1.5 | similar to mitochondrial carrier triple repeat 1 | 7926715 |
| THBS3 | 1.5 | thrombospondin 3 | 8103630 |
| TNFRSF10A | 1.5 | tumor necrosis factor receptor superfamily, member 10a | 7947110 |
| KLHDC5 | 1.5 | kelch domain containing 5 | 7959100 |
| RUSC2 | 1.5 | RUN and SH3 domain containing 2 | 7898084 |
| CSTA | 1.5 | cystatin A (stefin A) | 8108078 |
| EXOC1 | 1.5 | exocyst complex component 1 | 8161026 |
| CXCL2 | 1.5 | chemokine (C-X-C motif) ligand 2 | 8177460 |
| NFXL1 | 1.5 | nuclear transcription factor, X-box binding-like 1 | 8145532 |
| CHFR | 1.5 | checkpoint with forkhead and ring finger domains | 7954293 |
| ACOT9 /// ACOT9 | 1.5 | acyl-CoA thioesterase 9 /// acyl-CoA thioesterase 9 (ACOT9), transcript variant 1, mRNA | 8154151 |
| SPON2 | 1.5 | spondin 2, extracellular matrix protein | 8155212 |
| EPC1 | 1.5 | enhancer of polycomb homolog 1 (Drosophila) | 8147724 |
| TCEA1 | 1.5 | transcription elongation factor A (SII), 1 | 8157582 |
| ZNF581 /// ZNF580 | 1.5 | zinc finger protein 581 /// zinc finger protein 580 | 8176865 |
| SEC61G | 1.5 | Sec61 gamma subunit | 8077185 |
| LCAT | 1.5 | lecithin-cholesterol acyltransferase | 7951865 |
| AAK1 | 1.5 | AP2 associated kinase 1 | 7990879 |
| SLC38A1 /// SLC38A1 | 1.5 | solute carrier family 38, member 1, transcript variant 1/2 | 8149521 |
| FLJ11151 /// FLJ11151 /// FLJ11151 | 1.5 | hypothetical protein FLJ11151 /// hypothetical protein FLJ11151, transcript variant 1, mRNA variant 2, | 8001651 |
| TNFAIP2 | 1.5 | tumor necrosis factor, alpha-induced protein 2 | 8056545 |
| SDC4 | 1.5 | syndecan 4 | 7981427 |
| GARS /// GARS | 1.5 | glycyl-tRNA synthetase /// glycyl-tRNA synthetase (GARS), mRNA | 8130578 |
| LOC162073 | 1.5 | hypothetical protein LOC162073 | 8148694 |
| SCD | 1.5 | stearoyl-CoA desaturase (delta-9-desaturase) | 7945803 |
| GABRA2 | 1.5 | gamma-aminobutyric acid (GABA) A receptor, alpha 2 | 8049083 |
| C1orf26 | 1.5 | chromosome 1 open reading frame 26 | 8089714 |
| ZYX | 1.5 | zyxin | 7970301 |
| PDCD4 /// PDCD4 | 1.5 | programmed cell death 4 (neoplastic transformation inhibitor) (PDCD4), transcript variant 2, mRNA | 8124524 |
| LOC284988 /// LOC730041 | 1.5 | hypothetical LOC284988 /// similar to ras-like protein TC10 | 8046488 |
| HINT2 | 1.5 | histidine triad nucleotide binding protein 2 | 7939642 |
| GLRX2 | 1.5 | glutaredoxin 2 | 8037732 |
| PLAGL1 /// PLAGL1 /// PLAGL1 /// PLAGL1 /// PLAGL1 | 1.5 | pleiomorphic adenoma gene-like 1 , transcript variant 4/6/8, | 8174648 |
| YPEL2 | 1.5 | yippee-like 2 (Drosophila) | 7983969 |
| GZF1 | 1.5 | GDNF-inducible zinc finger protein 1 | 8002854 |
| FLJ26056 /// DKFZP434B061 | 1.5 | hypothetical protein LOC375127 /// DKFZP434B061 protein | 7941104 |
| SLC6A12 | 1.5 | solute carrier family 6 (neurotransmitter transporter, betaine/GABA), member 12 | 8113664 |
| SETD7 | 1.5 | SET domain containing (lysine methyltransferase) 7 | 7967255 |
| PTPN3 | 1.5 | protein tyrosine phosphatase, non-receptor type 3 | 8104119 |
| M6PR | 1.5 | mannose-6-phosphate receptor (cation dependent) | 8075462 |
| CTSD | 1.5 | cathepsin D | 8163569 |
| IKBKB | 1.5 | inhibitor of kappa light polypeptide gene enhancer in B-cells, kinase beta | 8079237 |
| SCYL1BP1 | 1.5 | SCY1-like 1 binding protein 1 | 8085358 |
| MPP1 | 1.5 | membrane protein, palmitoylated 1, 55kDa | 8096425 |
| SERINC1 | 1.5 | serine incorporator 1 | 7979698 |
| MGAT4B | 1.5 | mannosyl (alpha-1,3-)-glycoprotein beta-1,4-N-acetylglucosaminyltransferase, isozyme B | 7947189 |
| CIRBP | 1.5 | cold inducible RNA binding protein | 7966072 |
| KIAA1754 | 1.5 | KIAA1754 | 7907788 |
| ENO3 | 1.5 | enolase 3 (beta, muscle) | 8135594 |
| RNU5E | -3.8 | RNU5E | 7951163 |
| HP /// HP | -3.7 | haptoglobin /// haptoglobin (HP), mRNA | 8111129 |
| --- | -3.6 | --- | 8109773 |
| --- | -3.5 | --- | 8142431 |
| --- | -3.4 | --- | 8016745 |
| --- | -3.1 | --- | 7896817 |
| --- | -3.0 | --- | 8117339 |
| --- | -2.9 | --- | 7981059 |
| --- | -2.9 | --- | 7924884 |
| HPR /// HPR | -2.9 | haptoglobin-related protein /// haptoglobin-related protein (HPR), mRNA | 7985034 |
| HIST1H2BB | -2.9 | histone cluster 1, H2bb | 7995895 |
| RNU5D | -2.8 | RNA, U5D small nuclear | 8027402 |
| SPTLC3 | -2.8 | serine palmitoyltransferase, long chain base subunit 3 | 8128327 |
| LOC253012 | -2.8 | hypothetical protein LOC253012 | 7971015 |
| --- | -2.8 | --- | 7912496 |
| CD38 | -2.7 | CD38 molecule | 8063242 |
| METTL7A | -2.6 | methyltransferase like 7A | 8075785 |
| --- | -2.6 | --- | 7957260 |
| MYCN | -2.5 | v-myc myelocytomatosis viral related oncogene, neuroblastoma derived (avian) | 8019486 |
| MAP2K6 | -2.5 | mitogen-activated protein kinase kinase 6 | 8103728 |
| --- | -2.5 | --- | 7933084 |
| --- | -2.5 | --- | 8007228 |
| --- | -2.4 | --- | 8117653 |
| --- | -2.4 | --- | 8061746 |
| KIF20A | -2.4 | kinesin family member 20A | 8165684 |
| MOSC1 | -2.4 | MOCO sulphurase C-terminal domain containing 1 | 8000632 |
| HIST1H4D | -2.4 | histone cluster 1, H4d | 8168115 |
| ATP10D | -2.4 | ATPase, class V, type 10D | 7994981 |
| FOXG1 | -2.4 | forkhead box G1 | 8100521 |
| --- | -2.3 | --- | 7896748 |
| --- | -2.3 | --- | 7929047 |
| PRKAR2B | -2.3 | protein kinase, cAMP-dependent, regulatory, type II, beta | 8139165 |
| TSPAN18 /// TSPAN18 | -2.3 | tetraspanin 18 /// tetraspanin 18 (TSPAN18), transcript variant 1, mRNA | 8128737 |
| HIST1H1D | -2.3 | histone cluster 1, H1d | 8080960 |
| --- | -2.3 | --- | 8037205 |
| --- | -2.3 | --- | 8023401 |
| SCARNA10 | -2.3 | Homo sapiens small Cajal body-specific RNA 10 (SCARNA10), non-coding RNA. | 7985777 |
| SLC27A2 | -2.3 | solute carrier family 27 (fatty acid transporter), member 2 | 8144488 |
| ARG1 | -2.3 | arginase, liver | 8155081 |
| SLC2A4RG | -2.3 | SLC2A4 regulator | 8083090 |
| RNU4B1 | -2.3 | RNA, U4B1 small nuclear | 8025183 |
| PRTG /// PRTG | -2.2 | protogenin homolog (Gallus gallus) /// protogenin homolog (Gallus gallus) (PRTG), mRNA | 8128247 |
| --- | -2.2 | --- | 7900167 |
| PBX3 | -2.2 | pre-B-cell leukemia homeobox 3 | 8113214 |
| ACADSB | -2.2 | acyl-Coenzyme A dehydrogenase, short/branched chain | 7962537 |
| HIST1H2AH | -2.2 | histone cluster 1, H2ah | 7953626 |
| HIST1H3A | -2.2 | histone cluster 1, H3a | 7932512 |
| --- | -2.2 | --- | 8159415 |
| NAT8 /// NAT8B | -2.2 | N-acetyltransferase 8 /// N-acetyltransferase 8B (gene/pseudogene) (NAT8B), mRNA | 8057394 |
| --- | -2.2 | --- | 8058855 |
| DGKK /// DGKK | -2.2 | diacylglycerol kinase, kappa /// diacylglycerol kinase, kappa (DGKK), mRNA | 7923812 |
| --- | -2.2 | --- | 8171392 |
| FAM130A2 | -2.2 | family with sequence similarity 130, member A2 | 8014974 |
| --- | -2.2 | --- | 8130374 |
| SIAE | -2.2 | sialic acid acetylesterase | 8152215 |
| HIST1H2AM | -2.1 | histone cluster 1, H2am | 7934278 |
| RBP2 | -2.1 | retinol binding protein 2, cellular | 8054580 |
| --- | -2.1 | --- | 7901969 |
| CCR6 | -2.1 | chemokine (C-C motif) receptor 6 | 8122194 |
| --- | -2.1 | --- | 7947245 |
| TMEM195 | -2.1 | transmembrane protein 195 | 8087806 |
| VSNL1 | -2.1 | visinin-like 1 | 7938816 |
| SNORA21 | -2.1 | small nucleolar RNA, H/ACA box 21 | 8074958 |
| --- | -2.1 | --- | 7967322 |
| HIST1H2BK | -2.1 | Histone cluster 1 | 7944656 |
| RP11-35N6.1 | -2.1 | plasticity related gene 3 | 7917912 |
| --- | -2.1 | --- | 7974697 |
| SCARNA1 | -2.1 | small Cajal body-specific RNA 1 | 7979307 |
| AGXT2 | -2.1 | alanine-glyoxylate aminotransferase 2 | 7996318 |
| IGSF1 | -2.0 | immunoglobulin superfamily, member 1 | 8159786 |
| CCNB3 | -2.0 | cyclin B3 | 8041853 |
| HIST1H2BH | -2.0 | histone cluster 1, H2bh | 8147049 |
| --- | -2.0 | --- | 7904158 |
| RNU5B-1 | -2.0 | --- | 7957570 |
| CD302 | -2.0 | CD302 molecule | 8149625 |
| --- | -2.0 | --- | 7904463 |
| --- | -2.0 | --- | 7962884 |
| --- | -2.0 | --- | 7996563 |
| RNU4-1 | -2.0 | RNAu4-1 | 8171879 |
| ZNF618 | -2.0 | zinc finger protein 618 | 7948420 |
| FAM83D /// FAM83D | -2.0 | family with sequence similarity 83, member D /// family with sequence similarity 83, member D, mRNA | 7915590 |
| COL14A1 | -2.0 | collagen, type XIV, alpha 1 (undulin) | 8031714 |
| SPAG5 | -2.0 | sperm associated antigen 5 | 8149264 |
| SLC38A4 | -2.0 | solute carrier family 38, member 4 | 7945950 |
| HIST1H3I | -2.0 | histone cluster 1, H3i | 7986132 |
| GBA3 /// GBA3 | -2.0 | glucosidase, beta, acid 3 (cytosolic) /// glucosidase, beta, acid 3 (cytosolic) (GBA3), mRNA | 8022295 |
| ASGR1 | -2.0 | asialoglycoprotein receptor 1 | 7905789 |
| HIST1H2BM | -2.0 | histone cluster 1, H2bm | 8170390 |
| CNIH2 | -2.0 | cornichon homolog 2 (Drosophila) | 8019357 |
| HIST1H2BL | -2.0 | histone cluster 1, H2bl | 7969286 |
| --- | -2.0 | --- | 8134454 |
| H2AFX | -2.0 | H2A histone family, member X | 8117389 |
| RNU4ATAC | -2.0 | RNU4ATAC | 8002347 |
| --- | -1.9 | --- | 8010405 |
| PTTG3 | -1.9 | pituitary tumor-transforming 3 | 8031737 |
| HIST1H4I | -1.9 | histone cluster 1, H4i | 7919747 |
| --- | -1.9 | --- | 8020973 |
| --- | -1.9 | --- | 8177498 |
| --- | -1.9 | --- | 7979473 |
| --- | -1.9 | --- | 8105899 |
| SERPINF2 | -1.9 | serpin peptidase inhibitor, clade F (alpha-2 antiplasmin, pigment epithelium derived factor), member 2 | 8089954 |
| PTTG2 | -1.9 | pituitary tumor-transforming 2 | 8151795 |
| RPPH1 | -1.9 | ribonuclease P RNA component H1 | 8081941 |
| HIST1H2BO | -1.9 | histone cluster 1, H2bo | 8161919 |
| --- | -1.9 | --- | 8056491 |
| SLC29A4 /// SLC29A4 | -1.9 | solute carrier family 29 (nucleoside transporters), member 4 (SLC29A4), transcript variant 1, mRNA | 8170235 |
| --- | -1.9 | --- | 8058837 |
| --- | -1.9 | --- | 8025601 |
| PECAM1 /// PECAM1 | -1.9 | platelet/endothelial cell adhesion molecule (CD31 antigen) | 7989023 |
| PLK1 | -1.9 | polo-like kinase 1 (Drosophila) | 8067167 |
| --- | -1.9 | --- | 8022531 |
| --- | -1.9 | --- | 7961798 |
| CLRN3 | -1.9 | clarin 3 | 8039491 |
| ATP2B2 | -1.9 | ATPase, Ca++ transporting, plasma membrane 2 | 7993146 |
| --- | -1.9 | --- | 7968796 |
| --- | -1.9 | --- | 8032718 |
| --- | -1.9 | --- | 8117535 |
| --- | -1.9 | --- | 8137709 |
| ANXA6 /// ANXA6 /// ANXA6 | -1.9 | annexin A6 /// annexin A6, transcript variant 1, mRNA /// annexin A6, transcript variant 2, mRNA | 7956539 |
| --- | -1.9 | --- | 8083569 |
| PTTG1 | -1.9 | pituitary tumor-transforming 1 | 8167763 |
| --- | -1.9 | --- | 8169022 |
| CENPE | -1.9 | centromere protein E, 312kDa | 8073960 |
| NPTX2 | -1.9 | neuronal pentraxin II | 8121136 |
| GATM | -1.9 | glycine amidinotransferase (L-arginine:glycine amidinotransferase) | 8097064 |
| PSRC1 | -1.8 | proline/serine-rich coiled-coil 1 | 8124385 |
| KIF14 | -1.8 | kinesin family member 14 | 8065071 |
| --- | -1.8 | --- | 7964983 |
| --- | -1.8 | --- | 8158240 |
| --- | -1.8 | --- | 8018482 |
| FAM111A | -1.8 | family with sequence similarity 111, member A | 8006298 |
| --- | -1.8 | --- | 8177118 |
| CCNF | -1.8 | cyclin F | 7961887 |
| VASN | -1.8 | vasorin | 8077123 |
| --- | -1.8 | --- | 7981233 |
| --- | -1.8 | --- | 8136347 |
| C10orf140 | -1.8 | chromosome 10 open reading frame 140 | 8167942 |
| --- | -1.8 | --- | 8071671 |
| HIST1H2BG | -1.8 | histone cluster 1, H2bg | 8179326 |
| CDCA3 | -1.8 | cell division cycle associated 3 | 7920354 |
| HIST1H1A | -1.8 | histone cluster 1, H1a | 8066198 |
| HIST1H1B | -1.8 | histone cluster 1, H1b | 8026300 |
| KNG1 /// KNG1 | -1.8 | kininogen 1 /// kininogen 1 (KNG1), transcript variant 1, mRNA | 8178090 |
| --- | -1.8 | --- | 8105603 |
| HIST1H2BF | -1.8 | histone cluster 1, H2bf | 8056005 |
| BCAM /// BCAM | -1.8 | basal cell adhesion molecule /// basal cell adhesion molecule, transcript variant 2, | 8022902 |
| SLC6A6 | -1.8 | solute carrier family 6 (neurotransmitter transporter, taurine), member 6 | 8150797 |
| SOCS1 | -1.8 | suppressor of cytokine signaling 1 | 8033002 |
| HSPA1B /// HSPA1A | -1.8 | heat shock 70kDa protein 1B /// heat shock 70kDa protein 1A | 7902771 |
| HSPA1B /// HSPA1A | -1.8 | heat shock 70kDa protein 1B /// heat shock 70kDa protein 1A | 8102440 |
| --- | -1.8 | --- | 8142120 |
| DHFR /// DHFR | -1.8 | dihydrofolate reductase /// dihydrofolate reductase (DHFR), mRNA | 7955589 |
| RRM2 | -1.8 | ribonucleotide reductase M2 polypeptide | 8054804 |
| HSPA1B /// HSPA1A | -1.8 | heat shock 70kDa protein 1B /// heat shock 70kDa protein 1A | 7924619 |
| DTX4 /// DTX4 | -1.8 | deltex 4 homolog (Drosophila) /// deltex 4 homolog (Drosophila) (DTX4), mRNA | 7983393 |
| NPW /// NPW | -1.8 | neuropeptide W /// neuropeptide W (NPW), mRNA | 7931031 |
| --- | -1.8 | --- | 8047223 |
| MYCBP | -1.8 | c-myc binding protein | 8143327 |
| SYN3 /// SYN3 | -1.8 | synapsin III /// synapsin III (SYN3), transcript variant IIIc, mRNA | 8165680 |
| SLC1A3 | -1.8 | solute carrier family 1 (glial high affinity glutamate transporter), member 3 | 7905127 |
| SCARNA6 | -1.8 | small Cajal body-specific RNA 6 | 8103260 |
| PRR11 | -1.8 | proline rich 11 | 7977584 |
| HIST1H2BC | -1.8 | histone cluster 1, H2bc | 8055952 |
| ADH6 | -1.8 | alcohol dehydrogenase 6 (class V) | 8165672 |
| ZNF618 /// ZNF618 | -1.8 | zinc finger protein 618 /// zinc finger protein 618 (ZNF618), mRNA | 7909789 |
| GC | -1.8 | group-specific component (vitamin D binding protein) | 7960728 |
| --- | -1.8 | --- | 7919761 |
| --- | -1.8 | --- | 8081375 |
| DHFR /// DHFR | -1.8 | dihydrofolate reductase /// dihydrofolate reductase (DHFR), mRNA | 7956009 |
| HIST1H2BJ | -1.8 | histone cluster 1, H2bj | 7927681 |
| --- | -1.8 | --- | 8053654 |
| HSPA1A | -1.8 | heat shock 70kDa protein 1A | 8057797 |
| AURKB | -1.8 | aurora kinase B | 8100977 |
| NUSAP1 /// NUSAP1 | -1.8 | nucleolar and spindle associated protein 1, transcript variant 1/2, mRNA; | 8059277 |
| --- | -1.8 | --- | 7927095 |
| LOC648124 | -1.8 | similar to Vitamin K-dependent protein S precursor | 7994826 |
| HIST1H3H | -1.8 | histone cluster 1, H3h | 8100382 |
| NAT8B | -1.8 | N-acetyltransferase 8B (gene/pseudogene) | 8151942 |
| HSPA1A | -1.8 | heat shock 70kDa protein 1A | 7982927 |
| --- | -1.8 | --- | 8108217 |
| TCTEX1D1 | -1.8 | Tctex1 domain containing 1 | 8117608 |
| HIST1H3J | -1.8 | histone cluster 1, H3j | 7957604 |
| --- | -1.7 | --- | 8068898 |
| --- | -1.7 | --- | 8136987 |
| HIST1H3F | -1.7 | histone cluster 1, H3f | 7961483 |
| APOM | -1.7 | apolipoprotein M | 8104760 |
| APOM | -1.7 | apolipoprotein M | 8011747 |
| APOM | -1.7 | apolipoprotein M | 7968789 |
| HIST2H2BE | -1.7 | histone cluster 2, H2be | 7987772 |
| CCNA2 | -1.7 | cyclin A2 | 8107673 |
| FAM64A | -1.7 | family with sequence similarity 64, member A | 7950885 |
| PDGFRA | -1.7 | platelet-derived growth factor receptor, alpha polypeptide | 7980304 |
| LOC1720 | -1.7 | dihydrofolate reductase pseudogene | 8071375 |
| LOC100132728 | -1.7 | similar to cpn10 protein | 8023735 |
| HIST1H1C | -1.7 | histone cluster 1, H1c | 8027701 |
| SLC5A9 | -1.7 | solute carrier family 5 (sodium/glucose cotransporter), member 9 | 8012958 |
| CXXC6 | -1.7 | CXXC finger 6 | 8160317 |
| BCKDHB | -1.7 | branched chain keto acid dehydrogenase E1, beta polypeptide (maple syrup urine disease) | 8156194 |
| --- | -1.7 | --- | 8006606 |
| SPRR2B /// SPRR2F | -1.7 | small proline-rich protein 2B /// small proline-rich protein 2F | 8016213 |
| --- | -1.7 | --- | 8168984 |
| NT5DC2 | -1.7 | 5'-nucleotidase domain containing 2 | 8117600 |
| --- | -1.7 | --- | 8112220 |
| HIST1H2AB | -1.7 | histone cluster 1, H2ab | 8064375 |
| --- | -1.7 | --- | 8134834 |
| HIST1H3H /// HIST1H2AI | -1.7 | histone cluster 1, H3h /// histone cluster 1, H2ai | 7988970 |
| HIST2H4A /// HIST2H4B | -1.7 | histone cluster 2, H4a /// histone cluster 2, H4b | 8104022 |
| DDC /// DDC | -1.7 | dopa decarboxylase (aromatic L-amino acid decarboxylase) (DDC), transcript variant 1, mRNA | 8138487 |
| MKI67 | -1.7 | antigen identified by monoclonal antibody Ki-67 | 7965650 |
| --- | -1.7 | --- | 7921014 |
| HIST2H4A /// HIST2H4B | -1.7 | histone cluster 2, H4a /// histone cluster 2, H4b | 8141052 |
| CPB2 | -1.7 | carboxypeptidase B2 (plasma) | 8074251 |
| CA2 | -1.7 | carbonic anhydrase II | 8151281 |
| --- | -1.7 | --- | 8081880 |
| --- | -1.7 | --- | 8098263 |
| TNFAIP8L1 | -1.7 | tumor necrosis factor, alpha-induced protein 8-like 1 | 8066292 |
| --- | -1.7 | --- | 8052269 |
| LOC729687 | -1.7 | Like Nonhistone chromosomal protein HMG-17 | 7919570 |
| GLYCTK | -1.7 | glycerate kinase | 7975851 |
| CABC1 | -1.7 | chaperone, ABC1 activity of bc1 complex homolog (S. pombe) | 8023855 |
| LRMP | -1.7 | lymphoid-restricted membrane protein | 7904830 |
| UNG | -1.7 | uracil-DNA glycosylase | 7960340 |
| HN1 /// HN1 /// HN1 | -1.7 | hematological and neurological expressed 1 (HN1), transcript variant 1/3, mRNAmRNA | 7910636 |
| --- | -1.7 | --- | 7970842 |
| --- | -1.7 | --- | 8161701 |
| --- | -1.7 | --- | 7896759 |
| --- | -1.7 | --- | 8065798 |
| --- | -1.7 | --- | 7929596 |
| IL17RB | -1.7 | interleukin 17 receptor B | 8061211 |
| --- | -1.7 | --- | 8140782 |
| --- | -1.7 | --- | 8095574 |
| MXD3 | -1.7 | MAX dimerization protein 3 | 7940349 |
| FLJ44874 | -1.7 | FLJ44874 protein | 7990165 |
| SMOC1 | -1.7 | SPARC related modular calcium binding 1 | 8091778 |
| ALDH6A1 | -1.7 | aldehyde dehydrogenase 6 family, member A1 | 8013660 |
| ETV1 /// ETV1 | -1.7 | ets variant gene 1 /// ets variant gene 1 (ETV1), mRNA | 8048141 |
| NME4 | -1.7 | non-metastatic cells 4, protein expressed in | 7912515 |
| --- | -1.7 | --- | 8092523 |
| SEMA4G | -1.7 | sema domain, immunoglobulin domain (Ig), transmembrane domain (TM) and short cytoplasmic domain, | 8105040 |
| --- | -1.7 | --- | 7952830 |
| ARL4D | -1.7 | ADP-ribosylation factor-like 4D | 8070328 |
| --- | -1.7 | --- | 8046906 |
| HMGCS2 | -1.7 | 3-hydroxy-3-methylglutaryl-Coenzyme A synthase 2 (mitochondrial) | 7925062 |
| HNMT | -1.7 | histamine N-methyltransferase | 8071161 |
| HIST1H2AI | -1.7 | histone cluster 1, H2ai | 8058509 |
| --- | -1.7 | --- | 7971905 |
| GPX3 /// GPX3 | -1.7 | glutathione peroxidase 3 (plasma) /// glutathione peroxidase 3 (plasma) (GPX3), mRNA | 8080714 |
| CCNB1 | -1.7 | cyclin B1 | 7913252 |
| --- | -1.7 | --- | 7951038 |
| C1orf21 | -1.7 | chromosome 1 open reading frame 21 | 8149955 |
| --- | -1.7 | --- | 7948167 |
| ICAM3 | -1.7 | intercellular adhesion molecule 3 | 7982757 |
| HIST1H4K /// HIST1H4J | -1.7 | histone cluster 1, H4k /// histone cluster 1, H4j | 7913814 |
| --- | -1.7 | --- | 8000945 |
| FAM151A | -1.7 | family with sequence similarity 151, member A | 8109484 |
| --- | -1.7 | --- | 7924549 |
| SLC6A4 | -1.7 | solute carrier family 6 (neurotransmitter transporter, serotonin), member 4 | 7938100 |
| GAMT | -1.7 | guanidinoacetate N-methyltransferase | 8040473 |
| HIST1H4K /// HIST1H4J | -1.7 | histone cluster 1, H4k /// histone cluster 1, H4j | 8113790 |
| SLC16A10 | -1.7 | solute carrier family 16, member 10 (aromatic amino acid transporter) | 8124416 |
| --- | -1.7 | --- | 7896929 |
| ALDH7A1 | -1.7 | aldehyde dehydrogenase 7 family, member A1 | 7965606 |
| HIST1H4H | -1.7 | histone cluster 1, H4h | 7994518 |
| --- | -1.7 | --- | 8010260 |
| --- | -1.7 | --- | 8060286 |
| E2F8 | -1.7 | E2F transcription factor 8 | 8009639 |
| --- | -1.7 | --- | 8159900 |
| RP1-21O18.1 | -1.7 | kazrin | 8113491 |
| --- | -1.7 | --- | 8049963 |
| C9orf100 | -1.7 | chromosome 9 open reading frame 100 | 8130628 |
| EPHX2 | -1.7 | epoxide hydrolase 2, cytoplasmic | 8077262 |
| --- | -1.7 | --- | 7924443 |
| GSN | -1.7 | gelsolin (amyloidosis, Finnish type) | 8001748 |
| APOA1 | -1.7 | apolipoprotein A-I | 7957386 |
| APOA1 | -1.7 | apolipoprotein A-I | 7918284 |
| CKB | -1.7 | creatine kinase, brain | 8143610 |
| SNORA20 | -1.7 | small nucleolar RNA, H/ACA box 20 | 7957338 |
| PTMA | -1.6 | prothymosin, alpha (gene sequence 28) | 8084742 |
| HIST1H2AK | -1.6 | histone cluster 1, H2ak | 7960117 |
| CDCA7 | -1.6 | cell division cycle associated 7 | 8036956 |
| NANOS2 | -1.6 | nanos homolog 2 (Drosophila) | 7938370 |
| CCNB2 | -1.6 | cyclin B2 | 8005785 |
| --- | -1.6 | --- | 8088671 |
| TUBB4Q /// TUBB4Q | -1.6 | tubulin, beta polypeptide 4, member Q /// tubulin, beta polypeptide 4, member Q (TUBB4Q), mRNA | 7990442 |
| KIF15 | -1.6 | kinesin family member 15 | 7977854 |
| --- | -1.6 | --- | 8151494 |
| CCDC34 | -1.6 | coiled-coil domain containing 34 | 8020321 |
| --- | -1.6 | --- | 7923596 |
| --- | -1.6 | --- | 8109141 |
| HIST1H3C | -1.6 | histone cluster 1, H3c | 8145418 |
| SERPINA6 | -1.6 | serpin peptidase inhibitor, clade A (alpha-1 antiproteinase, antitrypsin), member 6 | 8096704 |
| HIST3H3 | -1.6 | histone cluster 3, H3 | 8138824 |
| CCNE1 /// CCNE1 | -1.6 | cyclin E1 /// cyclin E1 (CCNE1), transcript variant 2, mRNA | 8145570 |
| --- | -1.6 | --- | 8108478 |
| HMGB2 | -1.6 | high-mobility group box 2 | 7976073 |
| --- | -1.6 | --- | 8002152 |
| DNMT3B | -1.6 | DNA (cytosine-5-)-methyltransferase 3 beta | 8147057 |
| --- | -1.6 | --- | 8005685 |
| --- | -1.6 | --- | 7982712 |
| --- | -1.6 | --- | 8076547 |
| --- | -1.6 | --- | 8106827 |
| --- | -1.6 | --- | 8126666 |
| --- | -1.6 | --- | 8047097 |
| CDCA8 | -1.6 | cell division cycle associated 8 | 7933186 |
| GLRX | -1.6 | glutaredoxin (thioltransferase) | 8137524 |
| --- | -1.6 | --- | 8128956 |
| ASB9 | -1.6 | ankyrin repeat and SOCS box-containing 9 | 8172531 |
| TOP2A | -1.6 | topoisomerase (DNA) II alpha 170kDa | 8009746 |
| FBXO5 | -1.6 | F-box protein 5 | 8111218 |
| P4HA1 | -1.6 | procollagen-proline, 2-oxoglutarate 4-dioxygenase (proline 4-hydroxylase), alpha polypeptide I | 8008388 |
| BUB1 | -1.6 | BUB1 budding uninhibited by benzimidazoles 1 homolog (yeast) | 8013507 |
| ROR1 /// ROR1 | -1.6 | receptor tyrosine kinase-like orphan receptor 1 / variant 2, | 8005753 |
| --- | -1.6 | --- | 8104912 |
| HSP90AA2 | -1.6 | heat shock protein 90kDa alpha (cytosolic), class A member 2 | 7963174 |
| --- | -1.6 | --- | 8015835 |
| DPYD | -1.6 | dihydropyrimidine dehydrogenase | 8005446 |
| DLG7 | -1.6 | discs, large homolog 7 (Drosophila) | 8099253 |
| TUBB4Q /// LOC643224 | -1.6 | tubulin, beta polypeptide 4, member Q /// similar to tubulin, beta 8 | 8124518 |
| FABP5 | -1.6 | fatty acid binding protein 5 (psoriasis-associated) | 8026875 |
| --- | -1.6 | --- | 8168875 |
| --- | -1.6 | --- | 8040655 |
| HSD11B2 | -1.6 | hydroxysteroid (11-beta) dehydrogenase 2 | 8171170 |
| LOC389842 | -1.6 | similar to Ran-specific GTPase-activating protein (Ran-binding protein 1) (RanBP1) | 7899882 |
| FABP5 | -1.6 | fatty acid binding protein 5 (psoriasis-associated) | 7956759 |
| RNU5F | -1.6 | RNA, U5F small nuclear | 8146000 |
| --- | -1.6 | --- | 8104422 |
| DCXR | -1.6 | dicarbonyl/L-xylulose reductase | 7973377 |
| RP11-431O22.2 | -1.6 | Peptidylprolyl isomerase A (Cyclophilin A) (PPIA) pseudogene | 8070171 |
| HIST1H2BE | -1.6 | histone cluster 1, H2be | 8127743 |
| --- | -1.6 | --- | 7906564 |
| --- | -1.6 | --- | 8117382 |
| --- | -1.6 | --- | 7942071 |
| PECR | -1.6 | peroxisomal trans-2-enoyl-CoA reductase | 8070574 |
| AURKA | -1.6 | aurora kinase A | 7981566 |
| SOX5 /// SOX5 /// SOX5 | -1.6 | SRY (sex determining region Y)-box 5, transcript variant 3, mRNA /// transcript variant 2, mRNA | 8004266 |
| UBE2S | -1.6 | ubiquitin-conjugating enzyme E2S | 8149258 |
| --- | -1.6 | --- | 8148317 |
| --- | -1.6 | --- | 8056045 |
| HIST1H2AG | -1.6 | histone cluster 1, H2ag | 7956867 |
| --- | -1.6 | --- | 8087145 |
| HIST1H4B | -1.6 | histone cluster 1, H4b | 8007310 |
| --- | -1.6 | --- | 8041447 |
| --- | -1.6 | --- | 8089818 |
| --- | -1.6 | --- | 7964701 |
| --- | -1.6 | --- | 7960757 |
| --- | -1.6 | --- | 7910503 |
| SORD | -1.6 | sorbitol dehydrogenase | 7919591 |
| HSPE1 | -1.6 | heat shock 10kDa protein 1 (chaperonin 10) | 8137833 |
| --- | -1.6 | --- | 7973918 |
| NR4A2 /// NR4A2 | -1.6 | nuclear receptor subfamily 4, group A, member 2, transcript variant 3/4 mRNA | 8103389 |
| SCARNA12 | -1.6 | small Cajal body-specific RNA 12 | 7946851 |
| --- | -1.6 | --- | 8088167 |
| METTL7B | -1.6 | methyltransferase like 7B | 7983360 |
| FABP1 | -1.6 | fatty acid binding protein 1, liver | 8053562 |
| --- | -1.6 | --- | 8043995 |
| ITGAL | -1.6 | integrin, alpha L (antigen CD11A (p180), lymphocyte function-associated antigen 1; alpha polypeptide) | 8173430 |
| HRSP12 | -1.6 | heat-responsive protein 12 | 8039196 |
| ITPKA | -1.6 | inositol 1,4,5-trisphosphate 3-kinase A | 7923086 |
| HIST1H2AL | -1.6 | histone cluster 1, H2al | 7909586 |
| --- | -1.6 | --- | 8063547 |
| H2BFS | -1.6 | H2B histone family, member S | 7917875 |
| HIST4H4 | -1.6 | histone cluster 4, H4 | 8070239 |
| SLC25A11 | -1.6 | solute carrier family 25 (mitochondrial carrier; oxoglutarate carrier), member 11 | 8152115 |
| --- | -1.6 | --- | 8084299 |
| LOC731049 | -1.6 | similar to Ubiquitin-conjugating enzyme E2S ( E2-24 kDa) (Ubiquitin-protein ligase) (Ubiquitin carrier protein) (E2-EPF5) | 7953351 |
| --- | -1.6 | --- | 7989849 |
| --- | -1.6 | --- | 7963869 |
| --- | -1.6 | --- | 8018494 |
| HIST1H2BN | -1.6 | histone cluster 1, H2bn | 8078600 |
| HRBL | -1.6 | HIV-1 Rev binding protein-like | 7913869 |
| --- | -1.6 | --- | 7953812 |
| PON1 | -1.6 | paraoxonase 1 | 8124534 |
| --- | -1.6 | --- | 8071981 |
| CCDC88A | -1.6 | coiled-coil domain containing 88A | 7972745 |
| --- | -1.6 | --- | 8056693 |
| CYB5A | -1.6 | cytochrome b5 type A (microsomal) | 8081810 |
| FOXM1 | -1.6 | forkhead box M1 | 8003948 |
| --- | -1.6 | --- | 7969559 |
| CCDC86 | -1.6 | coiled-coil domain containing 86 | 8053668 |
| TMEM169 | -1.6 | transmembrane protein 169 | 7909527 |
| EHHADH | -1.6 | enoyl-Coenzyme A, hydratase/3-hydroxyacyl Coenzyme A dehydrogenase | 8035880 |
| NCAPD3 | -1.6 | non-SMC condensin II complex, subunit D3 | 8015060 |
| C21orf104 | -1.6 | C21orf104 | 7961022 |
| PCDH20 | -1.6 | protocadherin 20 | 8093128 |
| SNORA40 | -1.6 | small nucleolar RNA, H/ACA box 40 | 8019802 |
| PBK | -1.6 | PDZ binding kinase | 7995820 |
| AGTRL1 | -1.6 | angiotensin II receptor-like 1 | 8005441 |
| CASC5 /// CASC5 /// CASC5 | -1.6 | cancer susceptibility candidate 5, transcript variant 1// 2, | 7921088 |
| LOC647086 | -1.6 | similar to Protein C20orf27 | 7922550 |
| KIF4B | -1.6 | kinesin family member 4B | 8127787 |
| HIST1H3D /// HIST1H2AD | -1.6 | histone cluster 1, H3d /// histone cluster 1, H2ad | 8094789 |
| VWA1 | -1.6 | von Willebrand factor A domain containing 1 | 7940660 |
| HAL | -1.6 | histidine ammonia-lyase | 8103415 |
| BIRC5 | -1.6 | baculoviral IAP repeat-containing 5 (survivin) | 8026139 |
| DTYMK | -1.6 | deoxythymidylate kinase (thymidylate kinase) | 7986426 |
| AGPAT4 | -1.6 | 1-acylglycerol-3-phosphate O-acyltransferase 4 (lysophosphatidic acid acyltransferase, delta) | 8002571 |
| DTYMK | -1.6 | deoxythymidylate kinase (thymidylate kinase) | 7948995 |
| --- | -1.6 | --- | 7912975 |
| SNORA50 | -1.6 | small nucleolar RNA, H/ACA box 50 | 7970655 |
| PXMP2 | -1.6 | peroxisomal membrane protein 2, 22kDa | 8121886 |
| FAM19A4 | -1.6 | family with sequence similarity 19 (chemokine (C-C motif)-like), member A4 | 8053366 |
| --- | -1.6 | --- | 8122684 |
| --- | -1.6 | --- | 7949746 |
| ETNK2 | -1.6 | ethanolamine kinase 2 | 7938750 |
| CDCA2 | -1.6 | cell division cycle associated 2 | 7920664 |
| ESCO2 | -1.5 | establishment of cohesion 1 homolog 2 (S. cerevisiae) | 8073842 |
| --- | -1.5 | --- | 8149762 |
| C15orf23 | -1.5 | chromosome 15 open reading frame 23 | 7954604 |
| --- | -1.5 | --- | 8155048 |
| --- | -1.5 | --- | 8082058 |
| --- | -1.5 | --- | 8076461 |
| --- | -1.5 | --- | 8138545 |
| SKP2 | -1.5 | S-phase kinase-associated protein 2 (p45) | 8095163 |
| LOC339240 | -1.5 | keratin pseudogene | 8157036 |
| --- | -1.5 | --- | 7950235 |
| HIST1H2AJ | -1.5 | histone cluster 1, H2aj | 8100994 |
| SNORA68 | -1.5 | small nucleolar RNA, H/ACA box 68 | 8100179 |
| --- | -1.5 | --- | 7967841 |
| MTRR /// MTRR | -1.5 | 5-methyltetrahydrofolate-homocysteine methyltransferase reductase, transcript variant 1, mRNA | 7905088 |
| --- | -1.5 | --- | 8171802 |
| HMGN3 | -1.5 | high mobility group nucleosomal binding domain 3 | 7899480 |
| HIST1H2BD | -1.5 | histone cluster 1, H2bd | 8092931 |
| --- | -1.5 | --- | 8012207 |
| TFF2 | -1.5 | trefoil factor 2 (spasmolytic protein 1) | 8098870 |
| --- | -1.5 | --- | 8115664 |
| HMGA2 | -1.5 | high mobility group AT-hook 2 | 7976263 |
| LOC441795 | -1.5 | similar to high-mobility group box 3 | 7932938 |
| --- | -1.5 | --- | 8083777 |
| TRIM67 /// TRIM67 | -1.5 | tripartite motif-containing 67 /// tripartite motif-containing 67 (TRIM67), mRNA | 8150818 |
| FAM72D | -1.5 | Homo sapiens GCUD2 mRNA for Gastric cancer | 8031516 |
| --- | -1.5 | --- | 7981065 |
| LILRB3 /// LILRA6 /// LILRA6 /// LILRB3 | -1.5 | leukocyte immunoglobulin-like receptor, subfamily A/B (with TM domain), member 6 transcript variant 1, | 8139706 |
| ASPM | -1.5 | asp (abnormal spindle) homolog, microcephaly associated (Drosophila) | 8002143 |
| --- | -1.5 | --- | 7949060 |
| --- | -1.5 | --- | 7976239 |
| HSP90AA5P | -1.5 | heat shock protein 90kDa alpha (cytosolic), class A member 5 (pseudogene) | 8052798 |
| NCAPD2 | -1.5 | non-SMC condensin I complex, subunit D2 | 7962516 |
| STMN1 | -1.5 | stathmin 1/oncoprotein 18 | 7999553 |
| HIST1H4L | -1.5 | histone cluster 1, H4l | 8039409 |
| GLTPD2 | -1.5 | glycolipid transfer protein domain containing 2 | 7977046 |
| --- | -1.5 | --- | 8066513 |
| --- | -1.5 | --- | 8132070 |
| UQCRFS1 | -1.5 | ubiquinol-cytochrome c reductase, Rieske iron-sulfur polypeptide 1 | 7993622 |
| KRT24 | -1.5 | keratin 24 | 7935776 |
| PTMA | -1.5 | prothymosin, alpha | 8100109 |
| --- | -1.5 | --- | 8091799 |
| RNU2-2 | -1.5 | RNU2-2 | 7908178 |
| --- | -1.5 | --- | 8018906 |
| C15orf51 | -1.5 | chromosome 15 open reading frame 51 | 7963826 |
| ALDH4A1 | -1.5 | aldehyde dehydrogenase 4 family, member A1 | 8005510 |
| SUCLG1 | -1.5 | succinate-CoA ligase, alpha subunit | 8013292 |
| SUMO4 | -1.5 | SMT3 suppressor of mif two 3 homolog 4 (S. cerevisiae) | 8136918 |
| FLJ20699 /// FLJ20699 | -1.5 | hypothetical protein FLJ20699 /// hypothetical protein FLJ20699 (FLJ20699), mRNA | 7962487 |
| --- | -1.5 | --- | 8015806 |
| --- | -1.5 | --- | 7930454 |
| --- | -1.5 | --- | 8045289 |
| STARD10 /// STARD10 | -1.5 | StAR-related lipid transfer domain containing 10 // StAR-related lipid transfer domain containing 10, mRNA | 8161147 |
| HIST2H2AC | -1.5 | histone cluster 2, H2ac | 7970513 |
| SNORA73A | -1.5 | small nucleolar RNA, H/ACA box 73A | 7923027 |
| --- | -1.5 | --- | 8129985 |
| C17orf61 | -1.5 | chromosome 17 open reading frame 61 | 7936833 |
| GLRXL | -1.5 | glutaredoxin | 8008819 |
| SLC24A4 /// SLC24A4 | -1.5 | solute carrier family 24 (sodium/potassium/calcium exchanger), member 4, transcript variant 3, mRNA | 7909318 |
| LOC131055 | -1.5 | similar to peptidylprolyl isomerase A isoform 1 | 7955887 |
| SERPINA2 | -1.5 | serpine peptidase inhibitor | 7911092 |
| PPP1R14B | -1.5 | protein phosphatase 1, regulatory (inhibitor) subunit 14B | 8061373 |
| PTMAP7 | -1.5 | prothymosin, alpha pseudogene 7 | 8071361 |
| TMEM86B | -1.5 | transmembrane protein 86B | 7949615 |
| C3orf57 | -1.5 | chromosome 3 open reading frame 57 | 7960177 |
| --- | -1.5 | --- | 8109752 |
| PPP1R1A /// PPP1R1A | -1.5 | protein phosphatase 1, regulatory (inhibitor) subunit 1A | 8044813 |
| --- | -1.5 | --- | 8102848 |
| --- | -1.5 | --- | 8088172 |
| RACGAP1P | -1.5 | Rac GTPase activating protein 1 pseudogene | 8163149 |
| ETV4 /// ETV4 | -1.5 | ets variant gene 4 (E1A enhancer binding protein, E1AF) (ETV4), transcript variant 2, mRNA | 7960933 |
| C13orf3 | -1.5 | chromosome 13 open reading frame 3 | 7914805 |
| RPS26P39 | -1.5 | ribosomal protein S26 pseudogene 39 | 7945666 |
| C4BPA | -1.5 | complement component 4 binding protein, alpha | 8146171 |
| HOXC5 | -1.5 | homeobox C5 | 8135273 |
| --- | -1.5 | --- | 7907213 |
| B3GNT1 | -1.5 | UDP-GlcNAc:betaGal beta-1,3-N-acetylglucosaminyltransferase 1 | 8176174 |
| ODZ2 /// ODZ2 | -1.5 | odz, odd Oz/ten-m homolog 2 (Drosophila) /// odz, odd Oz/ten-m homolog 2 (Drosophila) (ODZ2), mRNA | 8129317 |
| TMEM37 | -1.5 | transmembrane protein 37 | 7939869 |
| --- | -1.5 | --- | 8116316 |
| --- | -1.5 | --- | 7925126 |
| --- | -1.5 | --- | 7998637 |
| OR4X1 | -1.5 | olfactory receptor, family 4, subfamily X, member 1 | 8024238 |
| --- | -1.5 | --- | 7915204 |
| SEPX1 /// SEPX1 | -1.5 | selenoprotein X, 1 /// selenoprotein X, 1 (SEPX1), mRNA | 7936242 |
| LOC728448 | -1.5 | peptidylprolyl isomerase E pseudogene | 8135099 |
| EMID2 /// EMID2 | -1.5 | EMI domain containing 2 /// EMI domain containing 2 (EMID2), mRNA | 8124211 |
| GPLD1 | -1.5 | glycosylphosphatidylinositol specific phospholipase D1 | 8063043 |
| UBE2C | -1.5 | ubiquitin-conjugating enzyme E2C | 8004043 |
| --- | -1.5 | --- | 8147371 |

RNA-Seq data

| Gene symbol | Fold change | Gene Name |
| --- | --- | --- |
| ANKRD1 | 6.4 | ankyrin repeat domain 1 (cardiac muscle) |
| NCF2 | 6.1 | neutrophil cytosolic factor 2 |
| NPPB | 6.1 | natriuretic peptide precursor B |
| IL8 | 4.5 | interleukin 8 |
| HDAC9 | 4.4 | histone deacetylase 9 |
| HKDC1 | 4.2 | hexokinase domain containing 1 |
| LAMC2 | 4.1 | laminin, gamma 2 |
| CXCL6 | 4.0 | chemokine (C-X-C motif) ligand 6 (granulocyte chemotactic protein 2) |
| UBASH3B | 3.9 | ubiquitin associated and SH3 domain containing, B |
| THBS1 | 3.8 | thrombospondin 1 |
| PRNP | 3.6 | prion protein |
| CREB5 | 3.6 | cAMP responsive element binding protein 5 |
| BDNF | 3.5 | brain-derived neurotrophic factor |
| ABCC9 | 3.5 | ATP-binding cassette, sub-family C (CFTR/MRP), member 9 |
| DSEL | 3.5 | dermatan sulfate epimerase-like |
| LINCR | 3.5 | neuralized homolog 3 (Drosophila) pseudogene |
| MATN2 | 3.5 | matrilin 2 |
| IFIH1 | 3.5 | interferon induced with helicase C domain 1 |
| ALPK3 | 3.4 | alpha-kinase 3 |
| ANXA3 | 3.4 | annexin A3 |
| FLNC | 3.4 | filamin C, gamma (actin binding protein 280) |
| PRSS23 | 3.3 | protease, serine, 23 |
| SAMD4A | 3.3 | sterile alpha motif domain containing 4A |
| MICAL2 | 3.3 | microtubule associated monoxygenase, calponin and LIM domain containing 2 |
| GAD1 | 3.3 | glutamate decarboxylase 1 (brain, 67kDa) |
| RASGEF1B | 3.2 | RasGEF domain family, member 1B |
| SOCS2 | 3.2 | suppressor of cytokine signaling 2 |
| RASD1 | 3.2 | RAS, dexamethasone-induced 1 |
| HSPB8 | 3.2 | heat shock 22kDa protein 8 |
| BHLHB3 | 3.2 | basic helix-loop-helix family, member e41 |
| TAGLN | 3.2 | transgelin |
| FER1L3 | 3.2 | fer-1-like 3, myoferlin |
| HABP2 | 3.2 | hyaluronic acid binding protein 2 |
| CXCL1 | 3.1 | chemokine (C-X-C motif) ligand 1 (melanoma growth stimulating activity, alpha) |
| PAPPA | 3.1 | pregnancy-associated plasma protein A, pappalysin 1 |
| PLA2G4C | 3.1 | phospholipase A2, group IVC (cytosolic, calcium-independent) |
| TUBA1A | 3.0 | tubulin, alpha 1a |
| BAI2 | 3.0 | brain-specific angiogenesis inhibitor 2 |
| LTBP1 | 3.0 | latent transforming growth factor beta binding protein 1 |
| ANXA1 | 3.0 | annexin A1 |
| PDGFB | 3.0 | platelet-derived growth factor beta polypeptide (simian sarcoma viral (v-sis) oncogene homolog) |
| GBP2 | 3.0 | guanylate binding protein 2, interferon-inducible |
| TAP1 | 3.0 | transporter 1, ATP-binding cassette, sub-family B (MDR/TAP) |
| VEPH1 | 3.0 | ventricular zone expressed PH domain homolog 1 (zebrafish) |
| TRIM9 | 2.9 | tripartite motif-containing 9 |
| FBXL16 | 2.9 | F-box and leucine-rich repeat protein 16 |
| C3orf32 | 2.9 | chromosome 3 open reading frame 32 |
| FZD8 | 2.9 | frizzled homolog 8 (Drosophila) |
| KRT23 | 2.9 | keratin 23 (histone deacetylase inducible) |
| OLFML3 | 2.8 | olfactomedin-like 3 |
| SLC16A14 | 2.8 | solute carrier family 16, member 14 |
| BACH2 | 2.8 | BTB and CNC homology 1, basic leucine zipper transcription factor 2 |
| CTGF | 2.8 | connective tissue growth factor |
| CYFIP2 | 2.7 | cytoplasmic FMR1 interacting protein 2 |
| NRP2 | 2.7 | neuropilin 2 |
| DUSP8 | 2.6 | dual specificity phosphatase 8 |
| CYR61 | 2.6 | cysteine-rich, angiogenic inducer, 61 |
| LAMA3 | 2.6 | laminin, alpha 3 |
| KLF4 | 2.6 | Kruppel-like factor 4 (gut) |
| TMEM45B | 2.6 | transmembrane protein 45B |
| TNNI1 | 2.6 | troponin I type 1 (skeletal, slow) |
| SLC5A10 | 2.6 | solute carrier family 5 (sodium/glucose cotransporter), member 10 |
| PRICKLE1 | 2.6 | prickle homolog 1 (Drosophila) |
| PPL | 2.6 | periplakin |
| PHLDA1 | 2.5 | pleckstrin homology-like domain, family A, member 1 |
| VNN3 | 2.5 | vanin 3 |
| ADPRH | 2.5 | ADP-ribosylarginine hydrolase |
| SERPINE2 | 2.5 | serpin peptidase inhibitor, clade E (nexin, plasminogen activator inhibitor type 1), member 2 |
| AMOTL2 | 2.5 | angiomotin like 2 |
| GALNAC4S-6ST | 2.4 | carbohydrate (N-acetylgalactosamine 4-sulfate 6-O) sulfotransferase 15 |
| LEPREL1 | 2.4 | leprecan-like 1 |
| ATF3 | 2.4 | activating transcription factor 3 |
| TNFRSF12A | 2.4 | tumor necrosis factor receptor superfamily, member 12A |
| NTN4 | 2.4 | netrin 4 |
| SUSD4 | 2.4 | sushi domain containing 4 |
| UBD | 2.4 | ubiquitin D |
| DTNA | 2.4 | dystrobrevin, alpha |
| SLC6A8 | 2.4 | solute carrier family 6 (neurotransmitter transporter, creatine), member 8 |
| TBX15 | 2.4 | T-box 15 |
| SLC34A2 | 2.4 | solute carrier family 34 (sodium phosphate), member 2 |
| BIRC3 | 2.4 | baculoviral IAP repeat-containing 3 |
| CRIM1 | 2.4 | cysteine rich transmembrane BMP regulator 1 (chordin-like) |
| FGD3 | 2.4 | FYVE, RhoGEF and PH domain containing 3 |
| SLC16A12 | 2.3 | solute carrier family 16, member 12 (monocarboxylic acid transporter 12) |
| TNFRSF19 | 2.3 | tumor necrosis factor receptor superfamily, member 19 |
| WTIP | 2.3 | Wilms tumor 1 interacting protein |
| SPAG1 | 2.3 | sperm associated antigen 1 |
| FLJ20489 | 2.3 | solute carrier family 48 (heme transporter), member 1 |
| IL32 | 2.3 | interleukin 32 |
| C10orf54 | 2.3 | chromosome 10 open reading frame 54 |
| SLC44A3 | 2.3 | solute carrier family 44, member 3 |
| MAFK | 2.3 | v-maf musculoaponeurotic fibrosarcoma oncogene homolog K (avian) |
| CAPN2 | 2.3 | castor zinc finger 1 |
| FSTL1 | 2.3 | follistatin-like 1 |
| GADD45B | 2.3 | growth arrest and DNA-damage-inducible, beta |
| OLFML2A | 2.3 | olfactomedin-like 2A |
| THSD4 | 2.3 | thrombospondin, type I, domain containing 4 |
| TPM1 | 2.2 | tropomyosin 1 (alpha) |
| ELF3 | 2.2 | E74-like factor 3 (ets domain transcription factor, epithelial-specific ) |
| PDE5A | 2.2 | phosphodiesterase 5A, cGMP-specific |
| PLK2 | 2.2 | polo-like kinase 2 (Drosophila) |
| LMCD1 | 2.2 | LIM and cysteine-rich domains 1 |
| C1QTNF5 | 2.2 | C1q and tumor necrosis factor related protein 5 |
| IRF1 | 2.2 | interferon regulatory factor 1 |
| ADCYAP1 | 2.2 | adenylate cyclase activating polypeptide 1 (pituitary) |
| KIF5C | 2.2 | kinesin family member 5C |
| CDKN1C | 2.2 | cyclin-dependent kinase inhibitor 1C (p57, Kip2) |
| KIAA0746 | 2.2 | KIAA0746 |
| RFTN1 | 2.2 | raftlin, lipid raft linker 1 |
| JAG1 | 2.2 | jagged 1 (Alagille syndrome) |
| MID2 | 2.2 | midline 2 |
| FOSL2 | 2.2 | FOS-like antigen 2 |
| SPG3A | 2.2 | spastic paraplegia 3A homolog |
| ACCN3 | 2.2 | amiloride-sensitive cation channel 3 |
| F2RL1 | 2.2 | coagulation factor II (thrombin) receptor-like 1 |
| ZNF114 | 2.2 | zinc finger protein 114 |
| S100A6 | 2.1 | S100 calcium binding protein A6 |
| HOXA1 | 2.1 | homeobox A1 |
| PCYT1B | 2.1 | phosphate cytidylyltransferase 1, choline, beta |
| SPECC1 | 2.1 | cytospin B |
| EIF4E3 | 2.1 | eukaryotic translation initiation factor 4E family member 3 |
| ENO2 | 2.1 | enolase 2 (gamma, neuronal) |
| MBOAT1 | 2.1 | membrane bound O-acyltransferase domain containing 1 |
| NDRG4 | 2.1 | NDRG family member 4 |
| SPSB4 | 2.1 | splA/ryanodine receptor domain and SOCS box containing 4 |
| VNN2 | 2.1 | vanin 2 |
| ZNF233 | 2.1 | zinc finger protein 233 |
| NRG3 | 2.1 | neuregulin 3 |
| BMP8B | 2.1 | bone morphogenetic protein 8b |
| SLC29A3 | 2.1 | solute carrier family 29 (nucleoside transporters), member 3 |
| RHBDF1 | 2.1 | rhomboid 5 homolog 1 (Drosophila) |
| COL16A1 | 2.1 | collagen, type XVII, alpha 1 |
| HYI | 2.1 | hydroxypyruvate isomerase homolog |
| MBNL2 | 2.1 | muscleblind-like 2 (Drosophila) |
| GPR137B | 2.1 | G protein-coupled receptor 137B |
| IDUA | 2.1 | iduronidase, alpha-L- |
| FLJ44968 | 2.1 | YjeF N-terminal domain containing 3 |
| RBP7 | 2.1 | retinol binding protein 7, cellular |
| TSHZ2 | 2.1 | teashirt zinc finger homeobox 2 |
| SDPR | 2.1 | serum deprivation response (phosphatidylserine binding protein) |
| CSF1 | 2.1 | colony stimulating factor 1 (macrophage) |
| F2RL2 | 2.1 | coagulation factor II (thrombin) receptor-like 2 |
| FGF12 | 2.1 | fibroblast growth factor 12 |
| LOXL4 | 2.1 | lysyl oxidase-like 4 |
| OPRL1 | 2.1 | opiate receptor-like 1 |
| GPC5 | 2.1 | glypican 5 |
| SLC12A2 | 2.1 | solute carrier family 12 (sodium/potassium/chloride transporters), member 2 |
| AQP7 | 2.1 | aquaporin 7 |
| CHST3 | 2.0 | carbohydrate (chondroitin 6) sulfotransferase 3 |
| HIVEP2 | 2.0 | H2.0-like homeobox |
| LYPD6 | 2.0 | lysozyme G-like 1 |
| STX11 | 2.0 | syntaxin 11 |
| FZD5 | 2.0 | frizzled homolog 5 (Drosophila) |
| STX1A | 2.0 | syntaxin 1A (brain) |
| PNMA3 | 2.0 | paraneoplastic antigen MA3 |
| BMP2 | 2.0 | bone morphogenetic protein 2 |
| MX1 | 2.0 | myxovirus (influenza virus) resistance 1, interferon-inducible protein p78 (mouse) |
| IL17RD | 2.0 | interleukin 17 receptor D |
| ANXA13 | 2.0 | annexin A13 |
| PTPRB | 2.0 | protein tyrosine phosphatase, receptor type, B |
| C6orf1 | 2.0 | chromosome 6 open reading frame 1 |
| TUBB3 | 2.0 | tubulin, beta 3 |
| CX3CL1 | 2.0 | chemokine (C-X3-C motif) ligand 1 |
| KCNJ8 | 2.0 | potassium inwardly-rectifying channel, subfamily J, member 8 |
| MAPK8IP1 | 2.0 | mitogen-activated protein kinase 8 interacting protein 1 |
| RASGRP3 | 2.0 | RAS guanyl releasing protein 3 (calcium and DAG-regulated) |
| EFEMP1 | 2.0 | EGF-containing fibulin-like extracellular matrix protein 1 |
| OXTR | 2.0 | oxytocin receptor |
| DUSP4 | 2.0 | dual specificity phosphatase 4 |
| TIGD6 | 2.0 | tigger transposable element derived 6 |
| METRNL | 2.0 | methyltransferase like 7A |
| TNFAIP2 | 2.0 | tumor necrosis factor, alpha-induced protein 2 |
| N4BP3 | 2.0 | Nedd4 binding protein 3 |
| SYTL5 | 2.0 | synaptotagmin-like 5 |
| KLF11 | 2.0 | Kruppel-like factor 11 |
| SIPA1L2 | 2.0 | signal-induced proliferation-associated 1 like 2 |
| KLHL5 | 2.0 | kelch-like 5 (Drosophila) |
| ANKRD24 | 2.0 | ankyrin repeat domain 24 |
| GPRC5B | 2.0 | G protein-coupled receptor, family C, group 5, member B |
| RCN1 | 2.0 | reticulocalbin 1, EF-hand calcium binding domain |
| MAP3K14 | 2.0 | mitogen-activated protein kinase kinase kinase 14 |
| IRS2 | 2.0 | insulin receptor substrate 2 |
| ANKRD29 | 2.0 | ankyrin repeat domain 29 |
| IFITM3 | 2.0 | interferon induced transmembrane protein 3 (1-8U) |
| NPNT | 1.9 | nephronectin |
| JUB | 1.9 | jub, ajuba homolog (Xenopus laevis) |
| GCNT3 | 1.9 | glucosaminyl (N-acetyl) transferase 3, mucin type |
| P2RY2 | 1.9 | purinergic receptor P2Y, G-protein coupled, 2 |
| OCA2 | 1.9 | oculocutaneous albinism II |
| MYL9 | 1.9 | myosin, light chain 9, regulatory |
| C15orf52 | 1.9 | chromosome 15 open reading frame 52 |
| LRRC69 | 1.9 | leucine rich repeat containing 69 |
| FSTL3 | 1.9 | follistatin-like 3 (secreted glycoprotein) |
| SMAD7 | 1.9 | SMAD family member 7 |
| NPR2 | 1.9 | natriuretic peptide receptor B/guanylate cyclase B (atrionatriuretic peptide receptor B) |
| TNFRSF14 | 1.9 | tumor necrosis factor receptor superfamily, member 14 (herpesvirus entry mediator) |
| S100P | 1.9 | S100 calcium binding protein P |
| NEDD9 | 1.9 | neural precursor cell expressed, developmentally down-regulated 9 |
| FLNA | 1.9 | filamin A, alpha (actin binding protein 280) |
| GPR39 | 1.9 | G protein-coupled receptor 39 |
| SPINK1 | 1.9 | serine peptidase inhibitor, Kazal type 1 |
| FBLN5 | 1.9 | fibulin 5 |
| C17orf28 | 1.9 | chromosome 17 open reading frame 28 |
| CDKN2B | 1.9 | cyclin-dependent kinase inhibitor 2B (p15, inhibits CDK4) |
| FAM125B | 1.9 | family with sequence similarity 125, member B |
| PPP1R1A | 1.9 | protein phosphatase 1, regulatory (inhibitor) subunit 1A |
| HEG1 | 1.9 | hemochromatosis type 2 (juvenile) |
| VIM | 1.9 | vimentin |
| SYNPO | 1.9 | synaptopodin |
| UNC5B | 1.9 | unc-5 homolog B (C. elegans) |
| ABLIM3 | 1.9 | actin binding LIM protein family, member 3 |
| ARSJ | 1.9 | arylsulfatase family, member J |
| KIAA0649 | 1.9 | KIAA0649 |
| GLIS3 | 1.9 | GLIS family zinc finger 3 |
| MRM1 | 1.9 | mitochondrial rRNA methyltransferase 1 homolog (S. cerevisiae) |
| TRIM62 | 1.9 | tripartite motif-containing 62 |
| GDF15 | 1.9 | growth differentiation factor 15 |
| TGFB2 | 1.9 | transforming growth factor, beta 2 |
| MYO7A | 1.9 | myosin VIIA |
| THBS3 | 1.9 | thrombospondin 3 |
| HBEGF | 1.9 | heparin-binding EGF-like growth factor |
| ZP3 | 1.9 | zona pellucida glycoprotein 3 (sperm receptor) |
| DUSP1 | 1.9 | dual specificity phosphatase 1 |
| ICAM1 | 1.9 | intercellular adhesion molecule 1 |
| ABR | 1.9 | active BCR-related gene |
| TNFAIP3 | 1.9 | tumor necrosis factor, alpha-induced protein 3 |
| EPPK1 | 1.8 | epiplakin 1 |
| TMEM185A | 1.8 | transmembrane protein 185A |
| KLF6 | 1.8 | Kruppel-like factor 6 |
| MRAS | 1.8 | muscle RAS oncogene homolog |
| SEMA3B | 1.8 | sema domain, immunoglobulin domain (Ig), short basic domain, secreted, (semaphorin) 3B |
| ELFN1 | 1.8 | extracellular leucine-rich repeat and fibronectin type III domain containing 1 |
| L3MBTL4 | 1.8 | l(3)mbt-like 4 (Drosophila) |
| CDH19 | 1.8 | cadherin 19, type 2 |
| NPAL2 | 1.8 | NIPA-like domain containing 2 |
| PLCD3 | 1.8 | phospholipase C, delta 3 |
| TCP11L2 | 1.8 | t-complex 11 (mouse)-like 2 |
| NFKBIE | 1.8 | nuclear factor of kappa light polypeptide gene enhancer in B-cells inhibitor, epsilon |
| PARP12 | 1.8 | poly (ADP-ribose) polymerase family, member 12 |
| IL18 | 1.8 | interleukin 18 (interferon-gamma-inducing factor) |
| RPS6KL1 | 1.8 | ribosomal protein S6 kinase-like 1 |
| RND3 | 1.8 | Rho family GTPase 3 |
| SVIL | 1.8 | supervillin |
| SYT11 | 1.8 | synaptotagmin XI |
| PPP1R15A | 1.8 | protein phosphatase 1, regulatory (inhibitor) subunit 15A |
| C1orf91 | 1.8 | chromosome 1 open reading frame 91 |
| MOSPD1 | 1.8 | motile sperm domain containing 1 |
| FAM59A | 1.8 | family with sequence similarity 59, member A |
| TNS3 | 1.8 | tensin 3 |
| INHBE | 1.8 | inhibin, beta E |
| LASS1 | 1.8 | LAG1 homolog, ceramide synthase 1 |
| GABRB1 | 1.8 | gamma-aminobutyric acid (GABA) A receptor, beta 1 |
| RAB17 | 1.8 | RAB17, member RAS oncogene family |
| OSGIN1 | 1.8 | oxidative stress induced growth inhibitor 1 |
| LBH | 1.8 | limb bud and heart development homolog (mouse) |
| LOC440567 | 1.8 | LOC440567 |
| TUBB2B | 1.8 | tubulin, beta 2B |
| MCOLN3 | 1.8 | mucolipin 3 |
| MPP1 | 1.8 | membrane protein, palmitoylated 1, 55kDa |
| CLDN6 | 1.8 | claudin 6 |
| PARD3B | 1.8 | par-3 partitioning defective 3 homolog B (C. elegans) |
| EDNRB | 1.8 | endothelin receptor type B |
| RELB | 1.8 | v-rel reticuloendotheliosis viral oncogene homolog B |
| MCAM | 1.8 | melanoma cell adhesion molecule |
| HOXA3 | 1.8 | homeobox A3 |
| LARP6 | 1.8 | La ribonucleoprotein domain family, member 6 |
| MYOM1 | 1.8 | myomesin 1, 185kDa |
| EPHA2 | 1.8 | EPH receptor A2 |
| FLVCR2 | 1.8 | feline leukemia virus subgroup C cellular receptor family, member 2 |
| CCDC80 | 1.8 | coiled-coil domain containing 80 |
| WWC1 | 1.8 | WW and C2 domain containing 1 |
| KLF10 | 1.8 | Kruppel-like factor 10 |
| SP100 | 1.8 | SP100 nuclear antigen |
| SLC19A3 | 1.8 | solute carrier family 19, member 3 |
| DAAM1 | 1.8 | dishevelled associated activator of morphogenesis 1 |
| CCR1 | 1.8 | chemokine (C-C motif) receptor 1 |
| FBXO6 | 1.8 | F-box protein 6 |
| SH3RF1 | 1.8 | SH3 domain containing ring finger 1 |
| FILIP1L | 1.8 | filamin A interacting protein 1-like |
| SNCA | 1.8 | synuclein, alpha (non A4 component of amyloid precursor) |
| RAPGEF5 | 1.8 | Rap guanine nucleotide exchange factor (GEF) 5 |
| BICC1 | 1.8 | bicaudal C homolog 1 (Drosophila) |
| TBX2 | 1.8 | T-box 2 |
| CYGB | 1.8 | cytoglobin |
| HAPLN4 | 1.8 | hyaluronan and proteoglycan link protein 4 |
| PTPN21 | 1.8 | protein tyrosine phosphatase, non-receptor type 21 |
| SPINT1 | 1.8 | serine peptidase inhibitor, Kunitz type 1 |
| TMEM54 | 1.8 | transmembrane protein 54 |
| STK17A | 1.8 | serine/threonine kinase 17a |
| JUN | 1.8 | jun oncogene |
| OBFC2A | 1.8 | oligonucleotide/oligosaccharide-binding fold containing 2A |
| TBC1D9 | 1.8 | TBC1 domain family, member 9 (with GRAM domain) |
| ANK2 | 1.7 | ankyrin 2, neuronal |
| CLK4 | 1.7 | ceroid-lipofuscinosis, neuronal 8 (epilepsy, progressive with mental retardation) |
| TMEM2 | 1.7 | transmembrane protein 2 |
| GEM | 1.7 | GTP binding protein overexpressed in skeletal muscle |
| GRK5 | 1.7 | G protein-coupled receptor kinase 5 |
| PDGFA | 1.7 | platelet-derived growth factor alpha polypeptide |
| TNFRSF11B | 1.7 | tumor necrosis factor receptor superfamily, member 11B |
| KIAA1609 | 1.7 | KIAA1609 |
| TESC | 1.7 | tescalcin |
| CLTCL1 | 1.7 | connector enhancer of kinase suppressor of Ras 2 |
| CASP4 | 1.7 | Caspase 4 |
| PEA15 | 1.7 | phosphoprotein enriched in astrocytes 15 |
| GPR137C | 1.7 | G protein-coupled receptor 137C |
| DLC1 | 1.7 | deleted in liver cancer 1 |
| NPTXR | 1.7 | neuronal pentraxin receptor |
| RLTPR | 1.7 | RGD motif, leucine rich repeats, tropomodulin domain and proline-rich containing |
| FAM134B | 1.7 | family with sequence similarity 134, member B |
| TMEM16D | 1.7 | transmembrane protein 16D |
| GPR161 | 1.7 | G protein-coupled receptor 161 |
| PDK4 | 1.7 | pyruvate dehydrogenase kinase, isozyme 4 |
| PIB5PA | 1.7 | inositol polyphosphate-5-phosphatase J |
| YPEL3 | 1.7 | yippee-like 3 (Drosophila) |
| ULBP1 | 1.7 | UL16 binding protein 1 |
| RTN1 | 1.7 | reticulon 1 |
| LITAF | 1.7 | lipopolysaccharide-induced TNF factor |
| GPR158 | 1.7 | G protein-coupled receptor 161 |
| SERPINA3 | 1.7 | serpin peptidase inhibitor, clade A (alpha-1 antiproteinase, antitrypsin), member 3 |
| PRAGMIN | 1.7 | homolog of rat pragma of Rnd2 |
| DMPK | 1.7 | dystrophia myotonica-protein kinase |
| AFF3 | 1.7 | anterior gradient homolog 2 (Xenopus laevis) |
| KCTD11 | 1.7 | potassium channel tetramerisation domain containing 11 |
| SH3YL1 | 1.7 | SH3 domain containing, Ysc84-like 1 (S. cerevisiae) |
| CXCL5 | 1.7 | chemokine (C-X-C motif) ligand 5 |
| LOC492311 | 1.7 | LOC492311 |
| TMEM200B | 1.7 | transmembrane protein 200B |
| TINAGL1 | 1.7 | tubulointerstitial nephritis antigen-like 1 |
| QRICH2 | 1.7 | glutamine rich 2 |
| PIK3CD | 1.7 | phosphoinositide-3-kinase, catalytic, delta polypeptide |
| C20orf117 | 1.7 | chromosome 20 open reading frame 117 |
| ABLIM2 | 1.7 | actin binding LIM protein family, member 2 |
| IER3 | 1.7 | immediate early response 3 |
| DAPK2 | 1.7 | death-associated protein kinase 2 |
| EHD4 | 1.7 | EH-domain containing 4 |
| FZD4 | 1.7 | frizzled homolog 4 (Drosophila) |
| PDE3A | 1.7 | phosphodiesterase 3A, cGMP-inhibited |
| RRAGC | 1.7 | Ras-related GTP binding C |
| NOSTRIN | 1.7 | nitric oxide synthase trafficker |
| FLJ20160 | 1.7 | major facilitator superfamily domain containing 6 |
| ARHGEF17 | 1.7 | Rho guanine nucleotide exchange factor (GEF) 17 |
| GDPD5 | 1.7 | glycerophosphodiester phosphodiesterase domain containing 5 |
| SPTAN1 | 1.7 | spectrin, alpha, non-erythrocytic 1 (alpha-fodrin) |
| ENPP5 | 1.7 | ectonucleotide pyrophosphatase/phosphodiesterase 5 (putative function) |
| FLJ20674 | 1.7 | V-set and immunoglobulin domain containing 10 |
| CHST9 | 1.7 | carbohydrate (N-acetylgalactosamine 4-0) sulfotransferase 9 |
| ARHGEF2 | 1.7 | rho/rac guanine nucleotide exchange factor (GEF) 2 |
| ACTB | 1.7 | actin, beta |
| PIM1 | 1.7 | pim-1 oncogene |
| KIAA1754 | 1.7 | KIAA1754 |
| FLRT2 | 1.7 | fibronectin leucine rich transmembrane protein 2 |
| FAM171B | 1.7 | family with sequence similarity 171, member B |
| SYDE1 | 1.7 | synapse defective 1, Rho GTPase, homolog 1 (C. elegans) |
| RHOC | 1.7 | ras homolog gene family, member C |
| PDE9A | 1.7 | phosphodiesterase 9A |
| TRIM23 | 1.7 | tripartite motif-containing 23 |
| LRRN1 | 1.7 | leucine rich repeat neuronal 1 |
| NUAK2 | 1.7 | NUAK family, SNF1-like kinase, 2 |
| WWTR1 | 1.7 | WW domain containing transcription regulator 1 |
| SRGAP1 | 1.7 | SLIT-ROBO Rho GTPase activating protein 1 |
| ITPR3 | 1.7 | inositol 1,4,5-triphosphate receptor, type 3 |
| KRT8 | 1.7 | keratin 8 |
| RBPMS | 1.7 | RNA binding protein with multiple splicing |
| MATN3 | 1.7 | matrilin 3 |
| RNF19A | 1.7 | ring finger protein 19A |
| KRT19 | 1.7 | keratin 19 |
| FAM38B | 1.7 | family with sequence similarity 38, member B |
| C6orf145 | 1.7 | chromosome 6 open reading frame 145 |
| S1PR3 | 1.7 | sphingosine-1-phosphate receptor 3 |
| RHBDF2 | 1.7 | rhomboid 5 homolog 2 (Drosophila) |
| GLRB | 1.7 | glycine receptor, beta |
| BEX2 | 1.7 | brain expressed X-linked 2 |
| AIM1 | 1.7 | aldo-keto reductase family 1, member C3 (3-alpha hydroxysteroid dehydrogenase, type II) |
| RAB15 | 1.7 | RAB15, member RAS onocogene family |
| ARL4C | 1.7 | ADP-ribosylation factor-like 4C |
| ARHGEF5 | 1.7 | Rho guanine nucleotide exchange factor (GEF) 5 |
| SLC1A3 | 1.7 | solute carrier family 1 (glial high affinity glutamate transporter), member 3 |
| NBEA | 1.7 | neurobeachin |
| STBD1 | 1.7 | starch binding domain 1 |
| BTN2A2 | 1.7 | butyrophilin, subfamily 2, member A2 |
| ADAM22 | 1.6 | ADAM metallopeptidase domain 22 |
| MAP3K1 | 1.6 | mitogen-activated protein kinase kinase kinase 1 |
| RAPGEF4 | 1.6 | Rap guanine nucleotide exchange factor (GEF) 4 |
| ZNF20 | 1.6 | zinc finger protein 20 |
| ANKS1B | 1.6 | ankyrin repeat and sterile alpha motif domain containing 1B |
| PHLDB1 | 1.6 | pleckstrin homology-like domain, family B, member 1 |
| RAB6B | 1.6 | RAB6B, member RAS oncogene family |
| NFKB2 | 1.6 | nuclear factor of kappa light polypeptide gene enhancer in B-cells 2 (p49/p100) |
| CD55 | 1.6 | CD55 molecule, decay accelerating factor for complement (Cromer blood group) |
| TUBB2A | 1.6 | tubulin, beta 2A |
| RAP1GAP | 1.6 | RAP1 GTPase activating protein |
| TNFAIP8 | 1.6 | tumor necrosis factor, alpha-induced protein 8 |
| ABTB1 | 1.6 | ankyrin repeat and BTB (POZ) domain containing 1 |
| STEAP3 | 1.6 | STEAP family member 3 |
| SPTBN5 | 1.6 | spectrin, beta, non-erythrocytic 5 |
| GCNT4 | 1.6 | glucosaminyl (N-acetyl) transferase 4, core 2 (beta-1,6-N-acetylglucosaminyltransferase) |
| YPEL2 | 1.6 | yippee-like 2 (Drosophila) |
| BCAR1 | 1.6 | breast cancer anti-estrogen resistance 1 |
| PWWP2B | 1.6 | PWWP domain containing 2B |
| DPYSL3 | 1.6 | dihydropyrimidinase-like 3 |
| CEBPD | 1.6 | CCAAT/enhancer binding protein (C/EBP), delta |
| ROBO2 | 1.6 | roundabout, axon guidance receptor, homolog 2 (Drosophila) |
| CASZ1 | 1.6 | castor zinc finger 1 |
| C19orf26 | 1.6 | chromosome 19 open reading frame 26 |
| GTPBP2 | 1.6 | GTP binding protein 2 |
| KRT18 | 1.6 | keratin 18 |
| MANBA | 1.6 | mannosidase, beta A, lysosomal |
| DENND2A | 1.6 | DENN/MADD domain containing 2A |
| CACNG4 | 1.6 | calcium channel, voltage-dependent, gamma subunit 4 |
| LAD1 | 1.6 | ladinin 1 |
| LCA5 | 1.6 | Leber congenital amaurosis 5 |
| INPP5F | 1.6 | inositol polyphosphate-5-phosphatase F |
| PROM1 | 1.6 | prominin 1 |
| PPP1R13B | 1.6 | protein phosphatase 1, regulatory (inhibitor) subunit 13B |
| NCK2 | 1.6 | NCK adaptor protein 2 |
| PACS1 | 1.6 | phosphofurin acidic cluster sorting protein 1 |
| SMAD9 | 1.6 | SMAD family member 9 |
| CTSL1 | 1.6 | cathepsin L1 |
| ARVCF | 1.6 | armadillo repeat gene deletes in velocardiofacial syndrome |
| WWC2 | 1.6 | WW and C2 domain containing 2 |
| GPR153 | 1.6 | G protein-coupled receptor 158 |
| DACT2 | 1.6 | dapper, antagonist of beta-catenin, homolog 2 (Xenopus laevis) |
| SSTR2 | 1.6 | somatostatin receptor 2 |
| NID2 | 1.6 | nidogen 2 (osteonidogen) |
| TIMP4 | 1.6 | TIMP metallopeptidase inhibitor 4 |
| NMT2 | 1.6 | N-myristoyltransferase 2 |
| PPAP2A | 1.6 | phosphatidic acid phosphatase type 2A |
| PLXNC1 | 1.6 | plexin C1 |
| GOLSYN | 1.6 | Golgi-localized protein |
| DAPK3 | 1.6 | death-associated protein kinase 3 |
| KIFC2 | 1.6 | kinesin family member C2 |
| TBX19 | 1.6 | T-box 19 |
| CCL20 | 1.6 | chemokine (C-C motif) ligand 20 |
| TUFT1 | 1.6 | tuftelin 1 |
| LRCH1 | 1.6 | leucine-rich repeats and calponin homology (CH) domain containing 1 |
| ENC1 | 1.6 | ectodermal-neural cortex (with BTB-like domain) |
| DKK3 | 1.6 | dickkopf homolog 3 (Xenopus laevis) |
| MAFF | 1.6 | v-maf musculoaponeurotic fibrosarcoma oncogene homolog F (avian) |
| EPHA5 | 1.6 | EPH receptor A5 |
| COL7A1 | 1.6 | collagen, type VIII, alpha 2 |
| SAMD12 | 1.6 | sterile alpha motif domain containing 12 |
| ARL2 | 1.6 | ADP-ribosylation factor-like 2 |
| KIAA1949 | 1.6 | KIAA1949 |
| UBE2L6 | 1.6 | ubiquitin-conjugating enzyme E2L 6 |
| FUT8 | 1.6 | fucosyltransferase 8 (alpha (1,6) fucosyltransferase) |
| ALX1 | 1.6 | angiopoietin-like 2 |
| CLDN1 | 1.6 | claudin 1 |
| HABP4 | 1.6 | hyaluronan binding protein 4 |
| CDH6 | 1.6 | cadherin 6, type 2, K-cadherin (fetal kidney) |
| PHF7 | 1.6 | PHD finger protein 7 |
| CDC14A | 1.6 | CDC14 cell division cycle 14 homolog A (S. cerevisiae) |
| MYC | 1.6 | v-myc myelocytomatosis viral oncogene homolog (avian) |
| C22orf36 | 1.6 | chromosome 22 open reading frame 36 |
| STAT5A | 1.6 | signal transducer and activator of transcription 5A |
| DPP4 | 1.6 | dipeptidyl-peptidase 4 |
| ADARB1 | 1.6 | adenosine deaminase, RNA-specific, B1 (RED1 homolog rat) |
| FAM65A | 1.6 | family with sequence similarity 65, member A |
| SEMA6A | 1.6 | sema domain, transmembrane domain (TM), and cytoplasmic domain, (semaphorin) 6A |
| PTPRU | 1.6 | protein tyrosine phosphatase, receptor type, U |
| ANGPTL2 | 1.6 | angiopoietin-like 2 |
| FAM50A | 1.6 | family with sequence similarity 50, member A |
| FUNDC2 | 1.6 | FUN14 domain containing 2 |
| EPB49 | 1.6 | erythrocyte membrane protein band 4.9 (dematin) |
| HSPA1L | 1.6 | heat shock 70kDa protein 1-like |
| ZAK | 1.6 | sterile alpha motif and leucine zipper containing kinase AZK |
| HECA | 1.6 | headcase homolog (Drosophila) [*Homo sapiens*] |
| KIF3C | 1.6 | kinesin family member 3C |
| RAPGEF2 | 1.6 | Rap guanine nucleotide exchange factor (GEF) 2 |
| C5orf41 | 1.6 | chromosome 5 open reading frame 41 |
| CLN8 | 1.6 | Claudin 8 |
| SKAP2 | 1.6 | src kinase associated phosphoprotein 2 |
| FKBP1B | 1.6 | FK506 binding protein 1B, 12.6 kDa |
| STC2 | 1.6 | stanniocalcin 2 |
| RPL39 | 1.6 | ribosomal protein L39 |
| CAV1 | 1.6 | caveolin 1 |
| ZNF350 | 1.6 | zinc finger protein 350 |
| RAB32 | 1.6 | RAB32, member RAS oncogene family |
| VLDLR | 1.6 | very low density lipoprotein receptor |
| TRIM3 | 1.6 | tripartite motif-containing 3 |
| C9orf19 | 1.6 | chromosome 9 open reading frame 19 |
| ASAH1 | 1.6 | N-acylsphingosine amidohydrolase (acid ceramidase) 1 |
| C13orf31 | 1.6 | chromosome 13 open reading frame 31 |
| SYNGR3 | 1.6 | synaptogyrin 3 |
| NFKBIA | 1.6 | nuclear factor of kappa light polypeptide gene enhancer in B-cells inhibitor, alpha |
| SLC12A4 | 1.6 | solute carrier family 12 (potassium/chloride transporters), member 4 |
| LOC283755 | 1.6 | LOC283755 |
| SEMA7A | 1.6 | semaphorin 7A, GPI membrane anchor (John Milton Hagen blood group) |
| CYP1A1 | 1.6 | cytochrome P450, family 1, subfamily A, polypeptide 1 |
| LOC390637 | 1.6 | LOC390637 |
| SLC25A30 | 1.6 | solute carrier family 25, member 30 |
| NPC2 | 1.6 | Niemann-Pick disease, type C2 |
| CDH2 | 1.6 | cadherin 2, type 1, N-cadherin (neuronal) |
| C12orf39 | 1.6 | chromosome 12 open reading frame 52 |
| ZC3H12C | 1.6 | zinc finger CCCH-type containing 12C |
| CLK1 | 1.6 | CDC-like kinase 4 |
| BTN3A1 | 1.6 | butyrophilin, subfamily 3, member A1 |
| MICAL1 | 1.6 | microtubule associated monoxygenase, calponin and LIM domain containing 1 |
| RBMS2 | 1.6 | RNA binding motif, single stranded interacting protein 2 |
| SLC3A2 | 1.6 | solute carrier family 3 (activators of dibasic and neutral amino acid transport), member 2 |
| HSPBAP1 | 1.6 | HSPB (heat shock 27kDa) associated protein 1 |
| FES | 1.6 | feline sarcoma oncogene |
| IFI6 | 1.6 | interferon, alpha-inducible protein 6 |
| MID1 | 1.6 | midline 1 (Opitz/BBB syndrome) |
| BMP4 | 1.6 | bone morphogenetic protein 4 |
| CKS2 | 1.6 | CDC28 protein kinase regulatory subunit 2 |
| HIF1A | 1.6 | histone cluster 1, H1c |
| MRCL3 | 1.6 | myosin regulatory light chain MRCL3 |
| OCLN | 1.6 | occludin |
| SNAPC1 | 1.6 | small nuclear RNA activating complex, polypeptide 1, 43kDa |
| CD59 | 1.6 | CD59 molecule, complement regulatory protein |
| PKIB | 1.6 | protein kinase (cAMP-dependent, catalytic) inhibitor beta |
| ZNF585A | 1.6 | zinc finger protein 578A |
| ACTN4 | 1.6 | actinin, alpha 4 |
| APOL2 | 1.6 | apolipoprotein L, 2 |
| MAP1B | 1.6 | microtubule-associated protein 1B |
| PSMG4 | 1.6 | proteasome (prosome, macropain) assembly chaperone 4 |
| IER5 | 1.6 | immediate early response 5 |
| AIFM2 | 1.6 | absent in melanoma 1 |
| STX3 | 1.6 | syntaxin 3 |
| RAP2B | 1.6 | RAP2B, member of RAS oncogene family |
| CACNB3 | 1.6 | calneuron 1 |
| CXCL16 | 1.6 | chemokine (C-X-C motif) ligand 16 |
| TPPP | 1.6 | tubulin polymerization promoting protein |
| RHBDL3 | 1.6 | rhomboid 5 homolog 3 (Drosophila) |
| RUSC2 | 1.6 | RUN and SH3 domain containing 2 |
| PFTK1 | 1.6 | PFTAIRE protein kinase 1 |
| CDC2L6 | 1.6 | cell division cycle 2-like 6 (CDK8-like) |
| SEC14L2 | 1.6 | SEC14-like 2 (S. cerevisiae) |
| MAST4 | 1.6 | microtubule associated serine/threonine kinase family member 4 |
| WIPF3 | 1.6 | WAS/WASL interacting protein family, member 3 |
| FHOD3 | 1.6 | formin homology 2 domain containing 3 |
| SCARF2 | 1.5 | scavenger receptor class F, member 2 |
| DCDC2 | 1.5 | doublecortin domain containing 2 |
| SQSTM1 | 1.5 | sequestosome 1 |
| MACROD2 | 1.5 | MACRO domain containing 2 |
| APBB2 | 1.5 | amyloid beta (A4) precursor protein-binding, family B, member 2 |
| ISG15 | 1.5 | ISG15 ubiquitin-like modifier |
| TMC7 | 1.5 | transmembrane channel-like 7 |
| PTPRM | 1.5 | protein tyrosine phosphatase, receptor type, M |
| RGNEF | 1.5 | Rho-guanine nucleotide exchange factor |
| OVGP1 | 1.5 | oviductal glycoprotein 1, 120kDa |
| GPX8 | 1.5 | glutathione peroxidase 8 (putative) |
| SERAC1 | 1.5 | serine active site containing 1 |
| ZNF69 | 1.5 | zinc finger protein 69 |
| C17orf63 | 1.5 | chromosome 17 open reading frame 63 |
| MYH9 | 1.5 | myosin, heavy chain 9, non-muscle |
| GABRA2 | 1.5 | gamma-aminobutyric acid (GABA) A receptor, alpha 2 |
| SEC31B | 1.5 | SEC31 homolog B (S. cerevisiae) |
| BTG2 | 1.5 | BTG family, member 2 |
| EPB41L1 | 1.5 | erythrocyte membrane protein band 4.1-like 1 |
| SULT1C4 | 1.5 | sulfotransferase family, cytosolic, 1C, member 4 |
| RNF146 | 1.5 | ring finger protein 146 |
| HEPH | 1.5 | hephaestin |
| RND1 | 1.5 | Rho family GTPase 1 |
| PITPNM2 | 1.5 | phosphatidylinositol transfer protein, membrane-associated 2 |
| HCCA2 | 1.5 | HCCA2 protein |
| HCN3 | 1.5 | hyperpolarization activated cyclic nucleotide-gated potassium channel 3 |
| NKIRAS1 | 1.5 | NFKB inhibitor interacting Ras-like 1 |
| FHIT | 1.5 | fragile histidine triad gene |
| BHLHB9 | 1.5 | basic helix-loop-helix domain containing, class B, 9 |
| ANXA2 | 1.5 | annexin A2 |
| AHR | 1.5 | aryl hydrocarbon receptor |
| DUSP10 | 1.5 | dual specificity phosphatase 10 |
| BCL3 | 1.5 | B-cell CLL/lymphoma 3 |
| TPM4 | 1.5 | tropomyosin 4 |
| INSR | 1.5 | insulin receptor |
| MAFG | 1.5 | v-maf musculoaponeurotic fibrosarcoma oncogene homolog G (avian) |
| FAM129A | 1.5 | family with sequence similarity 129, member A |
| RCAN2 | 1.5 | regulator of calcineurin 2 |
| C11orf67 | 1.5 | chromosome 11 open reading frame 67 |
| CTTNBP2 | 1.5 | cortactin binding protein 2 |
| DDR1 | 1.5 | discoidin domain receptor tyrosine kinase 1 |
| MAPRE3 | 1.5 | microtubule-associated protein, RP/EB family, member 3 |
| ABHD4 | 1.5 | abhydrolase domain containing 4 |
| ADFP | 1.5 | adipose differentiation-related protein |
| C1orf216 | 1.5 | chromosome 1 open reading frame 216 |
| PRICKLE2 | 1.5 | prickle homolog 2 (Drosophila) |
| UHRF2 | 1.5 | ubiquitin-like with PHD and ring finger domains 2 |
| TP53I11 | 1.5 | tumor protein p53 inducible protein 11 |
| STARD13 | 1.5 | StAR-related lipid transfer (START) domain containing 13 |
| COL12A1 | 1.5 | collagen, type XVI, alpha 1 |
| CACNA1G | 1.5 | calcium channel, voltage-dependent, gamma subunit 4 |
| PGCP | 1.5 | plasma glutamate carboxypeptidase |
| DISC1 | 1.5 | disrupted in schizophrenia 1 |
| FBXW4 | 1.5 | F-box and WD repeat domain containing 4 |
| CABLES1 | 1.5 | calcium channel, voltage-dependent, L type, alpha 1F subunit |
| PDE1A | 1.5 | phosphodiesterase 1A, calmodulin-dependent |
| TUBB6 | 1.5 | tubulin, beta 6 |
| ACTG1 | 1.5 | actin, gamma 1 |
| PLA1A | 1.5 | phospholipase A1 member A |
| MOBKL2B | 1.5 | MOB1, Mps One Binder kinase activator-like 2B (yeast) |
| PALLD | 1.5 | palladin, cytoskeletal associated protein |
| SLC20A2 | 1.5 | solute carrier family 20 (phosphate transporter), member 2 |
| GLP1R | 1.5 | GLI pathogenesis-related 1 |
| SOD3 | 1.5 | superoxide dismutase 3, extracellular |
| JMJD3 | 1.5 | lysine (K)-specific demethylase 6B |
| MAP3K12 | 1.5 | mitogen-activated protein kinase kinase kinase 12 |
| C10orf6 | 1.5 | chromosome 10 open reading frame 6 |
| MRLC2 | 1.5 | myosin regulatory light chain MRCL2 |
| BMF | 1.5 | Bcl2 modifying factor |
| APOL6 | 1.5 | apolipoprotein L, 6 |
| PAQR5 | 1.5 | progestin and adipoQ receptor family member V |
| TEAD1 | 1.5 | TEA domain family member 1 (SV40 transcriptional enhancer factor) |
| NUAK1 | 1.5 | NUAK family, SNF1-like kinase, 1 |
| PARVA | 1.5 | parvin, alpha |
| QPCT | 1.5 | glutaminyl-peptide cyclotransferase |
| LOC253012 | 1.5 | LOC253012 |
| ABCC3 | 1.5 | ATP-binding cassette, sub-family C (CFTR/MRP), member 3 |
| CCND1 | 1.5 | cyclin D1 |
| KCNC4 | 1.5 | potassium voltage-gated channel, Shaw-related subfamily, member 4 |
| SPTBN1 | 1.5 | spectrin, beta, non-erythrocytic 1 |
| PHLPPL | 1.5 | PH domain and leucine rich repeat protein phosphatase 2 |
| B2M | 1.5 | beta-2-microglobulin |
| TAP2 | 1.5 | transporter 2, ATP-binding cassette, sub-family B (MDR/TAP) |
| CPEB4 | 1.5 | cytoplasmic polyadenylation element binding protein 4 |
| FBXL18 | 1.5 | F-box and leucine-rich repeat protein 18 |
| TMEFF1 | 1.5 | transmembrane protein with EGF-like and two follistatin-like domains 1 |
| LMNA | 1.5 | lamin A/C |
| RHOU | 1.5 | ras homolog gene family, member U |
| APC2 | 1.5 | adenomatosis polyposis coli 2 |
| B3GALTL | 1.5 | beta 1,3-galactosyltransferase-like |
| MCOLN2 | 1.5 | mucolipin 2 |
| NPDC1 | 1.5 | neural proliferation, differentiation and control, 1 |
| RASA4 | 1.5 | RAS p21 protein activator 4 |
| WNT5B | 1.5 | wingless-type MMTV integration site family, member 5B |
| RIC8B | 1.5 | resistance to inhibitors of cholinesterase 8 homolog B (C. elegans) |
| SULT1C2 | 1.5 | sulfotransferase family, cytosolic, 1C, member 2 |
| PSCD3 | 1.5 | cytohesin 3 |
| MPDZ | 1.5 | multiple PDZ domain protein |
| TP53INP1 | 1.5 | tumor protein p53 inducible nuclear protein 1 |
| DAGLA | 1.5 | diacylglycerol lipase, alpha |
| C4orf32 | 1.5 | chromosome 4 open reading frame 32 |
| C9orf150 | 1.5 | chromosome 9 open reading frame 150 |
| LYZ | -1.5 | lysozyme (renal amyloidosis) |
| NPTX2 | -1.5 | neuronal pentraxin II |
| BCL7C | -1.5 | B-cell CLL/lymphoma 7C |
| ODZ2 | -1.5 | odz, odd Oz/ten-m homolog 2 (Drosophila) |
| ASB9 | -1.5 | ankyrin repeat and SOCS box-containing 9 |
| POLR3G | -1.5 | polymerase (RNA) III (DNA directed) polypeptide G (32kD) |
| APOA1 | -1.5 | apolipoprotein A-I |
| NPW | -1.5 | neuropeptide W |
| IGSF1 | -1.5 | immunoglobulin superfamily, member 1 |
| PXMP4 | -1.5 | peroxisomal membrane protein 4, 24kDa |
| PMFBP1 | -1.5 | polyamine modulated factor 1 binding protein 1 |
| VASN | -1.5 | vasorin |
| ZNF18 | -1.5 | zinc finger protein 18 |
| MDP-1 | -1.5 | magnesium-dependent phosphatase 1 |
| ANP32E | -1.5 | acidic (leucine-rich) nuclear phosphoprotein 32 family, member E |
| CCR6 | -1.5 | chemokine (C-C motif) receptor 6 |
| AHSG | -1.5 | apoptosis-inducing factor, mitochondrion-associated, 2 |
| SYNE2 | -1.5 | spectrin repeat containing, nuclear envelope 2 |
| PDK1 | -1.5 | pyruvate dehydrogenase kinase, isozyme 1 |
| TMEM129 | -1.5 | transmembrane protein 129 |
| C4BPA | -1.5 | complement component 4 binding protein, alpha |
| DUSP19 | -1.5 | dual specificity phosphatase 19 |
| NUF2 | -1.5 | NUF2, NDC80 kinetochore complex component, homolog (S. cerevisiae) |
| NUF2 | -1.5 | NUF2, NDC80 kinetochore complex component, homolog (S. cerevisiae) |
| NUF2 | -1.5 | NUF2, NDC80 kinetochore complex component, homolog (S. cerevisiae) |
| MRPL54 | -1.5 | mitochondrial ribosomal protein L54 |
| HSPA5 | -1.5 | heat shock 70kDa protein 5 (glucose-regulated protein, 78kDa) |
| KCNE3 | -1.5 | potassium voltage-gated channel, Isk-related family, member 3 |
| BCKDHB | -1.5 | branched chain keto acid dehydrogenase E1, beta polypeptide |
| TMEM86B | -1.5 | transmembrane protein 86B |
| ACTN3 | -1.5 | actinin, alpha 3 |
| AGR2 | -1.5 | anterior gradient homolog 2 |
| H2AFX | -1.5 | H2A histone family, member X |
| MSTO1 | -1.5 | misato homolog 1 |
| METRN | -1.5 | meteorin, glial cell differentiation regulator |
| KAZALD1 | -1.6 | Kazal-type serine peptidase inhibitor domain 1 |
| ZNF117 | -1.6 | zinc finger protein 117 |
| TMEM80 | -1.6 | transmembrane protein 80 |
| MYCN | -1.6 | v-myc myelocytomatosis viral related oncogene, neuroblastoma derived (avian) |
| ZNF101 | -1.6 | zinc finger protein 101 |
| ROS1 | -1.6 | c-ros oncogene 1 , receptor tyrosine kinase |
| TCTA | -1.6 | T-cell leukemia translocation altered gene |
| SPHAR | -1.6 | S-phase response (cyclin related) |
| SYNC1 | -1.6 | syncoilin, intermediate filament protein 1 |
| SERHL | -1.6 | serine hydrolase-like |
| GK | -1.6 | glycerol kinase |
| KIAA1024 | -1.6 | KIAA1024 |
| AGXT | -1.7 | alanine-glyoxylate aminotransferase 2 |
| PANX2 | -1.7 | pannexin 2 |
| DHFR | -1.7 | dihydrofolate reductase |
| METTL7A | -1.7 | major facilitator superfamily domain containing 2 |
| GPNMB | -1.7 | glycoprotein (transmembrane) nmb |
| COQ7 | -1.7 | coenzyme Q7 homolog, ubiquinone |
| MAT1A | -1.7 | methionine adenosyltransferase I, alpha |
| PDGFRL | -1.7 | platelet-derived growth factor receptor-like |
| SARS2 | -1.7 | seryl-tRNA synthetase 2, mitochondrial |
| RBM3 | -1.7 | RNA binding motif (RNP1, RRM) protein 3 |
| PPP1R14B | -1.8 | protein phosphatase 1, regulatory (inhibitor) subunit 14B |
| SFXN2 | -1.8 | sideroflexin 2 |
| SLC6A4 | -1.8 | solute carrier family 6 (neurotransmitter transporter, serotonin), member 4 |
| RBP2 | -1.8 | retinol binding protein 2, cellular |
| ARG1 | -1.8 | arginase, liver |
| FASN | -1.8 | fatty acid synthase |
| HMGCS2 | -1.8 | 3-hydroxy-3-methylglutaryl-Coenzyme A synthase 2 |
| SLC37A4 | -1.8 | solute carrier family 37 (glucose-6-phosphate transporter), member 4 |
| C8A | -1.8 | complement component 8, alpha polypeptide |
| AGXT2 | -1.9 | alanine-glyoxylate aminotransferase 2 |
| SLCO1B3 | -1.9 | solute carrier organic anion transporter family, member 1B3 |
| C10orf125 | -1.9 | chromosome 10 open reading frame 125 |
| GUCA1B | -1.9 | guanylate cyclase activator 1B (retina) |
| TMEM82 | -1.9 | transmembrane protein 82 |
| SERPINC1 | -1.9 | serpin peptidase inhibitor, clade C (antithrombin), member 1 |
| APOM | -2.0 | apolipoprotein M |
| VPS37D | -2.0 | vacuolar protein sorting 37 homolog D (S. cerevisiae) |
| ADH6 | -2.0 | alcohol dehydrogenase 6 (class V) |
| GSTA2 | -2.0 | glutathione S-transferase alpha 2 |
| C19orf39 | -2.0 | chromosome 19 open reading frame 39 |
| KRT222P | -2.0 | keratin 222 pseudogene |
| DLK1 | -2.0 | delta-like 1 homolog (Drosophila) |
| NQO1 | -2.0 | NAD(P)H dehydrogenase, quinone 1 |
| ACSM3 | -2.0 | acyl-CoA synthetase medium-chain family member 3 |
| COL8A2 | -2.0 | collagen, type VIII, alpha 2 |
| G6PC | -2.0 | glucose-6-phosphatase, catalytic subunit |
| CACNA1A | -2.0 | calcium channel, voltage-dependent, T type, alpha 1G subunit |
| ACSM2B | -2.0 | Acyl-coenzyme A synthetase |
| CHD5 | -2.1 | chromodomain helicase DNA binding protein 5 |
| OPN1SW | -2.1 | opsin 1 (cone pigments), short-wave-sensitive |
| HIST1H2BH | -2.1 | histone cluster 2, H2ac |
| DYRK3 | -2.1 | dual-specificity tyrosine-(Y)-phosphorylation regulated kinase 3 |
| ESPNL | -2.1 | espin-like |
| ZDHHC19 | -2.1 | zinc finger, DHHC-type containing 19 |
| FTCD | -2.1 | formiminotransferase cyclodeaminase |
| DNAH10 | -2.1 | dynein, axonemal, heavy chain 10 |
| TGM3 | -2.2 | transglutaminase 3 (E polypeptide, protein-glutamine-gamma-glutamyltransferase) |
| ZAN | -2.2 | zonadhesin |
| HIST1H1C | -2.2 | histone cluster 1, H1c |
| RP1L1 | -2.2 | retinitis pigmentosa 1-like 1 |
| C2orf54 | -2.2 | chromosome 2 open reading frame 54 |
| ALPI | -2.2 | alkaline phosphatase, intestinal |
| PDE6A | -2.2 | phosphodiesterase 6A, cGMP-specific, rod, alpha |
| PDE6A | -2.2 | phosphodiesterase 6A, cGMP-specific, rod, alpha |
| A1BG | -2.2 | alpha-1-B glycoprotein |
| LHFPL4 | -2.2 | lipoma HMGIC fusion partner-like 4 |
| FOSB | -2.2 | FBJ murine osteosarcoma viral oncogene homolog B |
| CLCN1 | -2.2 | chloride channel 1, skeletal muscle |
| CSF3R | -2.3 | colony stimulating factor 3 receptor (granulocyte) |
| APOA5 | -2.4 | apolipoprotein A-V |
| SRCRB4D | -2.5 | scavenger receptor cysteine rich domain containing, group B (4 domains) |
| TBX21 | -2.5 | T-box 21 |
| CPS1 | -2.6 | carbamoyl-phosphate synthetase 1 |
| HFE2 | -2.6 | hemochromatosis type 2 |
| PHYHD1 | -2.6 | phytanoyl-CoA dioxygenase domain containing 1 |
| AKR1C3 | -2.6 | aldo-keto reductase family 1, member C3 |
| C1orf186 | -2.7 | chromosome 19 open reading frame 86 |
| TPRG1 | -2.7 | tumor protein p63 regulated 1 |
| OSTalpha | -2.8 | organic solute transporter alpha |
| CORIN | -2.9 | corin, serine peptidase |
| CUX2 | -3.0 | cut-like homeobox 2 |
| GSTA1 | -3.1 | glutathione S-transferase alpha 1 |
| FOS | -3.1 | v-fos FBJ murine osteosarcoma viral oncogene homolog |
| COL17A1 | -3.2 | collagen, type V, alpha 3 |
| COL17A1 | -3.2 | collagen, type V, alpha 3 |
| EGR1 | -3.3 | early growth response 1 |
| IL21R | -3.8 | interleukin 21 receptor |
| IL21R | -3.8 | interleukin 21 receptor |

Proteomics Data

| **Spot** | **Protein Name** | **SwissProt Accession number** | **Fold change** | **Estimated From 2 D gel** | | **Calculated** | | **Score** | **Peptides matched** |
| --- | --- | --- | --- | --- | --- | --- | --- | --- | --- |
| **number** |  |  |  | **Mass kDa** | **pI** | **Mass Da** | **pI** |  |  |
| 1 | Vinculin | P18206 | U 1.94 | 73.1 | 6.09 | 124292 | 5.5 | 259 | 9 (8) |
| 2 | Matrin-3 | P43243 | D -2.46 | 72.3 | 5.75 | 95078 | 5.87 | 147 | 7 |
|  | ATP-citrate synthase | P53396 | D* | 72.0 | 4.28 | 121674 | 6.95 | 82 | 2 |
| 3 | Matrin-3 | P43243 |  |  |  | 95078 | 5.87 | 53 | 1 |
|  | Splicing factor 3 subunit 1 | Q15459 | D* | 71.9 | 5.35 | 88888 | 5.15 | 155 | 6 |
| 4 | Matrin-3 | P43243 | D* | 71.8 | 5.35 | 95078 | 5.87 | 72 | 2 |
| 5 | Caprin-1 | Q14444 | U 2.14 | 70.1 | 5.29 | 78489 | 5.14 | 132 | 3 |
|  | Alpha-actinin-4 | O43707 |  |  |  | 105245 | 5.27 | 107 | 3 |
|  | Heat shock protein 105 kDa | Q92598 |  |  |  | 97716 | 5.28 | 75 | 5 (3) |
| 6 | Ubiquitin carboxyl-terminal hydrolase 5 | P45974 | D -2.12 | 69.20 | 5.05 | 96638 | 4.91 | 416 | 12 |
|  | Nucleolin | P19338 |  |  |  | 76625 | 4.6 | 218 | 5 |
|  | Heat shock protein HSP 90-alpha | P07900 |  |  |  | 85006 | 4.94 | 154 | 4 |
|  | Heat shock protein HSP 90-beta | P08238 |  |  |  | 83554 | 4.97 | 142 | 4 (0) |
| 7 | Procollagen-lysine,2-oxoglutarate 5-dioxygenase 3 | O60568 | D* | 65.00 | 5.93 | 85302 | 5.69 | 153 | 6 (5) |
| 8 | 78 kDa glucose-regulated protein | P11021 | D -2.77 | 64 | 5.33 | 72402 | 5.07 | 102 | 4 (3) |
|  | Acylamino-acid-releasing enzyme | P13798 |  |  |  | 82142 | 5.29 | 150 | 6 (5) |
|  | Keratin, type II cytoskeletal 1 | P04264 |  |  |  | 66149 | 8.16 | 78 | 1 |
| 9 | Ezrin | P15311 | U 1.56 | 63.6 | 6.27 | 69484 | 5.94 | 459 | 14 (11) |
|  | Rootletin | Q5TZA2 |  |  |  | 228787 | 5.45 | 46 | 1 (0) |
|  | Early endosome antigen 1 | Q15075 |  |  |  | 163337 | 5.53 | 44 | 2 (0) |
|  | Radixin | P35241 |  |  |  | 68635 | 6.03 | 217 | 6 (0) |
|  | Keratin, type II cytoskeletal 1 | P04264 |  |  |  | 66149 | 8.16 | 44 | 1 |
| 10 | Protein phosphatase 1G | O15355 | D * | 62.3 | 4.47 | 59919 | 4.28 | 183 | 6 |
| 11 | Heat shock cognate 71 kDa protein | P11142 | D* | 61.2 | 5.14 | 71082 | 5.37 | 278 | 9 |
|  | Heat shock-related 70 kDa protein 2 | P54652 |  |  |  | 70263 | 5.56 | 168 | 6 (0) |
|  | ATP-dependent DNA helicase 2 subunit | P12956 |  |  |  | 70084 | 6.23 | 94 | 4 (3) |
|  | Sorting nexin-2 | O60749 |  |  |  | 58549 | 5.04 | 83 | 1 |
| 12 | Heat shock cognate 71 kDa protein | P11142 | D* | 61.2 | 5.21 | 71082 | 5.37 | 251 | 10 |
|  | Heat shock-related 70 kDa protein 2 | P54652 |  |  |  | 70263 | 5.56 | 73 | 4 (0) |
|  | Sorting nexin-2 | O60749 |  |  |  | 58549 | 5.04 | 60 | 2 (1) |
|  | ATP-dependent DNA helicase 2 subunit 1 | P12956 |  |  |  | 70084 | 6.23 | 56 | 1 |
| 13 | Alpha-fetoprotein | P02771 | D* | 59.9 | 5.67 | 70458 | 5.48 | 256 | 10 (9) |
|  | Coronin-1B | Q9BR76 |  |  |  | 54885 | 5.6 | 140 | 6 |
| 14 | Stress-70 protein, mitochondrial | P38646 | D -2.35 | 59.8 | 5.5 | 73920 | 5.87 | 501 | 17 (12) |
|  | Annexin A6 | P08133 |  |  |  | 76168 | 5.42 | 233 | 7 |
|  | Heat shock 70 kDa protein 1 | P08107 |  |  |  | 70294 | 5.48 | 76 | 2 |
|  | Insulin-like growth factor 2 mRNA-binding protein 1 | Q9NZI8 |  |  |  | 63759 | 9.26 | 47 | 2 |
|  | Plastin-3 | P13797 |  |  |  | 70904 | 5.52 | 36 | 2 (1) |
|  | Dihydrolipoyllysine-residue acetyltransferase component of pyruvate dehydrogenase complex, mitochondrial | P10515 |  |  |  | 69466 | 7.96 | 175 | 7 |
|  | Keratin, type II cytoskeletal 5 | P13647 |  |  |  | 62568 | 7.58 | 78 | 1 |
| 14 | Keratin, type II cytoskeletal 6A | P02538 | D -2.35 | 59.8 | 5.5 | 60293 | 8.09 | 78 | 1 (0) |
|  | Keratin, type II cytoskeletal 6B | P04259 |  |  |  | 60274 | 8.09 | 78 | 1 (0) |
|  | Keratin, type II cytoskeletal 6C | P48668 |  |  |  | 60273 | 8.09 | 78 | 1 (0) |
|  | Keratin, type II cytoskeletal 75 | O95678 |  |  |  | 59753 | 7.6 | 78 | 1 (0) |
|  | Keratin, type II cytoskeletal 79 | Q5XKE5 |  |  |  | 58059 | 6.75 | 78 | 1 (0) |
|  | Keratin, type II cytoskeletal 2 epidermal | P35908 |  |  |  | 66111 | 8.07 | 78 | 1 (0) |
| 15 | Lamin-B1 | P20700 | D* | 62.2 | 5.21 | 66653 | 5.11 | 587 | 18 (16) |
|  | Insulin-like growth factor 2 mRNA-binding protein 1 | Q9NZI8 |  |  |  | 63759 | 9.26 | 94 | 4 (2) |
|  | Insulin-like growth factor 2 mRNA-binding protein 2 | Q9Y6M1 |  |  |  | 66195 | 8.48 | 58 | 2 (1) |
|  | Insulin-like growth factor 2 mRNA-binding protein 3 | O00425 |  |  |  | 64023 | 8.99 | 58 | 2 (0) |
|  | Leucine-rich repeat-containing protein 47 | Q8N1G4 |  |  |  | 64004 | 8.55 | 34 | 2 (1) |
| 16 | Lamin-B2 | Q03252 | U* | 60 | 5.44 | 67762 | 5.29 | 305 | 7 |
|  | Ras GTPase-activating protein-binding protein 1 | Q13283 |  |  |  | 52189 | 5.36 | 184 | 8 |
|  | Anaphase-promoting complex subunit 7 | Q9UJX3 |  |  |  | 63720 | 5.5 | 90 | 2 |
|  | Heterogeneous nuclear ribonucleoprotein K | P61978 |  |  |  | 51230 | 5.39 | 86 | 2 |
|  | Insulin-like growth factor 2 mRNA-binding protein 1 | Q9NZI8 |  |  |  | 63759 | 9.26 | 43 | 2 |
| 17 | Heterogeneous nuclear ribonucleoprotein K | P61978 | U 2.04 | 58.7 | 5.33 | 51230 | 5.39 | 268 | 8 (7) |
|  | Poly(U)-binding-splicing factor PUF60 | Q9UHX1 |  |  |  | 60009 | 5.19 | 121 | 4 (3) |
|  | 60 kDa heat shock protein, mitochondrial | P10809 |  |  |  | 61187 | 5.7 | 92 | 5 (4) |
|  | Keratin, type II cytoskeletal 1 | P04264 |  |  |  | 66149 | 8.16 | 62 | 1 |
|  | Stress-70 protein, mitochondrial | P38646 |  |  |  | 73920 | 5.87 | 57 | 5 (3) |
|  | Beta-galactosidase | P16278 |  |  |  | 76483 | 6.1 | 43 | 2 |
|  | Bifunctional purine biosynthesis protein PURH | P31939 |  |  |  | 65089 | 6.27 | 86 | 2 |
|  | Eukaryotic initiation factor 4A-I | P60842 |  |  |  | 46353 | 5.32 | 75 | 1 |
|  | Eukaryotic initiation factor 4A-II | Q14240 |  |  |  | 46601 | 5.33 | 74 | 1 (0) |
|  | Polypyrimidine tract-binding protein 1 | P26599 |  |  |  | 57357 | 9.22 | 35 | 1 |
| 18 | Pyruvate kinase isozymes M1/M2 | P14618 | U 1.53 | 57.6 | 6.08 | 58470 | 7.96 | 82 | 4 |
|  | UDP-N-acetylhexosamine pyrophosphorylase | Q16222 |  |  |  | 59131 | 5.92 | 38 | 1 |
|  | Coatomer subunit delta | P48444 |  |  |  | 57630 | 5.89 | 71 | 1 |
|  | Keratin, type II cytoskeletal 1 | P04264 |  |  |  | 66149 | 8.16 | 59 | 1 |
|  | UDP-N-acetylhexosamine pyrophosphorylase | Q16222 |  |  |  | 59131 | 5.92 | 38 | 2 |
| 19 | T-complex protein 1 subunit theta | P50990 | U 2 | 57.4 | 5.44 | 60153 | 5.42 | 367 | 11 (10) |
|  | 60 kDa heat shock protein, mitochondrial | P10809 |  |  |  | 61187 | 5.7 | 288 | 12 (10) |
|  | T-complex protein 1 subunit epsilon | P48643 |  |  |  | 60089 | 5.45 | 192 | 7 |
|  | Splicing factor 3A subunit 3 | Q12874 |  |  |  | 59154 | 5.27 | 77 | 1 |
|  | Fatty acid synthase | P49327 |  |  |  | 275858 | 5.99 | 71 | 2 |
|  | Heterogeneous nuclear ribonucleoprotein K | P61978 |  |  |  | 51230 | 5.39 | 57 | 3 |
|  | Splicing factor 3A subunit 3 | Q12874 |  |  |  | 59154 | 5.27 | 59 | 2 |
|  | Keratin, type II cytoskeletal 1 | P04264 |  |  |  | 66149 | 8.16 | 51 | 1 |
| 20 | T-complex protein 1 subunit theta | P50990 | D* | 57.1 | 4.18 | 60153 | 5.42 | 134 | 3 |
|  | Zinc finger CCCH domain-containing protein 15 | Q8WU90 |  |  |  | 48972 | 5.22 | 77 | 1 |
| 20 | 60 kDa heat shock protein, mitochondrial | P10809 | D* | 57.1 | 4.18 | 61187 | 5.7 | 39 | 1 |
| 21 | Nucleosome assembly protein 1-like 1 | P55209 | D* | 56.1 | 4.11 | 45631 | 4.36 | 172 | 4 |
| 22 | V-type proton ATPase subunit B, brain isoform | P21281 | D -2.17 | 55.6 | 5.68 | 56807 | 5.57 | 638 | 21 (18) |
|  | Protein disulfide-isomerase A3 | P30101 |  |  |  | 57146 | 5.98 | 428 | 13 (12) |
| 23 | Methylcrotonoyl-CoA carboxylase beta chain, mitochondrial | Q9HCC0 | U* | 56.5 | 6.89 | 61808 | 7.58 | 289 | 8 (7) |
|  | Tyrosyl-tRNA synthetase, cytoplasmic | P54577 |  |  |  | 59448 | 6.61 | 41 | 1 |
| 24 | Adenylyl cyclase-associated protein 1 | Q01518 | U 1.99 | 55.2 | 6.98 | 52222 | 8.27 | 163 | 6 |
|  | T-complex protein 1 subunit delta | P50991 |  |  |  | 58401 | 7.96 | 122 | 3 |
|  | T-complex protein 1 subunit eta | Q99832 |  |  |  | 59842 | 7.55 | 87 | 5 (4) |
|  | Dihydrolipoyl dehydrogenase, mitochondrial | P09622 |  |  |  | 54686 | 7.59 | 79 | 1 |
| 25 | Protein disulfide-isomerase A3 | P30101 | U 1.99 | 55.2 | 5.67 | 57146 | 5.98 | 618 | 18 (15) |
|  | Retinal dehydrogenase 1 | P00352 |  |  |  | 55454 | 6.3 | 103 | 3 |
|  | Eukaryotic translation initiation factor 5 | P55010 |  |  |  | 49648 | 5.41 | 33 | 1 |
| 26 | Probable Xaa-Pro aminopeptidase 3 | Q9NQH7 | D* | 54.6 | 6.26 | 57624 | 6.37 | 66 | 2 |
| 27 | Retinal dehydrogenase 1 | P00352 | U 2.55 | 53.8 | 6.31 | 55454 | 6.3 | 735 | 20 (17) |
|  | Mitochondrial-processing peptidase subunit alpha | Q10713 |  |  |  | 58729 | 6.45 | 52 | 1 |
|  | Cytosol aminopeptidase | P28838 |  |  |  | 56530 | 8.03 | 57 | 2 |
|  | Dynein heavy chain 11, axonemal | Q96DT5 |  |  |  | 524843 | 6.03 | 39 | 4 (1) |
| 28 | Polyadenylate-binding protein 2 | Q86U42 | D* | 21.8 | 4.37 | 32843 | 5.04 | 173 | 3 |
|  | Uncharacterized protein C6orf174 | Q5TF21 |  |  |  | 103478 | 5.81 | 40 | 1 (0) |
| 29 | Tetratricopeptide repeat protein 38 | Q5R3I4 | D* | 51 | 5.8 | 53267 | 5.61 | 199 | 7 (6) |
|  | Elongation factor 1-alpha 1 | P68104 |  |  |  | 50451 | 9.1 | 113 | 3 |
|  | Putative elongation factor 1-alpha-like 3 | Q5VTE0 |  |  |  | 50495 | 9.15 | 113 | 3 (0) |
| 30 | Seryl-tRNA synthetase, mitochondrial | Q9NP81 | U 1.81 | 50.7 | 6.88 | 58702 | 8.35 | 172 | 6 (5) |
|  | Elongation factor 1-alpha 1 | P68104 |  |  |  | 50451 | 9.1 | 127 | 4 |
|  | Putative elongation factor 1-alpha-like 3 | Q5VTE0 |  |  |  | 50495 | 9.15 | 127 | 4 (0) |
|  | Septin-10 | Q9P0V9 |  |  |  | 53016 | 6.35 | 121 | 5 (3) |
|  | Glutamate dehydrogenase 1, mitochondrial | P00367 |  |  |  | 61701 | 7.66 | 75 | 2 |
|  | Fascin | Q16658 |  |  |  | 55123 | 6.84 | 56 | 2 |
|  | ATP synthase subunit alpha, mitochondrial | P25705 |  |  |  | 59828 | 9.16 | 35 | 1 |
|  | Keratin, type II cytoskeletal 1 | P04264 |  |  |  | 66149 | 8.16 | 74 | 1 |
| 31 | Keratin, type II cytoskeletal 8 | P05787 | D* | 50.8 | 5.48 | 53671 | 5.52 | 204 | 3 |
|  | Coiled-coil domain-containing protein 110 | Q8TBZ0 |  |  |  | 97235 | 5.88 | 49 | 1 (0) |
|  | Elongation factor 1-alpha 1 | P68104 |  |  |  | 50451 | 9.1 | 46 | 1 |
|  | Elongation factor 1-alpha 2 | Q05639 |  |  |  | 50780 | 9.11 | 46 | 1 (0) |
|  | Putative elongation factor 1-alpha-like 3 | Q5VTE0 |  |  |  | 50495 | 9.15 | 46 | 1 (0) |
|  | RuvB-like 2 | Q9Y230 |  |  |  | 51296 | 5.49 | 41 | 1 |
| 32 | SSB | P05455 | U 1.78 | 50.7 | 6.25 | 46979 | 6.68 | 163 | 3 |
|  | Elongation factor 1-alpha 1 | P68104 |  |  |  | 50451 | 9.1 | 92 | 3 |
|  | Putative elongation factor 1-alpha-like 3 | Q5VTE0 |  |  |  | 50495 | 9.15 | 92 | 3 (0) |
| 32 | Alpha-enolase | P06733 | U 1.78 | 50.7 | 6.25 | 47481 | 7.01 | 61 | 2 (1) |
|  | Phenylalanine-4-hydroxylase | P00439 |  |  |  | 52343 | 6.15 | 51 | 1 |
|  | Keratin, type II cytoskeletal 1 | P04264 |  |  |  | 66149 | 8.16 | 52 | 1 |
| 33 | Elongation factor 1-alpha 1 | P68104 | D* | 50.4 | 5.9 | 50451 | 9.1 | 61 | 2 |
|  | Putative elongation factor 1-alpha-like 3 | Q5VTE0 |  |  |  | 50495 | 9.15 | 61 | 2 (0) |
| 34 | Regulator of chromosome condensation | P18754 | D* | 48.3 | 4.83 | 45397 | 7.18 | 301 | 7 |
|  | COBW domain-containing protein 1 | Q9BRT8 |  |  |  | 44383 | 4.76 | 213 | 3 |
|  | COBW domain-containing protein 2 | Q8IUF1 |  |  |  | 44349 | 4.79 | 213 | 3 (0) |
|  | Elongation factor 1-gamma | P26641 |  |  |  | 50429 | 6.25 | 66 | 2 |
|  | Heat shock protein HSP 90-alpha | P07900 |  |  |  | 85006 | 4.94 | 65 | 2 |
|  | Heat shock protein HSP 90-beta | P08238 |  |  |  | 83554 | 4.97 | 65 | 3 (0) |
| 35 | 60 kDa heat shock protein, mitochondrial | P10809 | D* | 47.7 | 5.83 | 61187 | 5.7 | 133 | 4 (3) |
|  | 26S protease regulatory subunit 7 | P35998 |  |  |  | 49002 | 5.71 | 101 | 4 |
| 36 | Annexin A7 | P20073 | D* | 56.8 | 5.76 | 52991 | 5.52 | 126 | 3 |
| 37 | Rab GDP dissociation inhibitor beta | P50395 | D* | 46.9 | 6.33 | 51087 | 6.11 | 513 | 21 (18) |
|  | Adenylosuccinate synthetase isozyme 2 | P30520 |  |  |  | 50465 | 6.13 | 160 | 4 |
| 38 | Tubulin alpha-1A chain | Q71U36 | D -2.03 | 46 | 6.02 | 50788 | 4.94 | 129 | 5 |
|  | Tubulin alpha-1B chain | P68363 |  |  |  | 50804 | 4.94 | 129 | 5 (0) |
|  | Actin, cytoplasmic 1 | P60709 |  |  |  | 42052 | 5.29 | 203 | 5 (4) |
|  | Actin, cytoplasmic 2 | P63261 |  |  |  | 42108 | 5.31 | 170 | 5 (1) |
|  | Beta-actin-like protein 2 | Q562R1 |  |  |  | 42318 | 5.39 | 106 | 2 (0) |
|  | Keratin, type II cytoskeletal 1 | P04264 |  |  |  | 66149 | 8.16 | 59 | 1 |
|  | Creatine kinase B-type | P12277 |  |  |  | 42902 | 5.34 | 52 | 1 |
| 39 | Adenosylhomocysteinase | P23526 | U 1.79 | 45.8 | 6.09 | 48255 | 5.92 | 378 | 14 (13) |
|  | Keratin, type II cytoskeletal 1 | P04264 |  |  |  | 66149 | 8.16 | 51 | 1 |
|  | Retinal dehydrogenase 1 | P00352 |  |  |  | 55454 | 6.3 | 40 | 1 |
|  | Zinc finger MYM-type protein 1 | Q5SVZ6 |  |  |  | 131542 | 7.51 | 40 | 1 (0) |
|  | Retinal-specific ATP-binding cassette transporter | P78363 |  |  |  | 258232 | 5.89 | 40 | 1 (0) |
|  | Actin, cytoplasmic 2 | P63261 |  |  |  | 42108 | 5.31 | 160 | 4 |
|  | Beta-actin-like protein 2 | Q562R1 |  |  |  | 42318 | 5.39 | 94 | 2 (0) |
|  | Keratin, type II cytoskeletal 1 | P04264 |  |  |  | 66149 | 8.16 | 73 | 1 |
| 40 | Actin, cytoplasmic 1 | P60709 | U 2.16 | 45.7 | 4.46 | 42052 | 5.29 | 265 | 8 (7) |
|  | Beta-actin-like protein 2 | Q562R1 |  |  |  | 42318 | 5.39 | 97 | 2 (0) |
|  | Actin, cytoplasmic 2 | P63261 |  |  |  | 42108 | 5.31 | 87 | 2 (0) |
|  | Keratin, type II cytoskeletal 1 | P04264 |  |  |  | 66149 | 8.16 | 41 | 1 |
| 41 | Integrin-linked kinase-associated serine/threonine phosphatase 2C | Q9H0C8 | U 2.4 | 45.7 | 6.94 | 43450 | 6.68 | 163 | 5 |
|  | Multifunctional protein ADE2 | P22234 |  |  |  | 47790 | 6.94 | 115 | 2 |
|  | 26S protease regulatory subunit 8 | P62195 |  |  |  | 45768 | 7.11 | 246 | 10 (9) |
|  | Fumarate hydratase, mitochondrial | P07954 |  |  |  | 54773 | 8.85 | 40 | 1 |
|  | Keratin, type II cytoskeletal 1 | P04264 |  |  |  | 66149 | 8.16 | 60 | 1 |
|  | 6-phosphogluconate dehydrogenase, decarboxylating | P52209 |  |  |  | 53619 | 6.8 | 56 | 1 |
| 42 | Actin, cytoplasmic 1 | P60709 | D - 3 | 45.2 | 4.12 | 42052 | 5.29 | 327 | 13 (10) |
| 42 | Actin, cytoplasmic 2 | P63261 | D - 3 | 45.2 | 4.12 | 42108 | 5.31 | 288 | 13 (2) |
|  | Beta-actin-like protein 2 | Q562R1 |  |  |  | 42318 | 5.39 | 102 | 4 (0) |
|  | Creatine kinase B-type | P12277 |  |  |  | 42902 | 5.34 | 148 | 5 (4) |
|  | Keratin, type II cytoskeletal 1 | P04264 |  |  |  | 66149 | 8.16 | 68 | 1 |
| 43 | Leukocyte elastase inhibitor | P30740 | D* | 44.1 | 6.09 | 42829 | 5.9 | 146 | 4 |
|  | Elongation factor 1-gamma | P26641 |  |  |  | 50429 | 6.25 | 107 | 2 |
| 44 | Heterogeneous nuclear ribonucleoproteins C1/C2 | P07910 | D* | 43.9 | 5.01 | 33707 | 4.95 | 216 | 6 |
|  | 40S ribosomal protein SA | P08865 |  |  |  | 32947 | 4.79 | 114 | 3 |
|  | Heterogeneous nuclear ribonucleoprotein C-like 1 | O60812 |  |  |  | 32180 | 4.93 | 92 | 4 (0) |
|  | Keratin, type I cytoskeletal 18 | P05783 |  |  |  | 48029 | 5.34 | 69 | 2 |
|  | Keratin, type I cytoskeletal 19 | P08727 |  |  |  | 44065 | 5.05 | 58 | 2 (1) |
|  | Proteasomal ubiquitin receptor ADRM1 | Q16186 |  |  |  | 42412 | 4.96 | 51 | 2 (1) |
|  | Keratin, type II cytoskeletal 8 | P05787 |  |  |  | 53671 | 5.52 | 48 | 1 |
| 45 | Mannose-6-phosphate isomerase | P34949 | D* | 43.8 | 5.66 | 47196 | 5.62 | 43 | 1 |
| 46 | Septin-2 | Q15019 | U* | 43.9 | 6.52 | 41689 | 6.15 | 142 | 3 |
| 47 | 26S proteasome non-ATPase regulatory subunit 13 | Q9UNM6 | D* | 43.5 | 5.74 | 43176 | 5.53 | 136 | 3 |
|  | TAR DNA-binding protein 43 | Q13148 |  |  |  | 45053 | 5.85 | 78 | 3 |
|  | Actin, cytoplasmic 1 | P60709 |  |  |  | 42052 | 5.29 | 63 | 3 |
|  | Actin, cytoplasmic 2 | P63261 |  |  |  | 42108 | 5.31 | 63 | 3 (0) |
|  | Beta-actin-like protein 2 | Q562R1 |  |  |  | 42318 | 5.39 | 53 | 2 (0) |
| 48 | Poly(rC)-binding protein 1 | Q15365 | D* | 43.2 | 6.23 | 37987 | 6.66 | - | - |
|  | Poly(rC)-binding protein 2 | Q15366 |  |  |  | 38955 | 6.33 | 87 | 2 |
|  | Poly(rC)-binding protein 3 | P57721 |  |  |  | 36201 | 8.21 | 87 | 2 (0) |
| 49 | Macrophage-capping protein | P40121 | D* | 42.4 | 6 | 38779 | 5.88 | 117 | 3 |
| 50 | PDZ domain-containing protein GIPC1 | O14908 | U 1.80 | 41.5 | 6.3 | 36141 | 5.9 | 157 | 5 |
|  | Poly(rC)-binding protein 1 | Q15365 |  |  |  | 37987 | 6.66 | 43 | 1 |
|  | Poly(rC)-binding protein 2 | Q15366 |  |  |  | 38955 | 6.33 | 43 | 1 (0) |
|  | Poly(rC)-binding protein 3 | P57721 |  |  |  | 36201 | 8.21 | 43 | 1 (0) |
|  | Acetyl-CoA acetyltransferase, cytosolic | Q9BWD1 |  |  |  | 41838 | 6.47 | 81 | 3 |
|  | Keratin, type II cytoskeletal 1 | P04264 |  |  |  | 66149 | 8.16 | 79 | 1 |
|  | Eukaryotic translation initiation factor 3 subunit H | O15372 |  |  |  | 40076 | 6.09 | 75 | 2 |
| 51 | Phosphoserine aminotransferase | Q9Y617 | U 1.78 | 41.3 | 7 | 40796 | 7.56 | 178 | 4 |
|  | Aspartate aminotransferase, cytoplasmic | P17174 |  |  |  | 46447 | 6.53 | 144 | 4 (3) |
|  | Mitochondrial import receptor subunit TOM40 homolog | O96008 |  |  |  | 38211 | 6.79 | 117 | 5 (4) |
|  | Nuclear inhibitor of protein phosphatase 1 | Q12972 |  |  |  | 38626 | 6.87 | 103 | 5 (4) |
|  | Dynein heavy chain domain-containing protein 2 | Q6ZR08 |  |  |  | 126398 | 5.94 | 40 | 2 (1) |
| 52 | UF0160 protein MYG1 | Q9HB07 | D* | 41.1 | 5.83 | 42761 | 6.25 | 46 | 1 |
| 53 | Sialic acid synthase | Q9NR45 | U 1.9 | 40 | 6.72 | 40738 | 6.29 | 163 | 5 |
|  | Alcohol dehydrogenase [NADP+] | P14550 |  |  |  | 36892 | 6.32 | 117 | 3 |
|  | Poly(rC)-binding protein 1 | Q15365 |  |  |  | 37987 | 6.66 | 69 | 1 |
|  | Poly(rC)-binding protein 2 | Q15366 |  |  |  | 38955 | 6.33 | 69 | 1 (0) |
|  | Poly(rC)-binding protein 3 | P57721 |  |  |  | 36201 | 8.21 | 69 | 1 (0) |
| 54 | Serine-threonine kinase receptor-associated protein | Q9Y3F4 | U 3.63 | 39.7 | 5.08 | 38756 | 4.98 | 283 | 7 (6) |
|  | Heterogeneous nuclear ribonucleoproteins C1/C2 | P07910 |  |  |  | 33707 | 4.95 | 127 | 4 |
|  | Nucleophosmin | P06748 |  |  |  | 32726 | 4.64 | 135 | 5 (3) |
| 55 | Serine-threonine kinase receptor-associated protein | Q9Y3F4 | D* | 39.4 | 4.45 | 38756 | 4.98 | 248 | 6 |
|  | Heterogeneous nuclear ribonucleoproteins C1/C2 | P07910 |  |  |  | 33707 | 4.95 | 73 | 3 |
| 56 | Eukaryotic translation initiation factor 3 subunit I | Q13347 | U 1.97 | 39.1 | 5.42 | 36878 | 5.38 | 67 | 2 |
|  | Keratin, type II cytoskeletal 1 | P04264 |  |  |  | 66149 | 8.16 | 65 | 1 |
| 57 | Transaldolase | P37837 | U* | 39.4 | 6.3 | 37688 | 6.36 | 96 | 2 |
| 58 | Transaldolase | P37837 | D* | 38.1 | 6.01 | 37688 | 6.36 | 185 | 4 |
|  | Tubulin alpha-1A chain | Q71U36 |  |  |  | 50788 | 4.94 | 155 | 4 |
|  | Tubulin alpha-1B chain | P68363 |  |  |  | 50804 | 4.94 | 155 | 4 (0) |
|  | Tubulin alpha-1C chain | Q9BQE3 |  |  |  | 50548 | 4.96 | 155 | 4 (0) |
|  | Serine/threonine-protein phosphatase PP1-alpha catalytic subunit | P62136 |  |  |  | 38229 | 5.94 | 69 | 1 |
|  | Keratin, type II cytoskeletal 1 | P04264 |  |  |  | 66149 | 8.16 | 61 | 1 |
|  | 60S acidic ribosomal protein P0-like | Q8NHW5 |  |  |  | 34514 | 5.41 | 55 | 1 |
|  | 60S acidic ribosomal protein P0 | P05388 |  |  |  | 34423 | 5.72 | 55 | 1 (0) |
| 59 | Scavenger mRNA-decapping enzyme DcpS | Q96C86 | U 1.74 | 37.4 | 6.12 | 38756 | 5.93 | 280 | 10 (7) |
|  | Serine/threonine-protein phosphatase PP1-alpha catalytic subunit | P62136 |  |  |  | 38229 | 5.94 | 227 | 6 (5) |
|  | Serine/threonine-protein phosphatase PP1-gamma catalytic subunit | P36873 |  |  |  | 37701 | 6.12 | 177 | 4 (1) |
|  | Protein CDV3 homolog | Q9UKY7 |  |  |  | 27318 | 6.06 | 136 | 6 (5) |
|  | Keratin, type II cytoskeletal 5 | P13647 |  |  |  | 62568 | 7.58 | 68 | 1 |
|  | Keratin, type II cytoskeletal 6A | P02538 |  |  |  | 60293 | 8.09 | 68 | 1 (0) |
|  | Keratin, type II cytoskeletal 6B | P04259 |  |  |  | 60274 | 8.09 | 68 | 1 (0) |
|  | Keratin, type II cytoskeletal 6C | P48668 |  |  |  | 60273 | 8.09 | 68 | 1 (0) |
|  | Keratin, type II cytoskeletal 75 | O95678 |  |  |  | 59753 | 7.6 | 68 | 1 (0) |
|  | Keratin, type II cytoskeletal 79 | Q5XKE5 |  |  |  | 58059 | 6.75 | 68 | 1 (0) |
|  | Keratin, type II cytoskeletal 2 epidermal | P35908 |  |  |  | 66111 | 8.07 | 68 | 1 (0) |
|  | Keratin, type II cytoskeletal 1 | P04264 |  |  |  | 66149 | 8.16 | 60 | 1 |
|  | Glyceraldehyde-3-phosphate dehydrogenase | P04406 |  |  |  | 36201 | 8.57 | 42 | 2 |
|  | Mucin and cadherin-like protein | Q9HBB8 |  |  |  | 88444 | 4.78 | 41 | 1 (0) |
|  | Abnormal spindle-like microcephaly-associated protein | Q8IZT6 |  |  |  | 413189 | 10.45 | 33 | 1 (0) |
| 60 | 60S acidic ribosomal protein P0 | P05388 | D* | 37.3 | 6.05 | 34423 | 5.72 | 253 | 9 (8) |
|  | Macrophage-capping protein | P40121 |  |  |  | 38779 | 5.88 | 92 | 4 |
|  | Keratin, type II cytoskeletal 5 | P13647 |  |  |  | 62568 | 7.58 | 47 | 1 |
|  | Keratin, type II cytoskeletal 6A | P02538 |  |  |  | 60293 | 8.09 | 47 | 1 (0) |
|  | Keratin, type II cytoskeletal 6B | P04259 |  |  |  | 60274 | 8.09 | 47 | 1 (0) |
|  | Keratin, type II cytoskeletal 6C | P48668 |  |  |  | 60273 | 8.09 | 47 | 1 (0) |
|  | Keratin, type II cytoskeletal 75 | O95678 |  |  |  | 59753 | 7.6 | 47 | 1 (0) |
|  | Keratin, type II cytoskeletal 79 | Q5XKE5 |  |  |  | 58059 | 6.75 | 47 | 1 (0) |
|  | Keratin, type II cytoskeletal 2 epidermal | P35908 |  |  |  | 66111 | 8.07 | 46 | 1 (0) |
| 61 | 60S acidic ribosomal protein P0 | P05388 | D* | 37.3 | 5.61 | 34423 | 5.72 | 661 | 27 (15) |
|  | UF0553 protein C9orf64 | Q5T6V5 |  |  |  | 39460 | 5.61 | 379 | 12 (10) |
| 62 | UF0553 protein C9orf64 | Q5T6V5 | D* | 36.7 | 5.61 | 39460 | 5.61 | 88 | 3 |
|  | F-actin-capping protein subunit alpha-1 | P52907 |  |  |  | 33073 | 5.45 | 83 | 4 (2) |
|  | Keratin, type II cytoskeletal 1 | P04264 |  |  |  | 66149 | 8.16 | 61 | 1 |
|  | Keratin, type II cytoskeletal 5 | P13647 |  |  |  | 62568 | 7.58 | 35 | 1 |
|  | Keratin, type II cytoskeletal 6A | P02538 |  |  |  | 60293 | 8.09 | 35 | 1 (0) |
|  | Keratin, type II cytoskeletal 6B | P04259 |  |  |  | 60274 | 8.09 | 35 | 1 (0) |
|  | Keratin, type II cytoskeletal 6C | P48668 |  |  |  | 60273 | 8.09 | 35 | 1 (0) |
|  | Keratin, type II cytoskeletal 75 | O95678 |  |  |  | 59753 | 7.6 | 35 | 1 (0) |
|  | Keratin, type II cytoskeletal 79 | Q5XKE5 |  |  |  | 58059 | 6.75 | 35 | 1 (0) |
| 63 | Nicotinate-nucleotide pyrophosphorylase [carboxylating] | Q15274 | D* | 36.5 | 5.96 | 31138 | 5.81 | 53 | 1 |
|  | Keratin, type II cytoskeletal 5 | P13647 |  |  |  | 62568 | 7.58 | 46 | 1 |
|  | Keratin, type II cytoskeletal 6A | P02538 |  |  |  | 60293 | 8.09 | 46 | 1 (0) |
|  | Keratin, type II cytoskeletal 6B | P04259 |  |  |  | 60274 | 8.09 | 46 | 1 (0) |
|  | Keratin, type II cytoskeletal 6C | P48668 |  |  |  | 60273 | 8.09 | 46 | 1 (0) |
|  | Keratin, type II cytoskeletal 75 | O95678 |  |  |  | 59753 | 7.6 | 46 | 1 (0) |
|  | Keratin, type II cytoskeletal 79 | Q5XKE5 |  |  |  | 58059 | 6.75 | 46 | 1 (0) |
|  | Keratin, type II cytoskeletal 2 epidermal | P35908 |  |  |  | 66111 | 8.07 | 44 | 1 (0) |
| 64 | Tubulin beta chain | P07437 | D -2.2 | 35.7 | 5.74 | 50095 | 4.78 | 233 | 7 |
|  | Phosphoglycolate phosphatase | A6NDG6 |  |  |  | 34441 | 5.85 | 224 | 5 (4) |
|  | Tubulin beta-2C chain | P68371 |  |  |  | 50255 | 4.79 | 194 | 7 (2) |
|  | Tubulin beta-3 chain | Q13509 |  |  |  | 50856 | 4.83 | 157 | 6 (0) |
|  | Tubulin beta-2A chain | Q13885 |  |  |  | 50274 | 4.78 | 156 | 5 (0) |
|  | Tubulin beta-2B chain | Q9BVA1 |  |  |  | 50377 | 4.78 | 156 | 5 (0) |
|  | Tubulin beta-6 chain | Q9BUF5 |  |  |  | 50281 | 4.77 | 132 | 3 (0) |
|  | Keratin, type II cytoskeletal 5 | P13647 |  |  |  | 62568 | 7.58 | 50 | 1 |
|  | Keratin, type II cytoskeletal 6A | P02538 |  |  |  | 60293 | 8.09 | 50 | 1 (0) |
|  | Keratin, type II cytoskeletal 6B | P04259 |  |  |  | 60274 | 8.09 | 50 | 1 (0) |
|  | Keratin, type II cytoskeletal 6C | P48668 |  |  |  | 60273 | 8.09 | 50 | 1 (0) |
|  | Keratin, type II cytoskeletal 75 | O95678 |  |  |  | 59753 | 7.6 | 50 | 1 (0) |
|  | Keratin, type II cytoskeletal 79 | Q5XKE5 |  |  |  | 58059 | 6.75 | 50 | 1 (0) |
|  | Keratin, type II cytoskeletal 2 epidermal | P35908 |  |  |  | 66111 | 8.07 | 48 | 1 (0) |
|  | Annexin A4 | P09525 |  |  |  | 36088 | 5.84 | 35 | 1 |
| 65 | Annexin A1 | P04083 | U 5.06 | 35.8 | 6.39 | 38918 | 6.57 | 176 | 3 |
|  | UDP-glucose 4-epimerase | Q14376 |  |  |  | 38656 | 6.26 | 73 | 2 |
|  | Keratin, type II cytoskeletal 1 | P04264 |  |  |  | 66149 | 8.16 | 71 | 1 |
|  | Serine/threonine-protein phosphatase PP1-gamma catalytic subunit | P36873 |  |  |  | 37701 | 6.12 | 60 | 1 |
|  | Annexin A2 | P07355 |  |  |  | 38808 | 7.57 | 56 | 1 |
|  | AH receptor-interacting protein | O00170 |  |  |  | 38096 | 6.09 | 46 | 2 |
|  | Keratin, type II cytoskeletal 5 | P13647 |  |  |  | 62568 | 7.58 | 40 | 1 |
|  | Keratin, type II cytoskeletal 6A | P02538 |  |  |  | 60293 | 8.09 | 40 | 1 (0) |
|  | Keratin, type II cytoskeletal 6B | P04259 |  |  |  | 60274 | 8.09 | 40 | 1 (0) |
| 65 | Keratin, type II cytoskeletal 6C | P48668 | U 5.06 | 35.8 | 6.39 | 60273 | 8.09 | 40 | 1 (0) |
|  | Keratin, type II cytoskeletal 75 | O95678 |  |  |  | 59753 | 7.6 | 40 | 1 (0) |
|  | Keratin, type II cytoskeletal 79 | Q5XKE5 |  |  |  | 58059 | 6.75 | 40 | 1 (0) |
|  | Keratin, type II cytoskeletal 2 epidermal | P35908 |  |  |  | 66111 | 8.07 | 38 | 1 (0) |
|  | Glyceraldehyde-3-phosphate dehydrogenase | P04406 |  |  |  | 36201 | 8.57 | 55 | 2 |
| 66 | Malate dehydrogenase, cytoplasmic | P40925 | U 3.42 | 35.8 | 6.59 | 36631 | 6.91 | 134 | 4 |
|  | LIM and SH3 domain protein 1 | Q14847 |  |  |  | 30097 | 6.61 | 111 | 3 |
|  | UDP-glucose 4-epimerase | Q14376 |  |  |  | 38656 | 6.26 | 77 | 3 |
|  | Keratin, type II cytoskeletal 1 | P04264 |  |  |  | 66149 | 8.16 | 77 | 1 |
|  | Annexin A2 | P07355 |  |  |  | 38808 | 7.57 | 58 | 1 |
|  | Keratin, type I cytoskeletal 9 | P35527 |  |  |  | 62320 | 5.19 | 39 | 1 |
| 67 | PDZ and LIM domain protein 1 | O00151 | U* | 35.9 | 6.7 | 36505 | 6.56 | 121 | 5 (4) |
| 68 | Annexin A1 | P04083 | U 2.66 | 35.6 | 6.7 | 38918 | 6.57 | 614 | 14 (13) |
|  | PDZ and LIM domain protein 1 | O00151 |  |  |  | 36505 | 6.56 | 159 | 6 (5) |
|  | Annexin A2 | P07355 |  |  |  | 38808 | 7.57 | 74 | 3 |
|  | Calponin-2 | Q99439 |  |  |  | 34074 | 6.94 | 59 | 1 |
|  | Glyceraldehyde-3-phosphate dehydrogenase | P04406 |  |  |  | 36201 | 8.57 | 40 | 2 |
| 69 | Keratin, type II cytoskeletal 1 | P04264 | D* | 35 | 5.67 | 66149 | 8.16 | 58 | 1 |
|  | BRCA1/BRCA2-containing complex subunit 3 | P46736 |  |  |  | 36448 | 5.59 | 35 | 2 |
| 70 | DnaJ homolog subfamily C member 8 | O75937 | D* | 33.9 | 4.17 | 29823 | 9.04 | 71 | 3 |
|  | Annexin A5 | P08758 |  |  |  | 35971 | 4.94 | 57 | 1 |
|  | Keratin, type II cytoskeletal 1 | P04264 |  |  |  | 66149 | 8.16 | 46 | 1 |
| 71 | Glyceraldehyde-3-phosphate dehydrogenase | P04406 | D* | 33.1 | 7.02 | 36201 | 8.57 | 221 | 8 (7) |
|  | Thiosulfate sulfurtransferase | Q16762 |  |  |  | 33636 | 6.77 | 64 | 2 |
|  | Pyrroline-5-carboxylate reductase 1, mitochondrial | P32322 |  |  |  | 33568 | 7.18 | 37 | 1 |
|  | Pyrroline-5-carboxylate reductase 2 | Q96C36 |  |  |  | 33958 | 7.66 | 37 | 1 (0) |
| 72 | Annexin A4 | P09525 | D* | 33 | 5.55 | 36088 | 5.84 | 341 | 6 |
|  | Proteasome activator complex subunit 3 | P61289 |  |  |  | 29602 | 5.69 | 154 | 3 |
|  | Keratin, type II cytoskeletal 1 | P04264 |  |  |  | 66149 | 8.16 | 66 | 1 |
|  | Serine/threonine-protein phosphatase 6 catalytic subunit | O00743 |  |  |  | 35806 | 5.43 | 56 | 2 |
| 73 | Glyceraldehyde-3-phosphate dehydrogenase | P04406 | D* | 32.7 | 6.98 | 36201 | 8.57 | 188 | 9 (6) |
|  | Thiosulfate sulfurtransferase | Q16762 |  |  |  | 33636 | 6.77 | 52 | 2 |
| 74 | Tropomyosin alpha-4 chain | P67936 | U* | 33.1 | 4.8 | 28619 | 4.67 | 38 | 1 |
| 75 | Coatomer subunit epsilon | O14579 | U 3.48 | 32.7 | 5.01 | 34688 | 4.98 | 212 | 7 |
|  | 40S ribosomal protein S3 | P23396 |  |  |  | 26842 | 9.68 | 147 | 6 |
|  | Annexin A5 | P08758 |  |  |  | 35971 | 4.94 | 126 | 2 |
|  | Actin, cytoplasmic 1 | P60709 |  |  |  | 42052 | 5.29 | 112 | 3 |
|  | Actin, cytoplasmic 2 | P63261 |  |  |  | 42108 | 5.31 | 112 | 3 (0) |
|  | Chloride intracellular channel protein 1 | O00299 |  |  |  | 27248 | 5.09 | 92 | 3 |
|  | Beta-actin-like protein 2 | Q562R1 |  |  |  | 42318 | 5.39 | 91 | 2 (0) |
|  | EF-hand domain-containing protein D2 | Q96C19 |  |  |  | 26795 | 5.15 | 72 | 1 |
|  | Keratin, type II cytoskeletal 1 | P04264 |  |  |  | 66149 | 8.16 | 53 | 1 |
|  | U1 small nuclear ribonucleoprotein A | P09012 |  |  |  | 31259 | 9.83 | 49 | 3 |
| 75 | Keratin, type II cytoskeletal 5 | P13647 | U 3.48 | 32.7 | 5.01 | 62568 | 7.58 | 37 | 1 |
|  | Keratin, type II cytoskeletal 6A | P02538 |  |  |  | 60293 | 8.09 | 37 | 1 (0) |
|  | Keratin, type II cytoskeletal 6B | P04259 |  |  |  | 60274 | 8.09 | 37 | 1 (0) |
|  | Keratin, type II cytoskeletal 6C | P48668 |  |  |  | 60273 | 8.09 | 37 | 1 (0) |
|  | Keratin, type II cytoskeletal 75 | O95678 |  |  |  | 59753 | 7.6 | 37 | 1 (0) |
|  | Keratin, type II cytoskeletal 79 | Q5XKE5 |  |  |  | 58059 | 6.75 | 37 | 1 (0) |
| 76 | Proteasome activator complex subunit 3 | P61289 | D* | 32.6 | 5.57 | 29602 | 5.69 | 53 | 1 |
|  | Toll-interacting protein | Q9H0E2 |  |  |  | 30490 | 5.68 | 41 | 2 |
| 77 | 14-3-3 protein epsilon | P62258 | D -3 | 31.7 | 4.75 | 29326 | 4.63 | 282 | 8 |
|  | 14-3-3 protein theta | P27348 |  |  |  | 28032 | 4.68 | 53 | 2 (0) |
| 78 | Electron transfer flavoprotein subunit alpha, mitochondrial | P13804 | U 2.37 | 31.3 | 6.93 | 35400 | 8.62 | 205 | 8 |
|  | Voltage-dependent anion-selective channel protein 2 | P45880 |  |  |  | 32060 | 7.5 | 174 | 5 |
|  | V-type proton ATPase subunit E 1 | P36543 |  |  |  | 26186 | 7.7 | 95 | 4 (2) |
|  | Keratin, type II cytoskeletal 1 | P04264 |  |  |  | 66149 | 8.16 | 77 | 1 |
|  | Keratin, type II cytoskeletal 5 | P13647 |  |  |  | 62568 | 7.58 | 53 | 1 |
|  | Keratin, type II cytoskeletal 6A | P02538 |  |  |  | 60293 | 8.09 | 53 | 1 (0) |
|  | Keratin, type II cytoskeletal 6B | P04259 |  |  |  | 60274 | 8.09 | 53 | 1 (0) |
|  | Keratin, type II cytoskeletal 6C | P48668 |  |  |  | 60273 | 8.09 | 53 | 1 (0) |
|  | Keratin, type II cytoskeletal 75 | O95678 |  |  |  | 59753 | 7.6 | 53 | 1 (0) |
|  | Keratin, type II cytoskeletal 79 | Q5XKE5 |  |  |  | 58059 | 6.75 | 53 | 1 (0) |
|  | Keratin, type II cytoskeletal 2 epidermal | P35908 |  |  |  | 66111 | 8.07 | 51 | 1 (0) |
|  | Proteasome assembly chaperone 1 | O95456 |  |  |  | 33631 | 6.88 | 52 | 2 |
|  | Glucosamine-6-phosphate isomerase 1 | P46926 |  |  |  | 32819 | 6.42 | 36 | 1 |
| 79 | Coiled-coil domain-containing protein 5 | Q96CS2 | D -2.43 | 32.6 | 5.35 | 31901 | 5.41 | 56 | 2 (1) |
|  | Keratin, type II cytoskeletal 1 | P04264 |  |  |  | 66149 | 8.16 | 54 | 1 |
| 80 | Isochorismatase domain-containing protein 1 | Q96CN7 | U 2.18 | 31.3 | 6.99 | 32501 | 6.96 | 188 | 6 |
|  | V-type proton ATPase subunit E 1 | P36543 |  |  |  | 26186 | 7.7 | 124 | 3 (2) |
|  | Syntenin-1 | O00560 |  |  |  | 32595 | 7.06 | 103 | 4 (3) |
|  | Keratin, type II cytoskeletal 1 | P04264 |  |  |  | 66149 | 8.16 | 74 | 1 |
|  | Electron transfer flavoprotein subunit alpha, mitochondrial | P13804 |  |  |  | 35400 | 8.62 | 61 | 2 |
|  | Protein angel homolog 1 | Q9UNK9 |  |  |  | 76595 | 4.72 | 37 | 1 (0) |
| 81 | Histamine N-methyltransferase | P50135 | D* | 31.1 | 5.23 | 33616 | 5.18 | 274 | 10 (6) |
|  | Tumor protein D54 | O43399 |  |  |  | 22281 | 5.26 | 82 | 2 |
| 82 | Keratin, type II cytoskeletal 1 | P04264 | D* | 30.9 | 4.43 | 66149 | 8.16 | 65 | 1 |
| 83 | Proteasome subunit alpha type-3 | P25788 | D* | 30.1 | 5.22 | 28643 | 5.19 | 310 | 9 (7) |
| 84 | Peroxiredoxin-6 | P30041 | U 2.79 | 25.8 | 6.03 | 25133 | 6 | 236 | 6 (5) |
|  | Fumarylacetoacetate hydrolase domain-containing protein 1 | Q6P587 |  |  |  | 25112 | 6.96 | 113 | 3 |
|  | Keratin, type II cytoskeletal 1 | P04264 |  |  |  | 66149 | 8.16 | 70 | 1 |
| 85 | Proteasome subunit alpha type-3 | P25788 | U 2.07 | 28.5 | 6.97 | 28643 | 5.19 | 262 | 7 (5) |
|  | Keratin, type I cytoskeletal 18 | P05783 |  |  |  | 48029 | 5.34 | 71 | 2 |
|  | Keratin, type II cytoskeletal 1 | P04264 |  |  |  | 66149 | 8.16 | 70 | 1 |
|  | Fumarylacetoacetate hydrolase domain-containing protein 1 | Q6P587 |  |  |  | 25112 | 6.96 | 54 | 2 |
| 85 | Carbonic anhydrase 2 | P00918 | U 2.07 | 28.5 | 6.97 | 29285 | 6.87 | 40 | 1 |
| 86 | Triosephosphate isomerase | P60174 | U* | 27.7 | 6.78 | 26938 | 6.45 | 130 | 3 |
|  | Keratin, type I cytoskeletal 18 | P05783 |  |  |  | 48029 | 5.34 | 52 | 1 |
|  | Keratin, type I cytoskeletal 19 | P08727 |  |  |  | 44065 | 5.05 | 52 | 1 (0) |
| 87 | Glutathione S-transferase P | P09211 | D * | 27.7 | 5.6 | 23569 | 5.43 | 155 | 5 |
| 88 | Glutathione S-transferase P | P09211 | D* | 26.8 | 6.23 | 23569 | 5.43 | 266 | 7 |
|  | Proteasome subunit beta type-3 | P49720 |  |  |  | 23219 | 6.14 | 189 | 4 (3) |
|  | High mobility group protein B1 | P09429 |  |  |  | 25049 | 5.62 | 47 | 2 (1) |
|  | Putative high mobility group protein 1-like 1 | B2RPK0 |  |  |  | 24394 | 5.92 | 47 | 2 (0) |
|  | High mobility group protein 1-like 10 | Q9UGV6 |  |  |  | 24374 | 6.99 | 47 | 2 (0) |
| 89 | Glutathione S-transferase P | P09211 | U 1.61 | 26.6 | 5.94 | 23569 | 5.43 | 507 | 16 (14) |
|  | Oligoribonuclease, mitochondrial | Q9Y3B8 |  |  |  | 27044 | 6.4 | 46 | 2 |
|  | Proteasome subunit beta type-4 | P28070 |  |  |  | 29243 | 5.72 | 35 | 1 |
| 90 | Uncharacterized protein C9orf142 | Q9BUH6 | D -2.57 | 26.1 | 5.23 | 21968 | 5.39 | 50 | 1 |
|  | Gamma-glutamyltranspeptidase 1 | P19440 |  |  |  | 61714 | 6.65 | 49 | 1 |
|  | Gamma-glutamyltranspeptidase 2 | P36268 |  |  |  | 62074 | 7.22 | 49 | 1 (0) |
|  | Putative gamma-glutamyltranspeptidase 3 | A6NGU5 |  |  |  | 61919 | 6.68 | 49 | 1 (0) |
|  | Gamma-glutamyltransferase light chain 2 | Q14390 |  |  |  | 24206 | 5.13 | 49 | 1 (0) |
|  | Putative gamma-glutamyltransferase light chain 3 | B5MD39 |  |  |  | 24201 | 5.75 | 49 | 1 (0) |
| 91 | Proteasome subunit beta type-3 | P49720 | D* | 25.9 | 5.09 | 23219 | 6.14 | 62 | 2 |
|  | Uncharacterized protein C9orf142 | Q9BUH6 |  |  |  | 21968 | 5.39 | 57 | 2 |
| 92 | Glutathione S-transferase P | P09211 | D* | 25.3 | 5.13 | 23569 | 5.43 | 139 | 5 |
|  | Cathepsin B | P07858 |  |  |  | 38766 | 5.88 | 44 | 3 (1) |
| 93 | Glutathione S-transferase P | P09211 | D* | 25.4 | 5.55 | 23569 | 5.43 | 287 | 8 |
|  | COMM domain-containing protein 3 | Q9UBI1 |  |  |  | 22365 | 5.62 | 76 | 1 |
| 94 | Superoxide dismutase [Mn], mitochondrial | P04179 | U 1.87 | 24.3 | 6.92 | 24878 | 8.35 | 192 | 5 |
|  | Glutathione S-transferase P | P09211 |  |  |  | 23569 | 5.43 | 100 | 5 |
| 95 | Superoxide dismutase [Mn], mitochondrial | P04179 | D* | 23.8 | 5.22 | 24878 | 8.35 | 55 | 1 |
|  | RNA-binding protein 8A | Q9Y5S9 |  |  |  | 19934 | 5.5 | 50 | 2 |
| 96 | Phosphatidylethanolamine-binding protein 1 | P30086 | U 1.98 | 21 | 6.98 | 21158 | 7.01 | 81 | 3 |
| 97 | Nucleoside diphosphate kinase A | P15531 | D* | 19.8 | 6.38 | 17309 | 5.83 | 52 | 2 |
| 98 | 60S ribosomal protein L11 | P62913 | D* | 19.5 | 4.5 | 20468 | 9.64 | 239 | 5 (4) |
|  | 60S ribosomal protein L12 | P30050 |  |  |  | 17979 | 9.48 | 48 | 2 |
| 99 | Uncharacterized protein C6orf125 | Q9BRT2 | U 1.77 | 15.7 | 6.89 | 14979 | 6.84 | 35 | 2 |
| 100 | Keratin, type I cytoskeletal 10 | P13645 | U 2 | 15.2 | 6.53 | 59703 | 5.13 | 197 | 5 |
|  | Keratin, type II cytoskeletal 6A | P02538 |  |  |  | 60293 | 8.09 | 125 | 2 |
|  | Keratin, type II cytoskeletal 6B | P04259 |  |  |  | 60274 | 8.09 | 125 | 2 (0) |
|  | Keratin, type II cytoskeletal 6C | P48668 |  |  |  | 60273 | 8.09 | 125 | 2 (0) |
|  | Profilin-1 | P07737 |  |  |  | 15216 | 8.44 | 81 | 2 |
|  | Keratin, type II cytoskeletal 1 | P04264 |  |  |  | 66149 | 8.16 | 60 | 1 |
|  | Histidine triad nucleotide-binding protein 1 | P49773 |  |  |  | 13907 | 6.43 | 43 | 3 |

Fold Change are given: U = up regulated in HCV infected cells, U*=only detected in HCV infected cells

D = down regulated in HCV infected cells, D*=only detected in non-infected cells

Protein symbols

| **Protein Name** | **Database Entry** |
| --- | --- |
|
| Lamin-B2 | LMNB2 |
| Ras GTPase-activating protein-binding protein 1 | G3BP1 |
| Anaphase-promoting complex subunit 7 | APC7 |
| Heterogeneous nuclear ribonucleoprotein K | HNRPK |
| Insulin-like growth factor 2 mRNA-binding protein 1 | IF2B1 |
| Regulator of chromosome condensation | RCC1 |
| COBW domain-containing protein 1 | CBWD1 |
| COBW domain-containing protein 2 | CBWD2 |
| Elongation factor 1-gamma | EF1G |
| Heat shock protein HSP 90-alpha | HS90A |
| Heat shock protein HSP 90-beta | HS90B |
| 60S acidic ribosomal protein P0 | RLA0 |
| UPF0553 protein C9orf64 | CI064 |
| Histamine N-methyltransferase | HNMT |
| Tumor protein D54 | TPD54 |
| Proteasome subunit alpha type-3 | PSA3 |
| Lamin-B1 | LMNB1 |
| Insulin-like growth factor 2 mRNA-binding protein 1 | IF2B1 |
| Insulin-like growth factor 2 mRNA-binding protein 2 | IF2B2 |
| Insulin-like growth factor 2 mRNA-binding protein 3 | IF2B3 |
| Leucine-rich repeat-containing protein 47 | LRC47 |
| Vinculin | VINC |
| Matrin-3 | MATR3 |
| ATP-citrate synthase | ACLY |
| Matrin-3 | MATR3 |
| Splicing factor 3 subunit 1 | SF3A1 |
| Matrin-3 | MATR3 |
| Caprin-1 | CAPR1 |
| Alpha-actinin-4 | ACTN4 |
| Ubiquitin carboxyl-terminal hydrolase 5 | UBP5 |
| Nucleolin | NUCL |
| Heat shock protein HSP 90-alpha | HS90A |
| Heat shock protein HSP 90-beta | HS90B |
| Procollagen-lysine,2-oxoglutarate 5-dioxygenase 3 | PLOD3 |
| 78 kDa glucose-regulated protein | GRP78 |
| Ezrin | EZRI |
| Rootletin | CROCC |
| Early endosome antigen 1 | EEA1 |
| Protein phosphatase 1G | PPM1G |
| Heat shock cognate 71 kDa protein | HSP7C |
| Heat shock-related 70 kDa protein 2 | HSP72 |
| ATP-dependent DNA helicase 2 subunit | KU70 |
| Sorting nexin-2 | SNX2 |
| Heat shock cognate 71 kDa protein | HSP7C |
| Heat shock-related 70 kDa protein 2 | HSP72 |
| Sorting nexin-2 | SNX2 |
| ATP-dependent DNA helicase 2 subunit 1 | KU70 |
| Alpha-fetoprotein | FETA |
| Coronin-1B | COR1B |
| Stress-70 protein, mitochondrial | GRP75 |
| Annexin A6 | ANXA6 |
| Heat shock 70 kDa protein 1 | HSP71 |
| Insulin-like growth factor 2 mRNA-binding protein 1 | IF2B1 |
| Plastin-3 | PLST |
| Heterogeneous nuclear ribonucleoprotein K | HNRPK |
| Poly(U)-binding-splicing factor PUF60 | PUF60 |
| 60 kDa heat shock protein, mitochondrial | CH60 |
| Keratin, type II cytoskeletal 1 | K2C1 |
| Stress-70 protein, mitochondrial | GRP75 |
| Beta-galactosidase | BGAL |
| Pyruvate kinase isozymes M1/M2 | KPYM |
| UDP-N-acetylhexosamine pyrophosphorylase | UAP1 |
| T-complex protein 1 subunit theta | TCPQ |
| 60 kDa heat shock protein, mitochondrial | CH60 |
| T-complex protein 1 subunit epsilon | TCPE |
| Splicing factor 3A subunit 3 | SF3A3 |
| Fatty acid synthase | FAS |
| Heterogeneous nuclear ribonucleoprotein K | HNRPK |
| T-complex protein 1 subunit theta | TCPQ |
| Zinc finger CCCH domain-containing protein 15 | ZC3HF |
| 60 kDa heat shock protein, mitochondrial | CH60 |
| Nucleosome assembly protein 1-like 1 | NP1L1 |
| V-type proton ATPase subunit B, brain isoform | VATB2 |
| Protein disulfide-isomerase A3 | PDIA3 |
| Adenylyl cyclase-associated protein 1 | CAP1 |
| T-complex protein 1 subunit delta | TCPD |
| T-complex protein 1 subunit eta | TCPH |
| Protein disulfide-isomerase A3 | PDIA3 |
| Probable Xaa-Pro aminopeptidase 3 | XPP3 |
| Retinal dehydrogenase 1 | AL1A1 |
| Mitochondrial-processing peptidase subunit alpha | MPPA |
| Polyadenylate-binding protein 2 | PABP2 |
| Uncharacterized protein C6orf174 | CF174 |
| Tetratricopeptide repeat protein 38 | TTC38 |
| Elongation factor 1-alpha 1 | EF1A1 |
| Putative elongation factor 1-alpha-like 3 | EF1A3 |
| Seryl-tRNA synthetase, mitochondrial | SYSM |
| Elongation factor 1-alpha 1 | EF1A1 |
| Putative elongation factor 1-alpha-like 3 | EF1A3 |
| Septin-10 | FLJ11619 |
| Glutamate dehydrogenase 1, mitochondrial | DHE3 |
| Fascin | FSCN1 |
| ATP synthase subunit alpha, mitochondrial | ATPA |
| Keratin, type II cytoskeletal 8 | K2C8 |
| Coiled-coil domain-containing protein 110 | CC110 |
| Elongation factor 1-alpha 1 | EF1A1 |
| Elongation factor 1-alpha 2 | EF1A2 |
| Putative elongation factor 1-alpha-like 3 | EF1A3 |
| RuvB-like 2 | RUVB2 |
| Lupus La protein | LA |
| Elongation factor 1-alpha 1 | EF1A1 |
| Putative elongation factor 1-alpha-like 3 | EF1A3 |
| Alpha-enolase | ENOA |
| Phenylalanine-4-hydroxylase | PH4H |
| Keratin, type II cytoskeletal 1 | K2C1 |
| Elongation factor 1-alpha 1 | EF1A1 |
| Putative elongation factor 1-alpha-like 3 | EF1A3 |
| 60 kDa heat shock protein, mitochondrial | CH60 |
| 26S protease regulatory subunit 7 | PRS7 |
| Annexin A7 | ANXA7 |
| Rab GDP dissociation inhibitor beta | GDIB |
| Adenylosuccinate synthetase isozyme 2 | PURA2 |
| Tubulin alpha-1A chain | TBA1A |
| Tubulin alpha-1B chain | TBA1B |
| Actin, cytoplasmic 1 | ACTB |
| Actin, cytoplasmic 2 | ACTG |
| Beta-actin-like protein 2 | ACTBL |
| Adenosylhomocysteinase | SAHH |
| Keratin, type II cytoskeletal 1 | K2C1 |
| Retinal dehydrogenase 1 | AL1A1 |
| Zinc finger MYM-type protein 1 | ZMYM1 |
| Retinal-specific ATP-binding cassette transporter | ABCA4 |
| Actin, cytoplasmic 1 | ACTB |
| Beta-actin-like protein 2 | ACTBL |
| Integrin-linked kinase-associated serine/threonine phosphatase 2C | ILKAP |
| Multifunctional protein ADE2 | PUR6 |
| 26S protease regulatory subunit 8 | PRS8 |
| Fumarate hydratase, mitochondrial | FUMH |
| Actin, cytoplasmic 1 | ACTB |
| Actin, cytoplasmic 2 | ACTG |
| Beta-actin-like protein 2 | ACTBL |
| Leukocyte elastase inhibitor | ILEU |
| Elongation factor 1-gamma | EF1G |
| Heterogeneous nuclear ribonucleoproteins C1/C2 | HNRPC |
| 40S ribosomal protein SA | RSSA |
| Heterogeneous nuclear ribonucleoprotein C-like 1 | HNRCL |
| Keratin, type I cytoskeletal 18 | K1C18 |
| Keratin, type I cytoskeletal 19 | K1C19 |
| Proteasomal ubiquitin receptor ADRM1 | ADRM1 |
| Keratin, type II cytoskeletal 8 | K2C8 |
| Mannose-6-phosphate isomerase | MPI |
| 26S proteasome non-ATPase regulatory subunit 13 | PSD13 |
| TAR DNA-binding protein 43 | TADBP |
| Actin, cytoplasmic 1 | ACTB |
| Actin, cytoplasmic 2 | ACTG |
| Beta-actin-like protein 2 | ACTBL |
| Poly(rC)-binding protein 1 | PCBP1 |
| Poly(rC)-binding protein 2 | PCBP2 |
| Poly(rC)-binding protein 3 | PCBP3 |
| Macrophage-capping protein | CAPG |
| PDZ domain-containing protein GIPC1 | GIPC1 |
| Poly(rC)-binding protein 1 | PCBP1 |
| Poly(rC)-binding protein 2 | PCBP2 |
| Poly(rC)-binding protein 3 | PCBP3 |
| Phosphoserine aminotransferase | SERC |
| Aspartate aminotransferase, cytoplasmic | AATC |
| Mitochondrial import receptor subunit TOM40 homolog | TOM40 |
| Nuclear inhibitor of protein phosphatase 1 | PP1R8 |
| UPF0160 protein MYG1 | MYG1 |
| Sialic acid synthase | SIAS |
| Alcohol dehydrogenase [NADP+] | AK1A1 |
| Poly(rC)-binding protein 1 | PCBP1 |
| Poly(rC)-binding protein 2 | PCBP2 |
| Poly(rC)-binding protein 3 | PCBP3 |
| Serine-threonine kinase receptor-associated protein | STRAP |
| Heterogeneous nuclear ribonucleoproteins C1/C2 | HNRPC |
| Nucleophosmin | NPM |
| Serine-threonine kinase receptor-associated protein | STRAP |
| Heterogeneous nuclear ribonucleoproteins C1/C2 | HNRPC |
| Eukaryotic translation initiation factor 3 subunit I | EIF3I |
| Keratin, type II cytoskeletal 1 | K2C1 |
| Transaldolase | TALDO |
| Tubulin alpha-1A chain | TBA1A |
| Tubulin alpha-1B chain | TBA1B |
| Tubulin alpha-1C chain | TBA1C |
| Serine/threonine-protein phosphatase PP1-alpha catalytic subunit | PP1A |
| Keratin, type II cytoskeletal 1 | K2C1 |
| 60S acidic ribosomal protein P0-like | RLA0L |
| 60S acidic ribosomal protein P0 | RLA0 |
| Scavenger mRNA-decapping enzyme DcpS | DCPS |
| Serine/threonine-protein phosphatase PP1-alpha catalytic subunit | PP1A |
| Serine/threonine-protein phosphatase PP1-gamma catalytic subunit | PP1G |
| Protein CDV3 homolog | CDV3 |
| Keratin, type II cytoskeletal 5 | K2C5 |
| Keratin, type II cytoskeletal 6A | K2C6A |
| Keratin, type II cytoskeletal 6B | K2C6B |
| Keratin, type II cytoskeletal 6C | K2C6C |
| Keratin, type II cytoskeletal 75 | K2C75 |
| Keratin, type II cytoskeletal 79 | K2C79 |
| Keratin, type II cytoskeletal 2 epidermal | K22E |
| Keratin, type II cytoskeletal 1 | K2C1 |
| Glyceraldehyde-3-phosphate dehydrogenase | G3P |
| Mucin and cadherin-like protein | MUCDL |
| Abnormal spindle-like microcephaly-associated protein | ASPM |
| 60S acidic ribosomal protein P0 | RLA0 |
| Macrophage-capping protein | CAPG |
| Keratin, type II cytoskeletal 5 | K2C5 |
| Keratin, type II cytoskeletal 6A | K2C6A |
| Keratin, type II cytoskeletal 6B | K2C6B |
| Keratin, type II cytoskeletal 6C | K2C6C |
| Keratin, type II cytoskeletal 75 | K2C75 |
| Keratin, type II cytoskeletal 79 | K2C79 |
| Keratin, type II cytoskeletal 2 epidermal | K22E |
| UPF0553 protein C9orf64 | CI064 |
| F-actin-capping protein subunit alpha-1 | CAZA1 |
| Keratin, type II cytoskeletal 1 | K2C1 |
| Keratin, type II cytoskeletal 5 | K2C5 |
| Keratin, type II cytoskeletal 6A | K2C6A |
| Keratin, type II cytoskeletal 6B | K2C6B |
| Keratin, type II cytoskeletal 6C | K2C6C |
| Keratin, type II cytoskeletal 75 | K2C75 |
| Keratin, type II cytoskeletal 79 | K2C79 |
| Nicotinate-nucleotide pyrophosphorylase [carboxylating] | NADC |
| Keratin, type II cytoskeletal 5 | K2C5 |
| Keratin, type II cytoskeletal 6A | K2C6A |
| Keratin, type II cytoskeletal 6B | K2C6B |
| Keratin, type II cytoskeletal 6C | K2C6C |
| Keratin, type II cytoskeletal 75 | K2C75 |
| Keratin, type II cytoskeletal 79 | K2C79 |
| Keratin, type II cytoskeletal 2 epidermal | K22E |
| Tubulin beta chain | TBB5 |
| Phosphoglycolate phosphatase | PGP |
| Tubulin beta-2C chain | TBB2C |
| Tubulin beta-3 chain | TBB3 |
| Tubulin beta-2A chain | TBB2A |
| Tubulin beta-2B chain | TBB2B |
| Tubulin beta-6 chain | TBB6 |
| Keratin, type II cytoskeletal 5 | K2C5 |
| Keratin, type II cytoskeletal 6A | K2C6A |
| Keratin, type II cytoskeletal 6B | K2C6B |
| Keratin, type II cytoskeletal 6C | K2C6C |
| Keratin, type II cytoskeletal 75 | K2C75 |
| Keratin, type II cytoskeletal 79 | K2C79 |
| Keratin, type II cytoskeletal 2 epidermal | K22E |
| Annexin A4 | ANXA4 |
| Annexin A1 | ANXA1 |
| UDP-glucose 4-epimerase | GALE |
| Keratin, type II cytoskeletal 1 | K2C1 |
| Serine/threonine-protein phosphatase PP1-gamma catalytic subunit | PP1G |
| Annexin A2 | ANXA2 |
| AH receptor-interacting protein | AIP |
| Keratin, type II cytoskeletal 5 | K2C5 |
| Keratin, type II cytoskeletal 6A | K2C6A |
| Keratin, type II cytoskeletal 6B | K2C6B |
| Keratin, type II cytoskeletal 6C | K2C6C |
| Keratin, type II cytoskeletal 75 | K2C75 |
| Keratin, type II cytoskeletal 79 | K2C79 |
| Keratin, type II cytoskeletal 2 epidermal | K22E |
| Malate dehydrogenase, cytoplasmic | MDHC |
| LIM and SH3 domain protein 1 | LASP1 |
| UDP-glucose 4-epimerase | GALE |
| Keratin, type II cytoskeletal 1 | K2C1 |
| Annexin A2 | ANXA2 |
| Keratin, type I cytoskeletal 9 | K1C9 |
| Annexin A1 | ANXA1 |
| PDZ and LIM domain protein 1 | PDLI1 |
| Annexin A2 | ANXA2 |
| Calponin-2 | CNN2 |
| Glyceraldehyde-3-phosphate dehydrogenase | G3P |
| Keratin, type II cytoskeletal 1 | K2C1 |
| BRCA1/BRCA2-containing complex subunit 3 | BRCC3 |
| DnaJ homolog subfamily C member 8 | DNJC8 |
| Annexin A5 | ANXA5 |
| Keratin, type II cytoskeletal 1 | K2C1 |
| Glyceraldehyde-3-phosphate dehydrogenase | G3P |
| Thiosulfate sulfurtransferase | THTR |
| Pyrroline-5-carboxylate reductase 1, mitochondrial | P5CR1 |
| Pyrroline-5-carboxylate reductase 2 | P5CR2 |
| Annexin A4 | ANXA4 |
| Proteasome activator complex subunit 3 | PSME3 |
| Keratin, type II cytoskeletal 1 | K2C1 |
| Serine/threonine-protein phosphatase 6 catalytic subunit | PPP6 |
| Glyceraldehyde-3-phosphate dehydrogenase | G3P |
| Thiosulfate sulfurtransferase | THTR |
| Coatomer subunit epsilon | COPE |
| 40S ribosomal protein S3 | RS3 |
| Annexin A5 | ANXA5 |
| Actin, cytoplasmic 1 | ACTB |
| Actin, cytoplasmic 2 | ACTG |
| Chloride intracellular channel protein 1 | CLIC1 |
| Beta-actin-like protein 2 | ACTBL |
| EF-hand domain-containing protein D2 | EFHD2 |
| Keratin, type II cytoskeletal 1 | K2C1 |
| U1 small nuclear ribonucleoprotein A | SNRPA |
| Keratin, type II cytoskeletal 5 | K2C5 |
| Keratin, type II cytoskeletal 6A | K2C6A |
| Keratin, type II cytoskeletal 6B | K2C6B |
| Keratin, type II cytoskeletal 6C | K2C6C |
| Keratin, type II cytoskeletal 75 | K2C75 |
| Keratin, type II cytoskeletal 79 | K2C79 |
| Proteasome activator complex subunit 3 | PSME3 |
| Toll-interacting protein | TOLIP |
| 14-3-3 protein epsilon | 1433E |
| 14-3-3 protein theta | 1433T |
| Electron transfer flavoprotein subunit alpha, mitochondrial | ETFA |
| Voltage-dependent anion-selective channel protein 2 | VDAC2 |
| V-type proton ATPase subunit E 1 | VATE1 |
| Keratin, type II cytoskeletal 1 | K2C1 |
| Keratin, type II cytoskeletal 5 | K2C5 |
| Keratin, type II cytoskeletal 6A | K2C6A |
| Keratin, type II cytoskeletal 6B | K2C6B |
| Keratin, type II cytoskeletal 6C | K2C6C |
| Keratin, type II cytoskeletal 75 | K2C75 |
| Keratin, type II cytoskeletal 79 | K2C79 |
| Keratin, type II cytoskeletal 2 epidermal | K22E |
| Proteasome assembly chaperone 1 | PSMG1 |
| Glucosamine-6-phosphate isomerase 1 | GNPI1 |
| Coiled-coil domain-containing protein 5 | CCDC5 |
| Keratin, type II cytoskeletal 1 | K2C1 |
| Isochorismatase domain-containing protein 1 | ISOC1 |
| V-type proton ATPase subunit E 1 | VATE1 |
| Syntenin-1 | SDCB1 |
| Keratin, type II cytoskeletal 1 | K2C1 |
| Electron transfer flavoprotein subunit alpha, mitochondrial | ETFA |
| Protein angel homolog 1 | ANGE1 |
| Keratin, type II cytoskeletal 1 | K2C1 |
| Peroxiredoxin-6 | PRDX6 |
| Fumarylacetoacetate hydrolase domain-containing protein 1 | FAHD1 |
| Keratin, type II cytoskeletal 1 | K2C1 |
| Proteasome subunit alpha type-3 | PSA3 |
| Keratin, type I cytoskeletal 18 | K1C18 |
| Keratin, type II cytoskeletal 1 | K2C1 |
| Fumarylacetoacetate hydrolase domain-containing protein 1 | FAHD1 |
| Carbonic anhydrase 2 | CAH2 |
| Glutathione S-transferase P | GSTP1 |
| Glutathione S-transferase P | GSTP1 |
| Proteasome subunit beta type-3 | PSB3 |
| High mobility group protein B1 | HMGB1 |
| Putative high mobility group protein 1-like 1 | HMGL1 |
| High mobility group protein 1-like 10 | HMGLX |
| Glutathione S-transferase P | GSTP1 |
| Oligoribonuclease, mitochondrial | ORN |
| Proteasome subunit beta type-4 | PSB4 |
| Uncharacterized protein C9orf142 | CI142 |
| Gamma-glutamyltranspeptidase 1 | GGT1 |
| Gamma-glutamyltranspeptidase 2 | GGT2 |
| Putative gamma-glutamyltranspeptidase 3 | GGT3 |
| Gamma-glutamyltransferase light chain 2 | GGTL2 |
| Putative gamma-glutamyltransferase light chain 3 | GGTL3 |
| Proteasome subunit beta type-3 | PSB3 |
| Uncharacterized protein C9orf142 | CI142 |
| Glutathione S-transferase P | GSTP1 |
| Cathepsin B | CATB |
| Glutathione S-transferase P | GSTP1 |
| COMM domain-containing protein 3 | COMD3 |
| Superoxide dismutase [Mn], mitochondrial | SODM |
| Glutathione S-transferase P | GSTP1 |
| Superoxide dismutase [Mn], mitochondrial | SODM |
| RNA-binding protein 8A | RBM8A |
| Phosphatidylethanolamine-binding protein 1 | PEBP1 |
| Nucleoside diphosphate kinase A | NDKA |
| 60S ribosomal protein L11 | RL11 |
| 60S ribosomal protein L12 | RL12 |
| Uncharacterized protein C6orf125 | CF125 |
| Keratin, type I cytoskeletal 10 | K1C10 |
| Keratin, type II cytoskeletal 6A | K2C6A |
| Keratin, type II cytoskeletal 6B | K2C6B |
| Keratin, type II cytoskeletal 6C | K2C6C |
| Profilin-1 | PROF1 |
| Keratin, type II cytoskeletal 1 | K2C1 |
| Histidine triad nucleotide-binding protein 1 | HINT1 |
| Heat shock protein 105 kDa | HS105 |
| Ubiquitin carboxyl-terminal hydrolase 5 | UBP5 |
| Heat shock protein HSP 90-alpha | HS90A |
| Acylamino-acid-releasing enzyme | ACPH |
| Keratin, type II cytoskeletal 1 | K2C1 |
| Ezrin | EZRI |
| Radixin | RADI |
| Rootletin | CROCC |
| Keratin, type II cytoskeletal 1 | K2C1 |
| Dihydrolipoyllysine-residue acetyltransferase component of pyruvate dehydrogenase complex, mitochondrial | ODP2 |
| Keratin, type II cytoskeletal 5 | K2C5 |
| Keratin, type II cytoskeletal 6A | K2C6A |
| Keratin, type II cytoskeletal 6B | K2C6B |
| Keratin, type II cytoskeletal 6C | K2C6C |
| Keratin, type II cytoskeletal 75 | K2C75 |
| Keratin, type II cytoskeletal 79 | K2C79 |
| Keratin, type II cytoskeletal 2 epidermal | K22E |
| Stress-70 protein, mitochondrial | GRP75 |
| Keratin, type II cytoskeletal 1 | K2C1 |
| Heterogeneous nuclear ribonucleoprotein K | HNRPK |
| 60 kDa heat shock protein, mitochondrial | CH60 |
| Stress-70 protein, mitochondrial | GRP75 |
| Bifunctional purine biosynthesis protein PURH | PUR9 |
| Eukaryotic initiation factor 4A-I | IF4A1 |
| Eukaryotic initiation factor 4A-II | IF4A2 |
| Keratin, type II cytoskeletal 1 | K2C1 |
| Poly(U)-binding-splicing factor PUF60 | PUF60 |
| Polypyrimidine tract-binding protein 1 | PTBP1 |
| Pyruvate kinase isozymes M1/M2 | KPYM |
| Coatomer subunit delta | COPD |
| Keratin, type II cytoskeletal 1 | K2C1 |
| UDP-N-acetylhexosamine pyrophosphorylase | UAP1 |
| T-complex protein 1 subunit theta | TCPQ |
| 60 kDa heat shock protein, mitochondrial | CH60 |
| T-complex protein 1 subunit epsilon | TCPE |
| Splicing factor 3A subunit 3 | SF3A3 |
| Keratin, type II cytoskeletal 1 | K2C1 |
| V-type proton ATPase subunit B, brain isoform | VATB2 |
| Protein disulfide-isomerase A3 | PDIA3 |
| Adenylyl cyclase-associated protein 1 | CAP1 |
| T-complex protein 1 subunit eta | TCPH |
| Dihydrolipoyl dehydrogenase, mitochondrial | DLDH |
| Protein disulfide-isomerase A3 | PDIA3 |
| Retinal dehydrogenase 1 | AL1A1 |
| Eukaryotic translation initiation factor 5 | IF5 |
| Retinal dehydrogenase 1 | AL1A1 |
| Cytosol aminopeptidase | AMPL |
| Dynein heavy chain 11, axonemal | DYH11 |
| Seryl-tRNA synthetase, mitochondrial | SYSM |
| Glutamate dehydrogenase 1, mitochondrial | DHE3 |
| Fascin | FSCN1 |
| Keratin, type II cytoskeletal 1 | K2C1 |
| Lupus La protein | LA |
| Keratin, type II cytoskeletal 1 | K2C1 |
| Actin, cytoplasmic 1 | ACTB |
| Actin, cytoplasmic 2 | ACTG |
| Beta-actin-like protein 2 | ACTBL |
| Keratin, type II cytoskeletal 1 | K2C1 |
| Tubulin alpha-1A chain | TBA1A |
| Tubulin alpha-1B chain | TBA1B |
| Tubulin alpha-1C chain | TBA1C |
| Creatine kinase B-type | KCRB |
| Adenosylhomocysteinase | SAHH |
| Actin, cytoplasmic 2 | ACTG |
| Beta-actin-like protein 2 | ACTBL |
| Keratin, type II cytoskeletal 1 | K2C1 |
| Actin, cytoplasmic 1 | ACTB |
| Actin, cytoplasmic 2 | ACTG |
| Beta-actin-like protein 2 | ACTBL |
| Keratin, type II cytoskeletal 1 | K2C1 |
| 26S protease regulatory subunit 8 | PRS8 |
| Keratin, type II cytoskeletal 1 | K2C1 |
| 6-phosphogluconate dehydrogenase, decarboxylating | 6PGD |
| Actin, cytoplasmic 2 | ACTG |
| Actin, cytoplasmic 1 | ACTB |
| Creatine kinase B-type | KCRB |
| Beta-actin-like protein 2 | ACTBL |
| Keratin, type II cytoskeletal 1 | K2C1 |
| PDZ domain-containing protein GIPC1 | GIPC1 |
| Acetyl-CoA acetyltransferase, cytosolic | THIC |
| Keratin, type II cytoskeletal 1 | K2C1 |
| Eukaryotic translation initiation factor 3 subunit H | EIF3H |
| Phosphoserine aminotransferase | SERC |
| Dynein heavy chain domain-containing protein 2 | DNHD2 |
| Sialic acid synthase | SIAS |
| Alcohol dehydrogenase [NADP+] | AK1A1 |
| Serine-threonine kinase receptor-associated protein | STRAP |
| Nucleophosmin | NPM |
| Protein CDV3 homolog | CDV3 |
| Serine/threonine-protein phosphatase PP1-alpha catalytic subunit | PP1A |
| Annexin A1 | ANXA1 |
| Glyceraldehyde-3-phosphate dehydrogenase | G3P |
| 14-3-3 protein epsilon | 1433E |
| Electron transfer flavoprotein subunit alpha, mitochondrial | ETFA |
| Isochorismatase domain-containing protein 1 | ISOC1 |
| Fumarylacetoacetate hydrolase domain-containing protein 1 | FAHD1 |
| Methylcrotonoyl-CoA carboxylase beta chain, mitochondrial | MCCB |
| Tyrosyl-tRNA synthetase, cytoplasmic | SYYC |
| Septin-2 | DIFF6 |
| Transaldolase | TALDO |
| PDZ and LIM domain protein 1 | PDLI1 |
| Tropomyosin alpha-4 chain | TPM4 |
| Triosephosphate isomerase | TPIS |
| Keratin, type I cytoskeletal 18 | K1C18 |
| Keratin, type I cytoskeletal 19 | K1C19 |
| Lamin-B2 | LMNB2 |


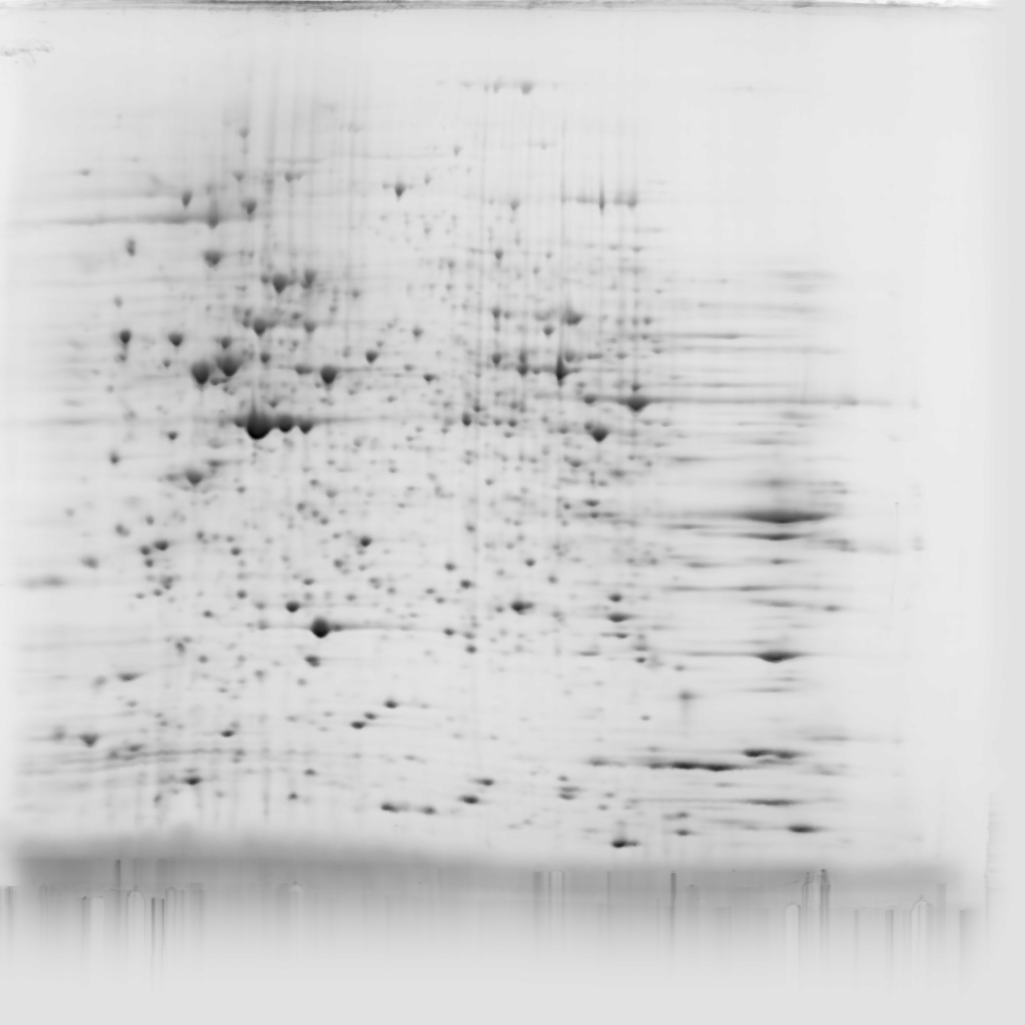


1

2

4

5

6

3

7

9

8

15

11

12

10

17

16

19

13

14

18

20

21

22

25

26

27

24

30

41

32

37

28

42

40

55

34

31

44

54

33

29

35

36

38

39

43

48

50

45

47

52

49

56

62

61

53

51

58

63

59

60

65

66

68

64

69

76

72

70

82

77

75

79

81

83

98

91

92

90

95

87

93

89

84

88

97

100

99

71

73

78

80

85

96

94

23

46

57

67

86

74

Approximate molecular

weight, kDa

pH 3

pH 10

200

10

50

**Proteomics synthetic gel**

**Comparative Canonical Pathway Data**

| **Canonical pathway in RNA-seq analysis only** | **Canonical pathway in microarray experiment only** |
| --- | --- |
|  |  |
| 14-3-3-mediated Signaling | Alanine and Aspartate Metabolism |
| 4-1BB Signaling in T Lymphocytes | Aminoacyl-tRNA Biosynthesis |
| Actin Cytoskeleton Signaling | Arginine and Proline Metabolism |
| Activation of IRF by Cytosolic Pattern Recognition Receptors | Cell Cycle: G2/M DNA Damage Checkpoint Regulation |
| Acute Myeloid Leukemia Signaling | Cyanoamino Acid Metabolism |
| Apoptosis Signaling | Fatty Acid Metabolism |
| April Mediated Signaling | Glycine, Serine and Threonine Metabolism |
| Axonal Guidance Signaling | Glycolysis/Gluconeogenesis |
| B Cell Activating Factor Signaling | Glycosaminoglycan Degradation |
| B Cell Receptor Signaling | Glycosphingolipid Biosynthesis - Neolactoseries |
| Basal Cell Carcinoma Signaling | Histidine Metabolism |
| BMP signaling pathway | LPS/IL-1 Mediated Inhibition of RXR Function |
| Cardiomyocyte Differentiation via BMP Receptors | NF-κB Signaling |
| CD27 Signaling in Lymphocytes | N-Glycan Degradation |
| CD40 Signaling | One Carbon Pool by Folate |
| Ceramide Signaling | p38 MAPK Signaling |
| Cholecystokinin/Gastrin-mediated Signaling | Parkinson's Signaling |
| Circadian Rhythm Signaling | Propanoate Metabolism |
| CXCR4 Signaling | PTEN Signaling |
| Dendritic Cell Maturation | Pyruvate Metabolism |
| EGF Signaling | Sphingolipid Metabolism |
| Erythropoietin Signaling | TGF-β Signaling |
| Factors Promoting Cardiogenesis in Vertebrates | Urea Cycle and Metabolism of Amino Groups |
| Glucocorticoid Receptor Signaling | Valine, Leucine and Isoleucine Degradation |
| GNRH Signaling | Total = 24 |
| G-Protein Coupled Receptor Signaling |  |
| Gα12/13 Signaling |  |
| Hepatic Fibrosis / Hepatic Stellate Cell Activation |  |
| HGF Signaling |  |
| HMGB1 Signaling |  |
| Human Embryonic Stem Cell Pluripotency |  |
| Hypoxia Signaling in the Cardiovascular System |  |
| IL-17 Signaling |  |
| IL-9 Signaling |  |
| ILK Signaling |  |
| Induction of Apoptosis by HIV1 |  |
| LPS-stimulated MAPK Signaling |  |
| Lymphotoxin β Receptor Signaling |  |
| Lysine Biosynthesis |  |
| MIF Regulation of Innate Immunity |  |
| Molecular Mechanisms of Cancer |  |
| NF-κB Activation by Viruses |  |
| NF-κB Signaling |  |
| PDGF Signaling |  |
| Production of Nitric Oxide and Reactive Oxygen Species in Macrophages |  |
| Prolactin Signaling |  |
| RAR Activation |  |
| Relaxin Signaling |  |
| Renal Cell Carcinoma Signaling |  |
| Role of NANOG in Mammalian Embryonic Stem Cell Pluripotency |  |
| SAPK/JNK Signaling |  |
| Semaphorin Signaling in Neurons |  |
| Small Cell Lung Cancer Signaling |  |
| Sphingosine-1-phosphate Signaling |  |
| Thrombopoietin Signaling |  |
| Thyroid Cancer Signaling |  |
| Tight Junction Signaling |  |
| Toll-like Receptor Signaling |  |
| TR/RXR Activation |  |
| Type II Diabetes Mellitus Signaling |  |
| Virus Entry via Endocytic Pathways |  |
| Total = 61 |  |

| **Canonical pathway common in microarray and RNA-seq experiment** |
| --- |
|  |
| Acute Phase Response Signaling |
| Aryl Hydrocarbon Receptor Signaling |
| ERK/MAPK Signaling |
| Hepatic Cholestasis |
| IL-10 Signaling |
| IL-6 Signaling |
| p53 Signaling |
| PPAR Signaling |
| PXR/RXR Activation |
| TGF-β Signaling |
| PPARα/RXRα Activation |
| Total = 11 |

| **Canonical pathway in proteomics experiment only** | **Canonical pathway common in RNA-seq and proteomics experiment** |
| --- | --- |
|  |  |
| Agrin Interactions at Neuromuscular Junction | Caveolar-mediated Endocytosis |
| Arachidonic Acid Metabolism | Cellular Effects of Sildenafil (Viagra) |
| Glutathione Metabolism | Germ Cell-Sertoli Cell Junction Signaling |
| Mechanisms of Viral Exit from Host Cells | Integrin Signaling |
| VEGF Signaling | Leukocyte Extravasation Signaling |
| Total = 5 | NRF2-mediated Oxidative Stress Response |
|  | Total = 6 |

**Microarray Data Biofunctions**

| Endocrine System Disorders | diabetes | 1.47E-05 | ANXA1, B2M, BTG2, CD38, CPT1A, CTSL1, DPP4, DUSP1, GBP2, HMGCS2, HRSP12, ICA1, ICAM1, IL18, INSR, IRF1, IRS2, JUN, KLF4, KLF11, MT1E, MYC, NCF2, PDE3A, PDE5A, PLAGL1, PTTG1, RHOB, SERPINA3, SERPINE1, SOCS1, SPP1, SUMO4 | |
| --- | --- | --- | --- | --- |
| Lipid Metabolism | metabolic process of lipid | 6.34E-05 | ACADSB, ACOT9, ACSL1, ACSS2, ADM, AKR1C2, ANXA1, APOA1, APOM, ASAH1, B3GNT5, CAV1, CPT1A, CYP1A1, DGKK, DLAT, EHHADH, FABP1, FABP3, FABP5, HEXA, HEXB, HMGCS2, HPGD, IKBKB, KNG1 (includes EG:3827), LASS5, LPIN1, MLXIPL, NPPA, PDE3A, PLA1A, PLA2G4C (includes EG:8605), PNPLA8, PRKAR2B, SC5DL, SH3GLB1, SLC27A2, SMPD1, SNCA, TPP1 | |
| Lipid Metabolism | metabolic process of fatty acid | 2.37E-03 | ACADSB, ACOT9, ACSL1, ACSS2, CAV1, CPT1A, DLAT, EHHADH, FABP5, HMGCS2, PNPLA8, PRKAR2B, SC5DL, SLC27A2 | |
| Lipid Metabolism | quantity of lipid | 7.51E-05 | ABCB1, ABCG2, ACSL1, ADFP, APOA1, ASAH1, CASP8, CAV1, CD38, CPT1A, CSF1, EDN1, EPHX2, FABP1, FABP3, HPGD, IFRD1, IL8, IL18, INSR, IRS2, KNG1 (includes EG:3827), LPIN1, LPP, MYC, NPC1, NPPA, PDGFRA, PNPLA8, PON1, PPARGC1A, RORA, SAA4, SLC3A2, SMPD1, SNCA, VLDLR | |
| Lipid Metabolism | quantity of phospholipid | 1.58E-03 | APOA1, CAV1, CD38, CSF1, IL8, INSR, KNG1 (includes EG:3827), LPP, NPC1, PDGFRA, PNPLA8, PON1, SLC3A2, SMPD1, SNCA, VLDLR | |
| Lipid Metabolism | metabolism of lipid | 4.77E-04 | ACADSB, ACOT9, ACSL1, ACSS2, AKR1C2, ANXA1, APOA1, APOM, ASAH1, B3GNT5, CAV1, CPT1A, CYP1A1, DGKK, DLAT, EHHADH, FABP1, FABP3, FABP5, HEXA, HEXB, HMGCS2, HPGD, KNG1 (includes EG:3827), LASS5, LPIN1, PDE3A, PLA1A, PLA2G4C (includes EG:8605), PNPLA8, PRKAR2B, SC5DL, SH3GLB1, SLC27A2, SMPD1, SNCA, TPP1 | |
| Lipid Metabolism | metabolism of fatty acid | 3.30E-03 | ACADSB, ACOT9, ACSL1, ACSS2, CAV1, CPT1A, DLAT, EHHADH, HMGCS2, PNPLA8, PRKAR2B, SC5DL, SLC27A2 | |
| Lipid Metabolism | hydrolysis of lipid | 8.17E-04 | ADFP, ADRA2C, ASAH1, CASP8, EDN1, F2RL1, HEXA, HEXB, IL8, KNG1 (includes EG:3827), LPP, PLA1A, PLA2G4C (includes EG:8605), PON1, SMPD1, SNCA, TMEM55B | |
| Lipid Metabolism | hydrolysis of gangliotriaosylceramide II3-sulfate | 2.91E-03 | HEXA, HEXB |  |
| Lipid Metabolism | hydrolysis of lactosylceramide II3-sulfate | 2.91E-03 | HEXA, HEXB |  |
| Lipid Metabolism | storage of lipid | 1.11E-03 | ADFP, APOA1, CAV1, HEXA, HEXB, SMPD1 | |
| Lipid Metabolism | lipolysis | 1.36E-03 | ADM, ANXA1, CIDEC, FABP5, IKBKB, NPPA, NR4A1, PRKAR2B | |
| Lipid Metabolism | oxidation of lipid | 1.74E-03 | ACADSB, ACSL1, CPT1A, EHHADH, FABP1, FABP3, HPGD, INSR, LCAT, LPIN1, PNPLA8, PPARGC1A, SLC27A2 | |
| Lipid Metabolism | release of phosphatidic acid | 2.05E-03 | ABCB1, APOA1, EDN1, PLA1A | |
| Lipid Metabolism | release of thromboxane | 2.91E-03 | EDN1, NPPA |  |
| Lipid Metabolism | release of thromboxane B2 | 2.91E-03 | EDN1, NPPA |  |
| Lipid Metabolism | transport of cholesterol | 2.21E-03 | ABCB1, APOA1, CAV1, LCAT, NPC1L1, SMPD1 | |
| Lipid Metabolism | transport of lipid | 2.45E-03 | ABCB1, ABCC3, ABCG2, ACSL1, ADFP, APOA1, FABP1, FABP3, NPC1, NPC2, NPC1L1 | |
| Lipid Metabolism | transport of glucocorticoid | 2.91E-03 | ABCB1, SERPINA6 | |

**RNA-Seq Data Biofunctions**

| Category | Function Annotation | P-value | Molecules |
| --- | --- | --- | --- |
| Metabolic Disease | diabetes | 2.62E-05 | ABLIM2, AFF3, ANK2, ANKS1B, ANO4, ANXA1, APOM, B2M, BACH2 (includes EG:60468), BDNF, BTG2, BTN3A1, CACNA1A, CDH6, CDH19, CEBPD, CHD5, CHST3, CLK4, CORIN, CPEB4, CREB5, CSF1, CSF3R, CTSL1, CUX2, CYTSB, DAAM1, DAPK2 (includes EG:23604), DCDC2, DDR1, DLC1, DPP4, DTNA, DUSP1, EDNRB, EFEMP1, EPB41L1, EPHA5, FAM125B, FAM129A, FAM38B, FGF12, FHIT, FHOD3 (includes EG:80206), FLVCR2, FUT8, GBP2, GLIPR2, GLIS3, GLP1R, GPC5, GPR158, GPR161, GRK5, HDAC9 (includes EG:9734), HEG1, HMGCS2, HSPA1L, ICAM1, IFIH1, IFIT2, IFITM3, IL8, IL18, INSR, IRF1, IRS2, ITPR3, JAG1, JUN, KIAA1609, KLF4, KLF11, KLHL5, KRT222P, L3MBTL4 (includes EG:91133), LAMA3, LAMC2, LBH, LEPREL1, MAP3K1, MAPK8IP1, MAST4, MATN2, MBOAT1, METTL7A, MICAL2, MOBKL2B, MYC, MYH9, MYOF, MYOM1, NBEA (includes EG:26960), NCF2, NCK2, NFKBIA, NMT2, NPTXR, NRG3, NRP2, OCA2, ODZ2, OPN1SW, PALLD, PAPPA, PARD3B, PARP12, PARVA, PDE3A, PDE5A, PDE9A, PDGFRL, PHLDB1, PLXNC1, PPAP2A, PRICKLE1, PRICKLE2, PSMG4 (includes EG:389362), RAP1GAP (includes EG:5909), RAPGEF4, RFTN1, ROBO2, ROS1, SAMD12, SAMD4A, SEC31B, SERPINA3, SH3YL1, SLC44A3, SLC6A4, SPTBN1, SPTBN5, SRGAP1, SSTR2, SVIL, SYNE2, TAP1, TAP2, TBX15, TEAD1, TMEFF1, TMEM2, TNFRSF11B, TRIM9, TSHZ2, UHRF2, VEPH1, WWC1, YPEL2, ZNF350 |
| Carbohydrate Metabolism | metabolism of D-glucose | 4.59E-04 | BDNF, CACNA1A, DLK1, G6PC, HBEGF, IRS2, MYC, PDK1, PDK4, SLC37A4 |
| Carbohydrate Metabolism | accumulation of carbohydrate | 6.70E-04 | ABCC3, ADCYAP1, APOA1, AQP7, BMP2, CX3CL1, CXCL1, EDNRB, GRK5, IL8, INSR, MYH9, SLC37A4 |
| Carbohydrate Metabolism | hydrolysis of glucose-6-phosphate | 1.88E-03 | G6PC, SLC37A4 |
| Carbohydrate Metabolism | transport of glucose-6-phosphate | 5.49E-03 | G6PC, SLC37A4 |
| Carbohydrate Metabolism | uptake of glucose-6-phosphate | 5.49E-03 | G6PC, SLC37A4 |
| Small Molecule Biochemistry | metabolism of D-glucose | 4.59E-04 | BDNF, CACNA1A, DLK1, G6PC, HBEGF, IRS2, MYC, PDK1, PDK4, SLC37A4 |
| Small Molecule Biochemistry | production of leukotriene B4 | 9.11E-04 | IL8, IL18, OPRL1, STAT5A |
| Small Molecule Biochemistry | hydrolysis of glucose-6-phosphate | 1.88E-03 | G6PC, SLC37A4 |
| Small Molecule Biochemistry | hydrolysis of cyclic GMP | 2.50E-03 | PDE1A, PDE5A, PDE9A |
| Small Molecule Biochemistry | uptake of amino acids | 3.45E-03 | ADCYAP1, BDNF, CLN8, SLC1A3, SLC3A2, SLC6A8, STX1A |
| Small Molecule Biochemistry | uptake of glucose-6-phosphate | 5.49E-03 | G6PC, SLC37A4 |
| Small Molecule Biochemistry | quantity of acylglycerol | 5.22E-03 | ADCYAP1, ADFP, APOA1, APOA5, AQP7, CAV1, DAGLA, DLK1, FASN, FES, INSR, IRS2, MYC, NQO1, VLDLR |
| Small Molecule Biochemistry | transport of glucose-6-phosphate | 5.49E-03 | G6PC, SLC37A4 |
| Lipid Metabolism | production of leukotriene B4 | 9.11E-04 | IL8, IL18, OPRL1, STAT5A |
| Lipid Metabolism | quantity of acylglycerol | 5.22E-03 | ADCYAP1, ADFP, APOA1, APOA5, AQP7, CAV1, DAGLA, DLK1, FASN, FES, INSR, IRS2, MYC, NQO1, VLDLR |
| Molecular Transport | quantity of lactic acid | 2.49E-03 | CEBPD, HIF1A, MYC, NQO1 |
| Molecular Transport | quantity of calcium | 2.94E-03 | ADCYAP1, BDNF, CAV1, CCL20, CCR1, CD59, CSF1, CX3CL1, EDNRB, F2RL1, FOS, GRK5, HOXA3 (includes EG:3200), HSPA5, ICAM1, IL8, INSR, ITPR3, LTBP1, LYZ, MCAM, MYC, NPPB, OXTR, P2RY2, PIK3CD, PRNP, RASGRP3, S1PR3, TNFRSF11B, ZP3 |
| Molecular Transport | quantity of acylglycerol | 5.22E-03 | ADCYAP1, ADFP, APOA1, APOA5, AQP7, CAV1, DAGLA, DLK1, FASN, FES, INSR, IRS2, MYC, NQO1, VLDLR |
| Molecular Transport | uptake of amino acids | 3.45E-03 | ADCYAP1, BDNF, CLN8, SLC1A3, SLC3A2, SLC6A8, STX1A |
| Molecular Transport | uptake of glucose-6-phosphate | 5.49E-03 | G6PC, SLC37A4 |
| Molecular Transport | transport of glucose-6-phosphate | 5.49E-03 | G6PC, SLC37A4 |
